# Development of an SI traceable value assigned amino acid matrix-matched material to underpin European external quality assessment

**DOI:** 10.1007/s00216-025-05793-4

**Published:** 2025-02-24

**Authors:** Emily Whyte, Rachel Carling, Simon Cowen, Patrick Sears, Chris Hopley

**Affiliations:** 1 LGC, Teddington, Middlesex TW11 0LY UK; 2 Faculty of Engineering and Physical Sciences, Univeristy of Surrey, Surrey, GU2 7XH UK; 3 Biochemical Sciences, Synnovis, Guys & St Thomas’ NHSFT, London, SE1 7EH UK; 4 European Research Network for Evaluation and Improvement of Screening, Diagnosis and Treatment of Inherited Disorders of Metabolism, Sheepbridge Centre, Chesterfield, Derbyshire S41 9RX UK; 5 GKT School Medical Education, Kings College London, Strand, London, WC2R 2LS UK

**Keywords:** Phenylketonuria, Amino acids, Plasma, External quality assessment, Standardisation, Certified reference material

## Abstract

Plasma amino acid analysis plays a vital role in monitoring inherited metabolic diseases (IMDs), such as phenylketonuria (PKU), where dietary therapy is the cornerstone of management and patients are regularly monitored using blood or plasma phenylalanine throughout life. Recent findings from external quality assessment (EQA) schemes demonstrate large variability between results from different clinical laboratories. EQA schemes are hampered by factors including suboptimal sample type and frequency of distributions. Aiming to harmonise and standardise EQA schemes, this paper describes value assignment of an SI traceable matrix-matched amino acid material, and its use in an interlaboratory comparison (ERNDIM QAA-2106 study) that included 89 clinical laboratories from throughout Europe. The outcomes of the interlaboratory comparison are valuable in enabling clinical laboratories to refine their methodologies and, in a longer term, will lead to enhanced accuracy of results in clinical measurements and, ultimately, improved patient care in IMD management. A frozen, pooled human plasma sample was distributed to participating laboratories (*n* = 88). The sample was value assigned for phenylalanine by the National Measurement Laboratory (NML) traceable to the international system of units (SI), at approximately the target value for dietary monitoring of phenylketonuria (360 µmol/L). Participants used their routine method of analysis to measure 24 amino acids in triplicate and results were compared against desirable analytical performance. Mean intra- and inter-laboratory variability were acceptable for 20/20 and 19/20 amino acids respectively. Mean bias relative to the consensus values was acceptable for 17/20 amino acids. Overall performance of phenylalanine was acceptable; however, 15/87 laboratories failed to meet acceptable imprecision and 8/87 had unacceptable bias; additionally, an overall negative bias for phenylalanine measurement was identified. This study demonstrates the importance of ensuring a matrix material with an assigned value of the analyte(s) of interest (traceable to the SI) is available for regular EQA. The significant bias in the routine measurement of phenylalanine identified has the potential to impact disease management, which aims to control blood phenylalanine concentrations for preventing adverse neurological outcomes.

**Supplementary Information:**

The online version contains supplementary material available at 10.1007/s00216-025-05793-4.

## Introduction

Plasma amino acid analysis is a key measurement for the diagnosis and monitoring of inherited metabolic diseases such as phenylketonuria (PKU), maple syrup urine disease (MSUD) and homocystinuria (HCU). A comprehensive amino acid profile will typically quantify around 25 different analytes in a single plasma sample [1]. Amino acid analysis is usually provided by a specialist laboratory, and it is generally acknowledged that both technical and clinical expertise are required to deliver a safe and robust clinical service. Historically, analysis of plasma amino acids was carried out almost exclusively by ion exchange chromatography (IEC) with post column derivatisation and UV detection. However, data from the European Research Network for the Evaluation and Improvement of Screening, diagnosis and Treatment of Inherited disorders of Metabolism (ERNDIM) Quantitative Amino Acids in Serum (QTAS) External Quality Assessment (EQA) scheme shows that the proportion of participants using IEC has reduced from 86% (2001) to 46% (2022) [2], whilst LC–MS/MS usage has increased proportionally from 0% (2001) to 33% (2022) [3]. Although IEC benefits from repeatability and a broad dynamic range, LC–MS/MS provides superior selectivity and a rapid analysis time in comparison to IEC [4, 5].

EQA schemes play an important role for monitoring and improving the quality of laboratory testing. The largest scheme is the ERNDIM QTAS EQA scheme which had 310 participating laboratories from 54 different countries in 2022. However, utility of the scheme is limited by the small number of samples distributed each year; eight samples are supplied annually which are in fact duplicate pairs [3]. An alternative amino acid EQA scheme, Birmingham Quality Quantitative Amino Acid scheme, distributes three serum specimens, 11 times per year, but this scheme only includes a subset of amino acids (isoleucine, leucine, phenylalanine, tyrosine and valine) whereas the ERNDIM QTAS scheme includes > 20 amino acids [6]. Other clinical chemistry schemes, immunosuppressants for example, typically supply multiple samples per month throughout the year [7–9]. For clinical laboratories, regular and frequent distribution of EQA samples is advantageous, as it helps to monitor lot-to-lot variation in reagents and calibrators [10].

The results of the ERNDIM QTAS scheme are scored against a derivation of the All Laboratory Trimmed Mean (ALTM), a calculated result based entirely on participants’ results that may not be consistent with an SI traceable reference value. This approach can cause false reassurance since laboratories may be in agreement with the ALTM yet still be inaccurate. Conversely, there is a risk that laboratories who are accurate, may start investigating a non-existent bias. Both outcomes are unsatisfactory and without comparison to an SI traceable value, this approach is unlikely to help individual laboratories improve their accuracy [11, 12]. Data from the ERNDIM QTAS scheme indicates only a small bias in absolute value compared to the gravimetric concentration (typically around 90—95% recovery for most amino acids), which is encouraging. However, there is a large variance in results between laboratories; 8–50% dependent on the amino acid. ERNDIM QTAS studies are conducted using stripped, lyophilised serum that has been enriched with the amino acids required for the scheme. It is therefore unclear whether these discrepancies are a result of laboratory measurements or reflect a bias from extraction efficiencies due to the sample format.

Currently, there is no amino acid EQA scheme available that is underpinned by a matrix material with an assigned value that is determined traceable to the international system of units (SI). Whilst absolute accuracy of plasma amino acid results is of less importance for diagnosis, it is vital for the monitoring of patients with inherited metabolic disorders [13]. An SI traceable, matrix-matched amino acid certified reference material (CRM) would help standardise an EQA scheme by providing comparison of results against a true value. Commercially available CRMs with SI traceable values for amino acids in plasma include NIST SRM 1950 and KRISS CRM 111–01–019. However, availability of such materials is limited, primarily due to the challenges and costs associated with their production, which at best constrains the frequency of their use in EQA schemes, and at worst prevents it. This poses a challenge for clinical laboratories and the impact on inter-laboratory harmonisation and standardisation of results has been highlighted by recent reports from the ERNDIM QTAS scheme [4].

This paper describes the development and use of an SI traceable value assigned material in an interlaboratory comparison (ERNDIM QAA-2106 study) for amino acids in plasma. To achieve this, the UK National Measurement laboratory (NML), hosted at LGC, in collaboration with ERNDIM and Synnovis (Guys and St Thomas’ NHSFT, London) produced a frozen, pooled human plasma sample that was more analogous to a patient specimen than the material distributed by the ERNDIM QTAS scheme. Additionally, phenylalanine concentration was determined using SI traceable isotope dilution (IDMS) methodology developed by the NML under ISO/IEC 17025 scope. Stability and homogeneity of the material were determined using the same high-accuracy IDMS methodology for traceable value assignment to make sure that the material was fit for purpose within the duration of the study. Phenylalanine was chosen for value assignment in the study material because management of phenylketonuria (PKU, OMIM 261600) relies upon life-long monitoring of phenylalanine in blood or plasma [14]. Comparability of results independent of time, place and measurement procedure is important, particularly when an individual patient’s test results are compared with clinical decision points described in evidence-based clinical practice guidelines [13, 15–17]. It is therefore crucial that laboratory results are accurate and reproducible [13]. Using this approach, ERNDIM QAA-2106 study participant performance was for the first time compared to an absolute traceable value with the highest confidence.

## Methods

### Participants

Participants for the ERNDIM QAA-2106 study were recruited by letter of invitation sent to all laboratories participating in the QTAS scheme that were based in Europe (*n* = 161). A total of 89 laboratories agreed to participate in the scheme and 89 sets of results were received from 88 laboratories. Participants used their routine method of analysis which included the following techniques: IEC, LC–MS/MS, HPLC and one laboratory reported results from NMR analysis.

### Study sample

A pooled human plasma (lithium heparin) material was sourced and prepared by Maine Standards, MA, USA. The plasma was enriched with phenylalanine and sub-sampled into 1.2 mL units (*n* = 1000). Phenylalanine enrichment was approximately equivalent to the target value for dietary monitoring of PKU (360 µmol/L) [16]. Homogeneity and stability data were collected at various temperatures prior to the study commencing. All measurements were performed using high-accuracy methodology traceable to the SI, with small standard measurement uncertainty of individual measurements observed (typically ~ 1%). Total analytical variability for phenylalanine was determined to be 0.84%, and the sample was stable for > 1 year when stored at − 20 °C or 80 °C. More information regarding homogeneity and stability assessment can be found in supplementary information section S2.

### Traceable value assignment of phenylalanine in the study sample

Value assignment was performed using double exact matching isotope dilution gas chromatography with tandem mass spectrometry. The assigned value is the mass fraction of phenylalanine determined using gravimetric preparation and is considered traceable to the SI through use of pure certified reference material for instrument calibration (National Measurement Institute of Japan CRM 6014-a). The quoted uncertainty is the half-width of the expanded uncertainty interval calculated using a coverage factor, *k*, of 2.0, which gives a level of confidence of approximately 95%. The mass fraction (µg/g) with combined expanded uncertainty was then converted to µmol/L using the density of the plasma. Density was determined gravimetrically to maintain traceability of the converted concentration to the SI base unit, the kilogram. The uncertainty of both the plasma density and the molecular mass was calculated and combined with the uncertainty of the value assignment. Details of the calculation for value assignment and associated uncertainty are included in supplementary information S1, along with details of double exact matching isotope dilution mass spectrometry methodology, sample preparation and GC–MS/MS analysis.

### Study design

Three units of the pooled frozen human plasma material were shipped on dry ice to 89 laboratories throughout Europe and participants confirmed that samples remained frozen on receipt. Participants were asked to report concentration (µmol/L) of the 24 amino acids in the three separate identical sample units, to be analysed on three different days. Laboratories used their routine method of amino acid analysis and reported the concentration of each amino acid individually. Details of the sample preparation method and instrumental analysis technique were requested with results.

The SI traceable value for phenylalanine enabled comparison of results to the true phenylalanine concentration. All other amino acid results were used to evaluate harmonisation of the laboratories results in comparison to a consensus mean, since the SI traceable values were not determined.

### Statistical analysis

The intra-laboratory variation was calculated from the mean and standard deviation of the three results submitted for each amino acid from each laboratory. Inter-laboratory variation was determined using the median as the robust estimate of the average from each laboratory, this was to limit the effect of extreme data points. Bias was determined by calculation of percentage difference to the consensus values.

In the absence of established analytical performance specifications (APS) according to the Milan consensus [18], targets for intra-laboratory imprecision and bias have been derived from intra- and inter-individual variation as reported previously [19]. Desirable targets for inter-laboratory imprecision have been established using the Horwitz function [20]. A summary of the desirable performance specifications is in supplementary Table 3.

Consensus values with expanded uncertainty for each amino acid were calculated from the study results. The robust estimator used was the median and associated standard uncertainty, given by Eq. 1 (standard uncertainty for median).

1 $$u= \sqrt{\frac{\pi }{2n}}{MAD}_{E}$$

Where ${MAD}_{E}$ is the median absolute deviation of the individual estimates, scaled to be an equivalent estimator for the population standard deviation, and *n* is the number of replicate values (institutes). Degrees of freedom were sufficiently high for a coverage factor *k* = 2 to be used for the expanded uncertainty associated with the consensus value to give a 95% confidence interval.

Degrees of equivalence (DoE) are measures of agreement between each laboratory and the median of the laboratory estimates, the consensus value. A negative value indicates a lower number than the consensus and a positive indicates a higher number; the closer the DoE is to zero, the closer to the consensus value. The DoE was divided by the expanded uncertainty associated with the DoE (U(DoE)) to generate a score for each analyte from each laboratory; scores are included in the supplementary information S4. A score with an absolute value below 1 is considered satisfactory results, scores between 1 and 1.5 may require further investigation, and scores > 1.5 indicate that the result is not fit-for-purpose and requires investigation. Degrees of equivalence *di* and their standard uncertainties *u*(*di*) were computed using Eq. 2 (calculation for DoE) and Eq. 3 (calculation for standard uncertainty in DoE).

2 $${d}_{i}= {X}_{i}- \widetilde{X}$$

3 $$u\;\left({d}_{i}\right)=u\left(\widetilde{X}\right)\sqrt{1+ \frac{\pi -4}{2n}}$$

Where ${X}_{i}$ is the estimate for laboratory *i*, *n* is the number of laboratories and $\widetilde{X}$ is the median of the lab estimates. The uncertainty in *d*_*i*_ includes the covariance between individual laboratory values and the overall estimate. DoE were reported back to participants to review individual amino acid performance. Degrees of equivalence for each laboratory and analyte are given in supplemental Table 2.

## Results

### Summary of results

Table 1 summarises the number of results submitted including the number of “not detected” results. Not detected results were included as data points and used for the calculation of consensus values, since they are valid measurements that lie below quantification limits, whereas “not analysed” results were discounted from statistical analysis because they are not valid data points.

**Table 1:** Summary of ERNDIM QAA-2106 results. Consensus values, intra- and inter-laboratory %CV and mean bias %

Analyte	Total results submitted (number of not detected)	Consensus value (µmol/L ± U (*k* = 2))	Average intra-laboratory %CV	Inter-laboratory %CV	Mean bias %
1-Methylhistidine	63 (1)	18 ± 1.5	3.5	-	-
3-Methylhistidine	63 (10)	5.5 ± 1.1	9.1	-	-
Alanine	89	377 ± 5.5	2.4	5.0	5.8
Anserine	47 (40)	0.0	-	-	-
Arginine	89 (1)	59 ± 1.6	7.1	10	9.9
Carnosine	50 (47)	0.0	-	-	-
Citrulline	89 (1)	30 ± 0.8	4.0	11	10
Glutamate	88	105 ± 2.6	3.8	9.3	7.9
Glutamine	89	449 ± 10	2.9	9.1	8.5
Glycine	89	257 ± 3.3	2.6	5.8	5.3
Histidine	89	81 ± 1.8	3.1	8.7	8.3
Isoleucine	89	81 ± 1.6	3.2	7.4	7.1
Leucine	89	149 ± 2.5	2.3	6.9	6.5
Lysine	89	176 ± 3.2	2.3	6.5	6.5
Methionine	89	27 ± 0.7	3.3	8.5	10
Ornithine	89 (1)	109 ± 2.2	3.4	8.3	7.4
Phenylalanine	89	359 ± 5.1	2.0	5.4	4.8
Proline	88 (1)	228 ± 4.4	3.6	7.0	7.1
Serine	89	107 ± 2.0	3.2	7.3	6.5
Taurine	84 (1)	48 ± 0.8	3.6	6.2	8.3
Threonine	89 (1)	147 ± 2.2	2.7	5.4	5.3
Tryptophan	74 (3)	52 ± 3.0	4.2	19	15
Tyrosine	89	66 ± 1.1	2.4	6.3	5.6
Valine	89	234 ± 3.2	2.8	6.0	5.1

**Fig. 1:**
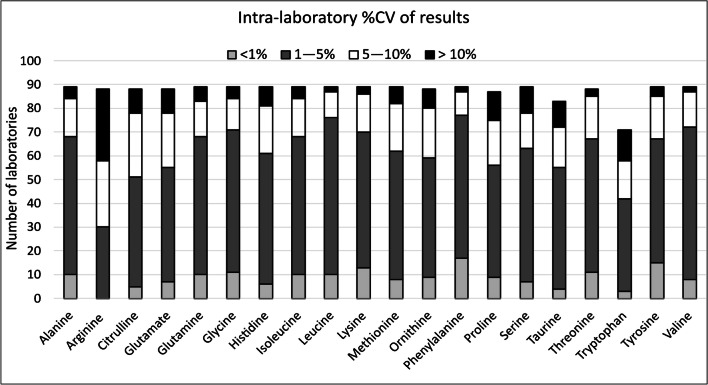
Number of laboratories reporting %CV for each amino acid, grouped into < 1%, 1–5%, 5–10% and > 10%

1-Methylhistidine and 3-methylhistidine both showed a bi-modal distribution, showing the confusion regarding the nomenclature of these analytes. Anserine and carnosine displayed a consensus value of zero as most reported results were “not detected”. Statistical analysis was therefore focussed on the remaining 20 amino acids. Intra-laboratory variation of each analyte was compared against desired performance criteria; 20/20 of the amino acids met the target imprecision. Intra-laboratory variation is illustrated in Fig. 1, to show relative performance of each amino acid.

**Fig. 2:**
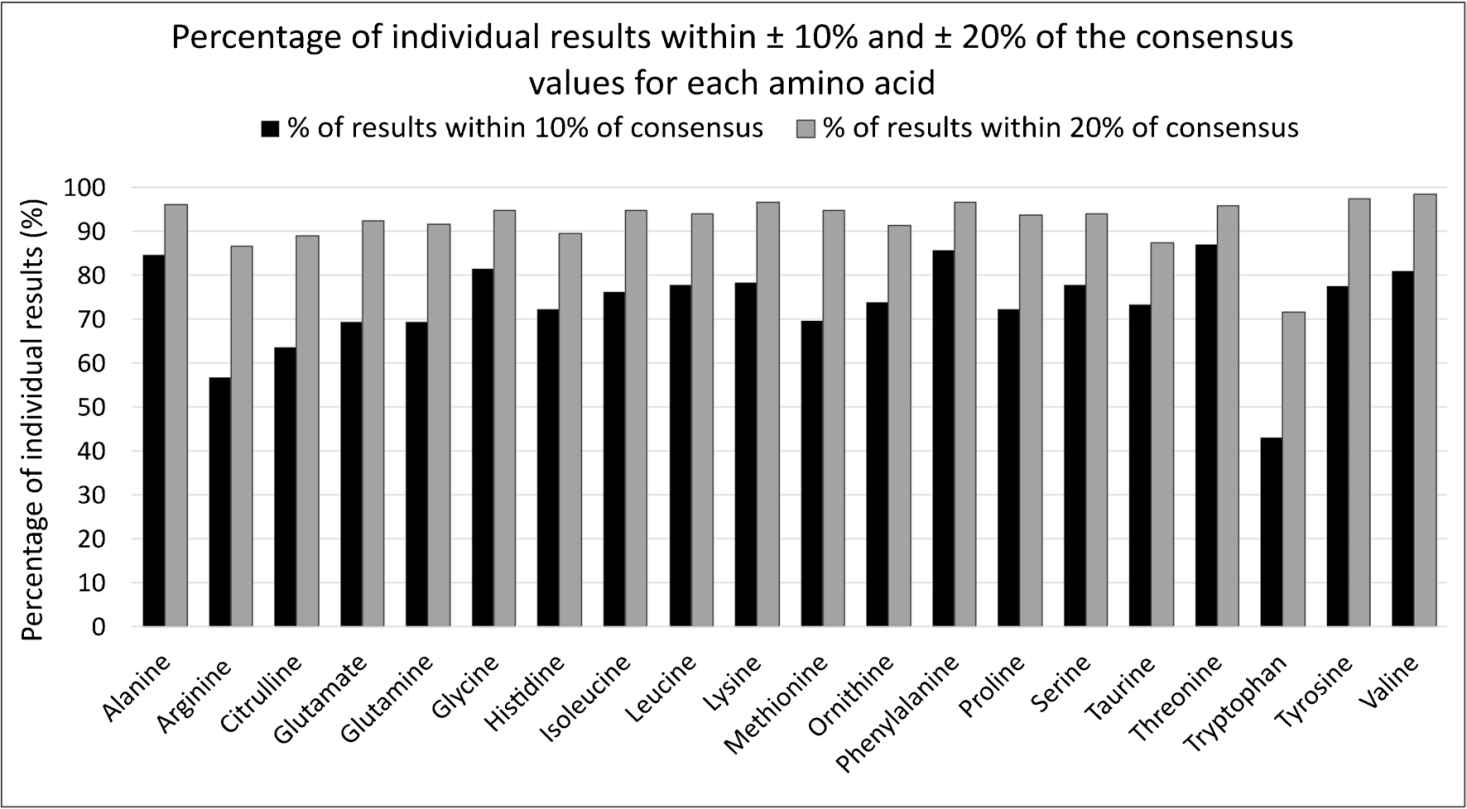
Percentage of individual results within ± 10% and ± 20% of the consensus values for each amino acid

Inter-laboratory variation summarised in Table 1 was compared to the predicted relative standard deviation (PRSD_R_) derived using the Horwitz function; 19/20 of the amino acids met the target %CV. Glutamine was above the target of 8.5%CV, calculated to be 9.1%CV. Results were also considered individually rather than as a laboratory average, as this is how true patient data is reported. To illustrate the performance of the individual amino acids, the percentage of individual results within ± 10% and ± 20% of the consensus values was calculated for each analyte and is depicted in Fig. 2.

**Fig. 3:**
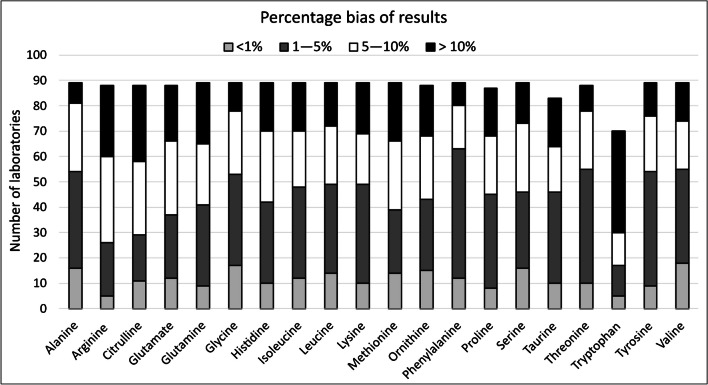
Percentage bias from study consensus value for each amino acid, grouped into < 1%, 1–5%, 5–10% and > 10%

Acceptable bias was demonstrated for 17/20 amino acids. Arginine, glutamine and histidine did not meet the respective targets with biases of 9.9%, 8.5% and 8.3% respectively. Figure 3 illustrates the range of biases determined from the study for all analytes.

### Value assignment of phenylalanine

The mass fraction of phenylalanine in the study sample was determined to be 59.59 ± 1.45 µg/g (95% confidence interval (CI), *k* = 2). This was converted to µmol/L following accurate density determination. The measurement uncertainty of density determination was incorporated into the molar concentration measurement uncertainty, which was calculated to be 368 ± 9.0 µmol/L (95% CI, *k* = 2). Measurement uncertainty was calculated using supplemental Eq. 2.

The assigned concentration range incorporates the target value for dietary monitoring of PKU (360 µmol/L) and the expanded measurement uncertainty at the 95% confidence interval was less than 2.5%. The material used for the study was at a key clinical decision point, with acceptably low measurement uncertainty to satisfy the recommended limit of one-third of desired total measurement uncertainty. The desired total error target for phenylalanine is 18.2% [19].

### Comparison of study results to assigned value

**Fig. 4:**
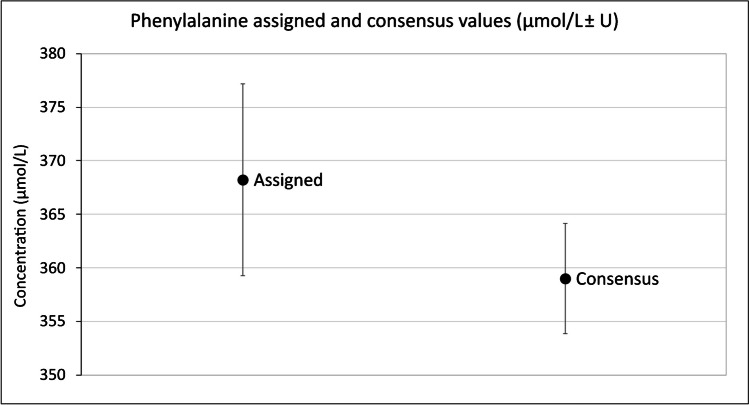
Phenylalanine consensus value of ERNDIM QAA-2016 study and assigned value of plasma sample for phenylalanine. Error bars are ± expanded measurement uncertainty

The consensus value calculated for phenylalanine in the ERNDIM QAA-2016 study was compared to the SI traceable assigned value ± expanded measurement uncertainty. The consensus value expanded measurement uncertainty overlaps with the assigned value expanded measurement uncertainty shown in Fig. 4, indicating that overall, the performance of laboratories is in agreement with the true value. Intra-laboratory variation was 2.0% compared to the desired 4.7%; similarly, inter-laboratory %CV was 5.4% compared to desired 8.7%. Mean bias to the assigned value was 5.1%, only slightly larger than bias to the consensus value (4.8%), both were below the desired bias of 10.4%.

**Fig. 5:**
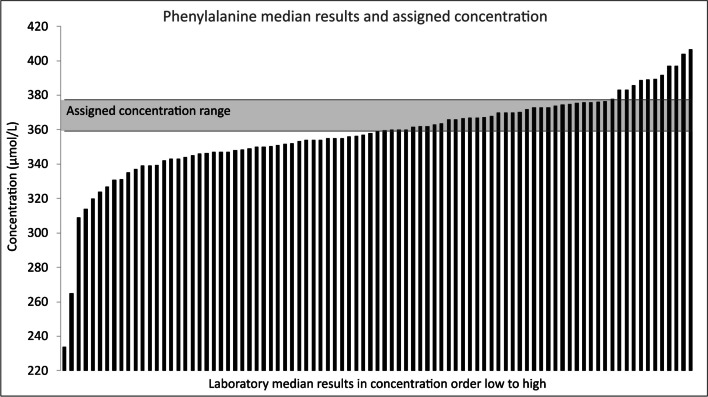
Laboratory median results for phenylalanine in concentration order low to high with assigned concentration range highlighted

However, 15/87 laboratories exceeded the target for imprecision (4.7%) and 8/87 laboratories exceeded the target for bias (10.4%). Additionally, 57 laboratories (64%) did not have their median results within the assigned value range represented in Fig. 5. Significantly, half of all median results (51%) were under-reporting and 14% were over-reporting. With more participants under-reporting than over-reporting, a negative bias of the overall results was identified.

## Discussion

### Amino acid results

Comparing results to desired performance for inter- and intra-laboratory variation, the target inter-laboratory variability was achieved for 19/20 amino acids, and mean intra-laboratory variability was achieved for all 20 amino acids, indicating harmonisation between laboratory results for all analytes. However, when considering individual laboratory results rather than the study averages, imprecision and bias targets were not met for many laboratories across every analyte. Additionally, laboratory measurement bias was identified for 7/88 participants from the calculated DoE’s. Six laboratories calculated DoE’s were all negative (a low result for all reported amino acids) and one showed all positive (a high result for all reported amino acids). Generally, laboratories DoE’s were a scatter for individual amino acids indicating that measurement issues were present for single analytes rather than for the entire analysis method.

Tryptophan was found to have a significant difference in concentration (*p* < 0.05) when using LC–MS/MS in comparison to IEC and HPLC. Comments mentioning co-elution of tryptophan with other amino acids were included in some reports, consistent with the fact that accurate measurement of tryptophan is hampered by protein binding and, for IEC, by poor resolution from histidine and 3-methylhistidine [21]. Aside from tryptophan, statistical analysis showed that the technique utilised does not introduce significant bias in results or affect the precision of results for any of the amino acids.

Direct comparison with previous ERNDIM QTAS distributions is difficult due to the nature of the study sample, a single sample with analytes present at endogenous concentration, and in an alternative matrix. It was not feasible to distribute a typical ERNDIM sample simultaneously for a direct comparison within the scope of the project, irrespective of this, intra-laboratory variation appears improved in comparison to that reported in previous annual reports from the ERNDIM QTAS scheme. This is encouraging, as the sample more closely resembles a patient sample.

A bi-modal distribution was seen for both 1-methylhistidine and 3-methylhistidine, confirming the confusion surrounding the nomenclature of these analytes. The ERNDIM QTAS scheme has included both analytes for the last four years to educate participants about the nomenclature. Despite this, the returns continue to show a bimodal distribution, reflecting the fact that the majority of participants have not yet adopted the correct nomenclature and highlighting the limitation of an EQA scheme, which only reports results relative to the consensus value, not a target value.

The consensus values of anserine and carnosine have been calculated as zero because most results were “not detected”. Seven laboratories reported a concentration for anserine with the mean ranging from 0.30 to 53 µmol/L and three laboratories reported concentration for carnosine with the mean ranging from 1.1 to 6.6 µmol/L. Whilst these are too few results to perform statistical analysis, the range in results indicates issues analysing these compounds amongst the laboratories reporting results.

### Phenylalanine results

The assigned value and consensus value for phenylalanine were in agreement, with the expanded measurement uncertainties of both measures overlapping (Fig. 4). Agreement to the assigned value indicates that all aspects of analysis; recovery, linearity, calibration etc. are performing correctly for analysis of that analyte. However, it cannot be assumed that the other amino acids would have consensus and assigned values in good agreement. Traceable value assignment of other amino acids would be required to establish this.

Typically, a derivation of the ALTM is used as the “correct” result for the QTAS scheme which is analogous to the consensus value calculated here. This value is reported to participants and used to determine the bias that each given laboratory has for each analyte. This study demonstrates that in the absence of an SI traceable material the consensus value could be considered a reasonable estimate for most analytes, but it does not necessarily confer accuracy and may on occasion be misleading. Furthermore, whilst the consensus value agreed with the assigned value, 51% of the laboratories under-reported results whilst a smaller proportion (14%) over-reported results. Patients and clinicians are aiming to avoid adverse neurological effects by controlling plasma phenylalanine concentration to below the target concentration of 360 µmol/L; therefore, the overall negative bias at this concentration has the potential to impact patient care and outcomes. Moreover, a number of laboratories showed a substantial negative bias, the magnitude of which is sufficient to directly influence patient management.

The challenges associated with development and supply of matrix CRMs are well documented: measurand prioritisation, collaboration between NMIs and commutability requirements to name a few. Moreover, most discussion on the topic is concerned with collaboration between reference material producers and in vitro diagnostic manufacturers [22]. When considering laboratory developed tests, which are routinely used for amino acid analysis, the path to traceability is more challenging. However, it is well known that there is a global need for more matrix CRMs to become available to clinical laboratories, as well as greater collaboration between industries, regulatory bodies and standardisation organisations [23]. Currently, a continuous supply of a CRM of this nature for EQA is not feasible at an acceptable cost, however, there are a number of initiatives underway within the metrology community to tackle this challenge [24, 25]. Whilst the provision of an SI traceable material direct to laboratories would help standardise measurement, a cheaper, more practical alternative would be to the provision of such material to EQA providers. This would allow laboratories to regularly compare their performance to a higher order material, be confident that any identified bias was true and then take steps to rectify it. It should be noted that alignment to EQA materials per se is not acceptable practice and is a potential limitation of this approach.

For the purpose of this study, a matrix material with an assigned value that is traceable to the SI was made available. Due to challenges that emerged at the time of the study, homogeneity and stability were monitored to make sure that the material was fit-for-purpose within the duration of the intercomparison. No further commutability study of this material was undertaken, and this is an acknowledged limitation of the study.

## Conclusion

This standalone interlaboratory study has reviewed the performance of amino acids analysis in the majority of European laboratories that provide a routine clinical service. The study has enabled laboratories to compare results using a sample that has been shown to be homogenous, stable and matrix-matched, and therefore more similar to a patient sample than the material distributed by the ERNDIM QTAS scheme. Overall, harmonisation of results across laboratories was deemed acceptable for 17/20 amino acids, yet it is worth noting outliers were present for each amino acid data set.

Comparison of laboratory performance to an SI traceable value for phenylalanine revealed that when all data are considered the consensus value of the study agreed with the assigned value. However, a negative bias was present indicating laboratories are more likely to under-report phenylalanine concentration than over-report. Increased phenylalanine in the blood can lead to adverse neurological outcomes; consequently, underestimation during regular blood testing could have severely detrimental effects to patients. In view of this, the study reported here has highlighted the importance of ensuring an SI traceable material in a suitable matrix is available for EQA to distribute.

## Supplementary Information

Below is the link to the electronic supplementary material.

Supplementary file1 (DOCX 409 KB)

## Abbreviations

IMD: Inherited metabolic disease
EQA: External quality assessment
CRM: Certified reference material
NML: UK National Measurement Laboratory
SI: International system of units
PKU: Phenylketonuria
MSUD: Maple syrup urine disease
HCU: Homocystinuria
IEC: Ion exchange chromatogram
LC–MS/MS: Liquid chromatography tandem mass spectrometry
ERNDIM: European Research Network for the evaluation and improvement of screening, Diagnosis and treatment of Inherited disorders of Metabolism
QTAS: Quantitative amino acids in serum
ALTM: All laboratory trimmed mean
ISO: International standards organisation
HPLC: High performance liquid chromatography
LC-MS: Liquid chromatography mass spectrometry
NMR: Nuclear magnetic resonance
