# Supplementary material for: Development of an SI traceable value assigned amino acid matrix-matched material to underpin European external quality assessment
Source: Anal Bioanal Chem. 2025 Feb 24;417(12):2669–78. doi: 10.1007/s00216-025-05793-4 (PMC12003604; doi:10.1007/s00216-025-05793-4)
Supplement: Supplementary file 1 — Supplementary file1 (DOCX 409 KB) [file 216_2025_5793_MOESM1_ESM.docx]

Supplementary information for: Development of an SI traceable value assigned amino acid matrix-matched material to underpin European external quality assessment

**S1: Double exact matching isotope dilution mass spectrometry (DEM-IDMS) procedure for phenylalanine certification in plasma sample**

Standards

Traceable standard of phenylalanine was obtained from NMIJ, Japan (CRM 6014-a) and stable isotope labelled standard 13C9, 15N-Phenylalanine was purchased from CDN Isotopes. NIST SRM 1950 frozen plasma material was used as quality control (QC) material, and was obtained from Sigma Aldrich (Dorset, UK)

A ~1 mg/mL stock solution was prepared gravimetrically in 100 mM HCl. The stock was diluted gravimetrically to 500 µg/g in 0.1M HCl, and then again gravimetrically to the concentration of the study sample to make a calibration solution. A separate calibration solution was prepared for QC samples at the concentration of the NIST SRM 1950 material as specified on the SRM certificate. Internal standard stock and dilutions were prepared in the same manner as for the calibration solution. The stable isotope internal standard spiking solution was used for both samples and calibration standards, with a separate solution prepared for QC samples and calibration solution.

A standard comparison was performed against a stock prepared by another analyst to ensure accuracy of the stock preparation within the measurement uncertainty of the method. A standard comparison was also performed against freshly prepared stocks prior to 6, 9 and 12 months stability measurements.

Sample preparation for value assignment

For each analytical replicate a 250 µL plasma aliquot was gravimetrically blended with 250 µL stable isotopically labelled analogue solution. After equilibration time of 1 hour the blends were protein precipitated with 1 mL of acetonitrile, vortexed mixed thoroughly and centrifuged for 30 minutes. A 100 µL aliquot of the supernatant was taken to lyophilise by vacuum centrifuge, 100 µL of N-tert-Butyldimethylsilyl-N-methyltrifluoroacetamide (MTBSTFA) with 1% trimethylsilyl (TMS) derivatising agent was added to the dried samples which were then vortex mixed and heated to 85°C for 3 hours for derivatisation of amino acids to the tert-butyldimethylsilyl derivatives.

GC-MS/MS method for value assignment

An Agilent 7010B GC-MS/MS with CTC PAL RSI 120 autosampler was used. As the stationary phase a 30 m × 0.25-mm i.d., 0.25 μm film thickness column was used (Restek). Helium (99.999%) was used as the carrier gas at a constant flow of 1.2 mL/min. The 20-minute oven temperature program was: 130°C hold 3 minutes, ramp 10°C/min to 250°C, ramp 35°C/min to 320°C hold 3 minutes 60 °C. The injector temperature was set at 300 °C and the transfer line was heated to 300 °C. The injection volume was 1 μl in the split mode with a spilt ratio of 20:1. Detection was conducted on MRM transitions of the TDBMS derivatives of leucine and phenylalanine 206>90 (CE10) and 346>317 (CE8) MRMs were used for quantitation respectively. The operation conditions of the mass spectrometer were: electron impact ionization (70 eV) in MRM; emission current, 50 μA; ionization source temperature, 220 °C; electron multiplier voltage, 1500 V; scan width, 0.15; scan time, 0.05 s; and peak width, m/z 0.7 Da. Argon (99.99%) was used as the collision gas at 0.2 Pa.

Quantitation

Each sample was injected five times and quantified using calibration standards that exactly matched the sample concentration, injected immediately before and after the samples.

The calculated amount of phenylalanine in each of the sample extracts was calculated using the DEM-IDMS equation (Supplement equation 1). The DEM-IDMS method allows for a calculation of the uncertainty in the mass fraction phenylalanine in the samples by Supplement equation 2.

*Supplement equation 1*

$$W_{x}=W_{z}.\frac{m_{z}}{m_{yc}}.\frac{m_{y}}{m_{x}}.\frac{R'_{B}}{R'_{BC}}$$

Where:

W_x_ = the mass fraction of phenylalanine in sample

W_z_ = the mass fraction of natural phenylalanine used to prepare the calibration blend

m_z_ = mass of the natural phenylalanine solution added to the calibration blend

m_x_ = mass of the sample used

m_yc_ = mass of the labelled phenylalanine solution added to the calibration blend

m_y_ = mass of the labelled phenylalanine solution added to the sample blend

R’_B_ = measured ratio of the sample blend

R’_BC_= average measured ratio of the calibration blend injected before and after the sample

*Supplement equation 2*

$$u_{c}=w_{x}\sqrt{\left( \frac{u_{Wz}}{w_{z}} \right)^{2}+\left( \frac{{u_{p}}_{R}}{p_{R}} \right)^{2}+\left( \frac{um_{x}}{m_{x}} \right)^{2}+\left( \frac{um_{y}}{m_{y}} \right)^{2}+\left( \frac{um_{z}}{m_{z}} \right)^{2}+\left( \frac{um_{yc}}{m_{yc}} \right)^{2}}$$

Where:

U_Wz_ = the standard uncertainty associated with the mass fraction of the calibration solution

w_z_ = the mass fraction of the calibration solution

um_x_ = the uncertainty associated with the mass of sample used

m_x_ = the mass of sample used

um_y_ = the uncertainty associated with the mass of labelled phenylalanine added to the sample

m_y_ = the mass of labelled phenylalanine added to the sample

u_mz_ = the uncertainty associated with the mass of phenylalanine added to the calibration blend

m_z_ = the mass of phenylalanine added to the calibration blend

um_yc_ = the uncertainty associated with the mass of labelled phenylalanine added to the calibration blend

m_yc_ = the mass of labelled phenylalanine added to the calibration blend

u_PR_ = the standard deviation of the ratios of R’B/R’Bc (n=5)

P_R_ = the mean of R’B/R’Bc (n=5)

**S2: Stability and homogeneity assessment of the plasma sample**

Homogeneity assessment:

Homogeneity and characterisation were performed on 15 units in triplicate analysed across three batches. Within unit, between unit and total analytical variability were assessed using analysis of variance (ANOVA) as this is the most appropriate measure between groups. The results are in Supplement table 1 and shown in the normalised plot Supplement figure 1.

*Supplement table 1: Analytical variability results for homogeneity assessment of phenylalanine*

| **ANOVA Estimate** | **Phenylalanine** |
| --- | --- |
| **Within-unit, CV_wth_:** | 0.39 % |
| **Between-unit, CV_btw_:** | 0.52 % |
| **Total analytical variability, CV:** | 0.84 % |

*Supplemental figure 1: Normalised plot of individual homogeneity results, 15 units in triplicate*.


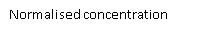

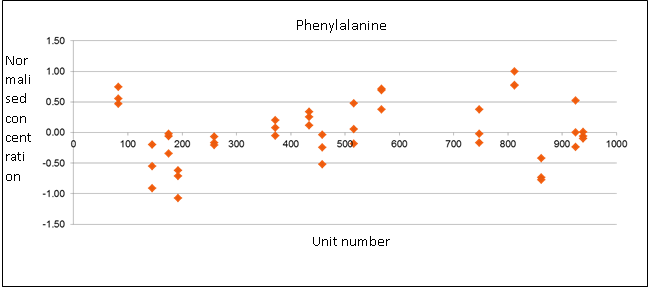


Stability assessment

Short term stability was assessed at 18°C, 4°C -20°C and -80°C at T0, 1 week, 2 weeks and 4 weeks. Long-term stability measurements were performed at 3 months, 6 months, 9 months and 12 months for samples stored at -20°C, and -80°C. The plasma sample was found to be unstable at 4°C for >1 week and unstable at 18°C for >24 hours. The sample was stable within analytical variability of homogeneity assessment at -20°C and -80°C for >1 year.

**S3: List of participating laboratories**

“V.Buzzi” Hospital

AEPEMEC

Alder Hey Children's Hospital

AORMN Presidio Ospedaliero Santa Croce-Fano

AOU MEYER CHILDREN HOSPITAL

Azienda Ospedaliera Universitaria Integrata di Verona

Belfast Health and Social Care Trust

Birmingham Women's and Children's NHS FT

Bruker BioSpin GmbH

Cambridge University Hospitals

CEINGE

Centre Hospitalo-Universitaire de Caen

Centro de Genética Médica - Centro Hospitalar Universitário do Porto

Childrens Clinical University Hospital

Childrens Hospital

CHRU de Nancy

CHU Amiens Picardie

CHU Bordeaux

CHU de Limoges

CHU de Toulouse

CHU GRENOBLE ALPES

Chu Liège

CHU Lille

CHU MONTPELLIER

CHU Nice, FRANCE

CHU TOURS

CHUV

Citta della salute

Cliniques universitaires st Luc

Cochin Hospital APHP

Cruces Hospital

Department der Kinder- und Jugendheilkunde

Dijon University Hospital

EUROFINS BIOMNIS

Evelina London Children's Hospital

Hôpitaux Universitaires de Strasbourg

Hospital Clínic Barcelona

Hospital Clínico Universitario de Santiago

Hospital La Paz

Hospital Sant Joan de Deu

Hospital Universitari Vall d'Hebron

HOSPITAL UNIVERSITARIO LA FE

Hospital Universitario Virgen del Rocío

I.R.C.C.S. Burlo Garofolo

Inselspital Bern

Institute of Child Health

Institute of Mother and Child

Instituto Nacional de Saúde Doutor Ricardo Jorge

Karolinska University Hospital

Labor Berlin GmbH

Labor Dr. Wisplinghoff

Laboratoire national de Santé

Laboratorio Echevarne

Laboratory Biochemical Genetics

Leeds Teaching Hospitals NHS Trust

Lyon University Hospital

MVZ Medizinisches Labor Bremen GmbH

NEOLAB S.A

NHS Lothian

North Bristol NHS Trust

Nottingham University Hospitals NHS Trust

Oslo University Hospital, Rikshospitalet

PMA GmbH

Queen Elizabeth University Hospital, Glasgow

rouen university hospital

Royal Victoria Infirmary

Sahlgrenska University Hospital

Screening-Labor Hannover

Sheffield Children's Hospital

Skane County Council

South West London Pathology

The Cyprus Institute of Neurology & Genetics

UCLH

UMC Utrecht

Univeristy Hospital Wales

university children's hospital of vienna

University Children's Hospital Zurich

University Hospital Leuven

University Hospital of Reims

University Hospital Olomouc

University Hospital Padova

University Hospital Southampton NHS FT

University Hospitals Bristol & Weston NHS Foundation Trust

UZ Brussel

Uz Ghent

Viapath, St Thomas's

Vilnius university Hospital Santaros Clinics

Willink Laboratory

**S4: Supplemental table 2: Individual estimates and degrees of equivalence (DoE)**

**1-methylhistidine**

| **Lab** | **Median** | **MAD** | **u (median)** | **Mean** | **u (mean)** | **n** | **Non-detects** | **DoE** | **u (DoE)** | **Score** |
| --- | --- | --- | --- | --- | --- | --- | --- | --- | --- | --- |
| 1 | 21.6 | 0.7 | 0.5 | 21.7 | 0.4 | 3 | 0 | 3.7 | 4.25 | 0.44 |
| 2 | 19.7 | 1.3 | 1.0 | 19.6 | 0.6 | 3 | 0 | 1.8 | 4.25 | 0.21 |
| 4 | 20.8 | 0.3 | 0.2 | 20.2 | 0.7 | 3 | 0 | 2.9 | 4.25 | 0.34 |
| 6 | 21.0 | 0.0 | 0.0 | 20.7 | 0.3 | 3 | 0 | 3.1 | 4.25 | 0.36 |
| 7 | 4.0 | 0.0 | 0.0 | 4.0 | 0.0 | 3 | 0 | -13.9 | 4.25 | 1.64 |
| 8 | 19.0 | 0.0 | 0.0 | 18.7 | 0.3 | 3 | 0 | 1.1 | 4.25 | 0.13 |
| 10 | 4.3 | 0.2 | 0.2 | 4.3 | 0.1 | 3 | 0 | -13.6 | 4.25 | 1.60 |
| 11 | 18.2 | 0.3 | 0.2 | 18.4 | 0.3 | 3 | 0 | 0.3 | 4.25 | 0.04 |
| 13 | 13.5 | 0.9 | 0.6 | 13.3 | 0.5 | 3 | 0 | -4.4 | 4.25 | 0.52 |
| 15 | 20.9 | 0.1 | 0.1 | 20.9 | 0.1 | 3 | 0 | 3.0 | 4.25 | 0.35 |
| 16 | 13.0 | 0.0 | 0.0 | 14.0 | 1.0 | 3 | 0 | -4.9 | 4.25 | 0.58 |
| 17 | 22.0 | 0.0 | 0.0 | 22.0 | 0.0 | 3 | 0 | 4.1 | 4.25 | 0.48 |
| 19 | 21.0 | 0.0 | 0.0 | 20.6 | 0.4 | 3 | 0 | 3.1 | 4.25 | 0.36 |
| 20 | 7.1 | 0.9 | 0.6 | 9.5 | 2.7 | 3 | 0 | -10.8 | 4.25 | 1.27 |
| 22 | 15.0 | 0.0 | 0.0 | 15.0 | 0.0 | 3 | 0 | -2.9 | 4.25 | 0.34 |
| 23 | 19.6 | 0.9 | 0.6 | 19.6 | 0.4 | 3 | 0 | 1.7 | 4.25 | 0.20 |
| 26 | 223.5 | 60.8 | 44.0 | 241.8 | 40.6 | 3 | 0 | 205.6 | 4.25 | 24.19 |
| 27 | 19.6 | 0.4 | 0.3 | 20.0 | 0.5 | 3 | 0 | 1.7 | 4.25 | 0.20 |
| 28 | 6.0 | 0.0 | 0.0 | 6.3 | 0.3 | 3 | 0 | -11.9 | 4.25 | 1.40 |
| 29 | 17.2 | 0.1 | 0.1 | 17.2 | 0.1 | 3 | 0 | -0.7 | 4.25 | 0.08 |
| 31 | 7.0 | 0.6 | 0.4 | 7.2 | 0.4 | 3 | 0 | -10.9 | 4.25 | 1.28 |
| 32 | 17.9 | 0.0 | 0.0 | 18.2 | 0.3 | 3 | 0 | 0.0 | 4.25 | 0.00 |
| 33 | 18.0 | 4.4 | 3.2 | 17.3 | 2.3 | 3 | 0 | 0.1 | 4.25 | 0.01 |
| 35 | 21.0 | 0.0 | 0.0 | 19.7 | 1.3 | 3 | 0 | 3.1 | 4.25 | 0.36 |
| 36 | 19.3 | 0.6 | 0.4 | 18.3 | 1.3 | 3 | 0 | 1.4 | 4.25 | 0.16 |
| 37 | 20.8 | 0.1 | 0.1 | 20.7 | 0.1 | 3 | 0 | 2.9 | 4.25 | 0.34 |
| 38 | 23.0 | 1.5 | 1.1 | 23.3 | 0.9 | 3 | 0 | 5.1 | 4.25 | 0.60 |
| 39 | 17.3 | 0.1 | 0.1 | 17.0 | 0.4 | 3 | 0 | -0.6 | 4.25 | 0.07 |
| 40 | 4.0 | 0.7 | 0.5 | 4.3 | 0.6 | 3 | 0 | -13.9 | 4.25 | 1.64 |
| 41 | 6.5 | 0.7 | 0.5 | 6.7 | 0.5 | 3 | 0 | -11.4 | 4.25 | 1.34 |
| 42 | 4.9 | 0.0 | 0.0 | 4.8 | 0.1 | 3 | 0 | -13.0 | 4.25 | 1.53 |
| 43 | 16.1 | 0.3 | 0.2 | 16.3 | 0.3 | 3 | 0 | -1.8 | 4.25 | 0.21 |
| 45 | 18.6 | 0.1 | 0.1 | 18.5 | 0.1 | 3 | 0 | 0.7 | 4.25 | 0.08 |
| 48 | 17.9 | 1.8 | 1.3 | 17.6 | 0.9 | 3 | 0 | 0.0 | 4.25 | 0.00 |
| 50 | 22.0 | 3.0 | 2.1 | 21.7 | 1.5 | 3 | 0 | 4.1 | 4.25 | 0.48 |
| 51 | 17.0 | 0.0 | 0.0 | 17.2 | 0.2 | 3 | 0 | -0.9 | 4.25 | 0.11 |
| 52 | 13.0 | 1.5 | 1.1 | 13.3 | 0.9 | 3 | 0 | -4.9 | 4.25 | 0.58 |
| 53 | 5.4 | 0.1 | 0.1 | 5.5 | 0.2 | 3 | 0 | -12.5 | 4.25 | 1.47 |
| 55 | 20.0 | 1.5 | 1.1 | 20.0 | 0.6 | 3 | 0 | 2.1 | 4.25 | 0.25 |
| 56 | 16.2 | 0.9 | 0.6 | 16.2 | 0.3 | 3 | 0 | -1.7 | 4.25 | 0.20 |
| 57 | 4.5 | 0.1 | 0.1 | 4.5 | 0.1 | 3 | 0 | -13.4 | 4.25 | 1.58 |
| **1-methylhistidine** | | | | | | | | | | |
| 59 | 20.0 | 0.0 | 0.0 | 19.7 | 0.3 | 3 | 0 | 2.1 | 4.25 | 0.25 |
| 62 | 19.0 | 0.0 | 0.0 | 18.7 | 0.3 | 3 | 0 | 1.1 | 4.25 | 0.13 |
| 63 | 19.2 | 0.6 | 0.4 | 19.5 | 0.5 | 3 | 0 | 1.3 | 4.25 | 0.15 |
| 64 | 22.0 | 0.0 | 0.0 | 20.3 | 1.7 | 3 | 0 | 4.1 | 4.25 | 0.48 |
| 65 | 4.3 | 2.1 | 1.5 | 4.4 | 0.9 | 3 | 0 | -13.6 | 4.25 | 1.60 |
| 66 | 22.8 | 0.2 | 0.2 | 22.9 | 0.2 | 3 | 0 | 4.9 | 4.25 | 0.58 |
| 67 | 19.7 | 0.1 | 0.1 | 19.1 | 0.7 | 3 | 0 | 1.8 | 4.25 | 0.21 |
| 68 | 22.4 | 5.5 | 4.0 | 23.1 | 2.8 | 3 | 0 | 4.5 | 4.25 | 0.53 |
| 69 | 5.3 | 0.0 | 0.0 | 5.3 | 0.1 | 3 | 0 | -12.6 | 4.25 | 1.48 |
| 70 | 19.0 | 0.0 | 0.0 | 18.7 | 0.3 | 3 | 0 | 1.1 | 4.25 | 0.13 |
| 71 | 20.0 | 0.0 | 0.0 | 19.7 | 0.3 | 3 | 0 | 2.1 | 4.25 | 0.25 |
| 72 | 17.0 | 0.0 | 0.0 | 17.0 | 0.0 | 3 | 0 | -0.9 | 4.25 | 0.11 |
| 73 | 19.0 | 0.0 | 0.0 | 19.3 | 0.3 | 3 | 0 | 1.1 | 4.25 | 0.13 |
| 74 | 5.1 | 0.1 | 0.1 | 5.1 | 0.1 | 3 | 0 | -12.8 | 4.25 | 1.51 |
| 76 | 5.4 | 0.2 | 0.2 | 5.4 | 0.1 | 3 | 0 | -12.5 | 4.25 | 1.47 |
| 77 | 17.0 | 0.0 | 0.0 | 17.0 | 0.0 | 3 | 0 | -0.9 | 4.25 | 0.11 |
| 79 | 12.0 | 1.5 | 1.1 | 12.0 | 0.6 | 3 | 0 | -5.9 | 4.25 | 0.69 |
| 80 | 0.0 | 0.0 | 0.0 | 0.0 | 0.0 | 3 | 1 | -17.9 | 4.25 | 2.11 |
| 81 | 8.7 | 0.6 | 0.4 | 5.9 | 3.0 | 3 | 1 | -9.2 | 4.25 | 1.08 |
| 84 | 15.3 | 0.3 | 0.2 | 15.2 | 0.2 | 3 | 0 | -2.6 | 4.25 | 0.31 |
| 88 | 4.9 | 0.2 | 0.2 | 4.8 | 0.2 | 3 | 0 | -13.0 | 4.25 | 1.53 |
| 90 | 2.9 | 0.9 | 0.6 | 3.0 | 0.5 | 3 | 0 | -15.0 | 4.25 | 1.76 |

**3-methylhistidine**

| **Lab** | **Median** | **MAD** | **u (median)** | **Mean** | **u (mean)** | **n** | **Non-detects** | **DoE** | **u (DoE)** | **Score** |
| --- | --- | --- | --- | --- | --- | --- | --- | --- | --- | --- |
| 1 | 5.4 | 0.3 | 0.2 | 5.3 | 0.2 | 3 | 0 | -0.1 | 3.3 | 0.02 |
| 2 | 0.0 | 0.0 | 0.0 | 0.0 | 0.0 | 3 | 1 | -5.5 | 3.3 | 0.83 |
| 4 | 5.6 | 0.3 | 0.2 | 5.5 | 0.2 | 3 | 0 | 0.1 | 3.3 | 0.02 |
| 6 | 0.0 | 0.0 | 0.0 | 0.0 | 0.0 | 3 | 1 | -5.5 | 3.3 | 0.83 |
| 7 | 14.0 | 1.5 | 1.1 | 13.7 | 0.9 | 3 | 0 | 8.5 | 3.3 | 1.29 |
| 8 | 4.0 | 0.0 | 0.0 | 4.0 | 0.0 | 3 | 0 | -1.5 | 3.3 | 0.23 |
| 9 | 21.6 | 0.1 | 0.1 | 29.8 | 8.2 | 3 | 0 | 16.1 | 3.3 | 2.44 |
| 10 | 17.0 | 2.4 | 1.7 | 16.7 | 1.2 | 3 | 0 | 11.5 | 3.3 | 1.74 |
| 11 | 5.5 | 0.7 | 0.5 | 5.6 | 0.4 | 3 | 0 | 0.0 | 3.3 | 0.00 |
| 13 | 11.5 | 0.3 | 0.2 | 11.6 | 0.2 | 3 | 0 | 6.0 | 3.3 | 0.91 |
| 15 | 4.6 | 0.1 | 0.1 | 4.5 | 0.1 | 3 | 0 | -0.9 | 3.3 | 0.14 |
| 16 | 0.0 | 0.0 | 0.0 | 0.0 | 0.0 | 3 | 1 | -5.5 | 3.3 | 0.83 |
| 17 | 9.0 | 1.5 | 1.1 | 8.7 | 0.9 | 3 | 0 | 3.5 | 3.3 | 0.53 |
| 18 | 6.9 | 0.1 | 0.1 | 6.8 | 0.1 | 3 | 0 | 1.4 | 3.3 | 0.21 |
| 19 | 5.2 | 0.1 | 0.0 | 5.0 | 0.3 | 3 | 0 | -0.3 | 3.3 | 0.05 |
| 20 | 0.0 | 0.0 | 0.0 | 0.0 | 0.0 | 3 | 1 | -5.5 | 3.3 | 0.83 |
| 22 | 10.0 | 1.5 | 1.1 | 10.0 | 0.6 | 3 | 0 | 4.5 | 3.3 | 0.68 |
| 23 | 5.8 | 0.4 | 0.3 | 5.6 | 0.4 | 3 | 0 | 0.3 | 3.3 | 0.05 |
| 26 | 0.0 | 0.0 | 0.0 | 0.0 | 0.0 | 3 | 1 | -5.5 | 3.3 | 0.83 |
| **3-methylhistidine** | | | | | | | | | | |
| 27 | 7.2 | 0.4 | 0.3 | 6.9 | 0.4 | 3 | 0 | 1.7 | 3.3 | 0.26 |
| 28 | 6.0 | 3.0 | 2.1 | 6.0 | 1.2 | 3 | 0 | 0.5 | 3.3 | 0.08 |
| 29 | 3.1 | 0.0 | 0.0 | 3.0 | 0.1 | 3 | 0 | -2.4 | 3.3 | 0.36 |
| 31 | 52.0 | 1.5 | 1.1 | 56.7 | 5.2 | 3 | 0 | 46.5 | 3.3 | 7.05 |
| 32 | 3.1 | 0.0 | 0.0 | 3.0 | 0.1 | 3 | 0 | -2.4 | 3.3 | 0.36 |
| 33 | 4.0 | 0.0 | 0.0 | 4.3 | 0.3 | 3 | 0 | -1.5 | 3.3 | 0.23 |
| 35 | 0.0 | 0.0 | 0.0 | 0.0 | 0.0 | 3 | 1 | -5.5 | 3.3 | 0.83 |
| 36 | 4.9 | 0.1 | 0.1 | 4.5 | 0.4 | 3 | 0 | -0.6 | 3.3 | 0.09 |
| 37 | 5.4 | 0.0 | 0.0 | 5.3 | 0.1 | 3 | 0 | -0.1 | 3.3 | 0.02 |
| 38 | 6.0 | 0.0 | 0.0 | 5.7 | 0.3 | 3 | 0 | 0.5 | 3.3 | 0.08 |
| 40 | 21.0 | 0.0 | 0.0 | 20.2 | 0.8 | 3 | 0 | 15.5 | 3.3 | 2.35 |
| 41 | 14.7 | 0.7 | 0.5 | 14.5 | 0.5 | 3 | 0 | 9.2 | 3.3 | 1.39 |
| 42 | 17.9 | 0.4 | 0.3 | 18.4 | 0.7 | 3 | 0 | 12.4 | 3.3 | 1.88 |
| 43 | 4.6 | 6.8 | 4.9 | 5.7 | 3.7 | 3 | 1 | -0.9 | 3.3 | 0.14 |
| 45 | 5.5 | 0.1 | 0.1 | 5.7 | 0.3 | 3 | 0 | 0.0 | 3.3 | 0.00 |
| 50 | 6.0 | 0.0 | 0.0 | 6.3 | 0.3 | 3 | 0 | 0.5 | 3.3 | 0.08 |
| 51 | 5.0 | 0.0 | 0.0 | 5.1 | 0.1 | 3 | 0 | -0.5 | 3.3 | 0.08 |
| 52 | 9.0 | 0.0 | 0.0 | 8.7 | 0.3 | 3 | 0 | 3.5 | 3.3 | 0.53 |
| 53 | 20.5 | 1.8 | 1.3 | 20.5 | 0.7 | 3 | 0 | 15.0 | 3.3 | 2.27 |
| 55 | 5.0 | 0.0 | 0.0 | 4.7 | 0.3 | 3 | 0 | -0.5 | 3.3 | 0.08 |
| 56 | 4.3 | 0.4 | 0.3 | 4.1 | 0.3 | 3 | 0 | -1.2 | 3.3 | 0.18 |
| 57 | 13.6 | 0.4 | 0.3 | 13.6 | 0.2 | 3 | 0 | 8.1 | 3.3 | 1.23 |
| 59 | 5.0 | 0.0 | 0.0 | 5.3 | 0.3 | 3 | 0 | -0.5 | 3.3 | 0.08 |
| 62 | 6.0 | 1.5 | 1.1 | 6.7 | 1.2 | 3 | 0 | 0.5 | 3.3 | 0.08 |
| 63 | 5.2 | 0.9 | 0.6 | 3.7 | 1.8 | 3 | 1 | -0.3 | 3.3 | 0.05 |
| 64 | 0.0 | 0.0 | 0.0 | 0.0 | 0.0 | 3 | 1 | -5.5 | 3.3 | 0.83 |
| 65 | 23.8 | 0.9 | 0.7 | 24.1 | 0.7 | 3 | 0 | 18.3 | 3.3 | 2.77 |
| 66 | 6.1 | 0.0 | 0.0 | 5.9 | 0.2 | 3 | 0 | 0.6 | 3.3 | 0.09 |
| 67 | 5.6 | 0.3 | 0.2 | 5.7 | 0.2 | 3 | 0 | 0.1 | 3.3 | 0.02 |
| 68 | 4.9 | 0.0 | 0.0 | 5.3 | 0.4 | 3 | 0 | -0.6 | 3.3 | 0.09 |
| 69 | 19.4 | 2.5 | 1.8 | 19.6 | 1.1 | 3 | 0 | 13.9 | 3.3 | 2.11 |
| 70 | 5.0 | 0.0 | 0.0 | 5.0 | 0.0 | 3 | 0 | -0.5 | 3.3 | 0.08 |
| 72 | 5.0 | 0.0 | 0.0 | 4.7 | 0.3 | 3 | 0 | -0.5 | 3.3 | 0.08 |
| 73 | 4.0 | 0.0 | 0.0 | 4.3 | 0.3 | 3 | 0 | -1.5 | 3.3 | 0.23 |
| 74 | 18.2 | 0.9 | 0.6 | 18.8 | 0.9 | 3 | 0 | 12.7 | 3.3 | 1.92 |
| 75 | 9.3 | 0.1 | 0.1 | 9.4 | 0.2 | 3 | 0 | 3.8 | 3.3 | 0.58 |
| 76 | 18.5 | 0.1 | 0.1 | 19.6 | 1.2 | 3 | 0 | 13.0 | 3.3 | 1.97 |
| 77 | 5.0 | 1.5 | 1.1 | 3.7 | 1.9 | 3 | 1 | -0.5 | 3.3 | 0.08 |
| 79 | 0.0 | 0.0 | 0.0 | 0.0 | 0.0 | 3 | 1 | -5.5 | 3.3 | 0.83 |
| 80 | 0.0 | 0.0 | 0.0 | 0.0 | 0.0 | 3 | 1 | -5.5 | 3.3 | 0.83 |
| 81 | 0.0 | 0.0 | 0.0 | 0.0 | 0.0 | 3 | 1 | -5.5 | 3.3 | 0.83 |
| 84 | 2.0 | 0.0 | 0.0 | 2.0 | 0.0 | 3 | 0 | -3.5 | 3.3 | 0.53 |
| 88 | 19.2 | 3.9 | 2.8 | 19.6 | 1.8 | 3 | 0 | 13.7 | 3.3 | 2.08 |
| 90 | 13.3 | 0.1 | 0.1 | 12.8 | 0.5 | 3 | 0 | 7.8 | 3.3 | 1.18 |

**Alanine**

| **Lab** | **Median** | **MAD** | **u (median)** | **Mean** | **u (mean)** | **n** | **Non-detects** | **DoE** | **u (DoE)** | **Score** |
| --- | --- | --- | --- | --- | --- | --- | --- | --- | --- | --- |
| 1 | 387.6 | 3.7 | 2.7 | 385.6 | 3.3 | 3 | 0 | 10.6 | 19.2 | 0.28 |
| 2 | 373.0 | 0.0 | 0.0 | 377.7 | 4.7 | 3 | 0 | -4.0 | 19.2 | 0.10 |
| 3 | 373.0 | 0.0 | 0.0 | 384.7 | 11.7 | 3 | 0 | -4.0 | 19.2 | 0.10 |
| 4 | 34.9 | 1.2 | 0.9 | 34.8 | 0.5 | 3 | 0 | -342.1 | 19.2 | 8.90 |
| 5 | 378.0 | 7.4 | 5.4 | 366.3 | 14.2 | 3 | 0 | 1.0 | 19.2 | 0.03 |
| 6 | 367.0 | 3.0 | 2.1 | 368.0 | 2.1 | 3 | 0 | -10.0 | 19.2 | 0.26 |
| 7 | 361.0 | 25.2 | 18.2 | 362.0 | 10.7 | 3 | 0 | -16.0 | 19.2 | 0.42 |
| 8 | 380.0 | 1.5 | 1.1 | 377.0 | 3.5 | 3 | 0 | 3.0 | 19.2 | 0.08 |
| 9 | 417.1 | 73.1 | 52.9 | 516.1 | 124.5 | 3 | 0 | 40.1 | 19.2 | 1.04 |
| 10 | 376.8 | 0.6 | 0.4 | 381.6 | 4.9 | 3 | 0 | -0.2 | 19.2 | 0.00 |
| 11 | 402.0 | 23.7 | 17.2 | 401.7 | 9.5 | 3 | 0 | 25.0 | 19.2 | 0.65 |
| 12 | 360.3 | 16.8 | 12.1 | 366.1 | 11.9 | 3 | 0 | -16.7 | 19.2 | 0.43 |
| 13 | 407.0 | 8.9 | 6.4 | 408.0 | 4.4 | 3 | 0 | 30.0 | 19.2 | 0.78 |
| 14 | 365.0 | 3.0 | 2.1 | 364.7 | 1.5 | 3 | 0 | -12.0 | 19.2 | 0.31 |
| 15 | 378.3 | 2.4 | 1.7 | 376.8 | 2.3 | 3 | 0 | 1.3 | 19.2 | 0.03 |
| 16 | 381.0 | 5.9 | 4.3 | 380.7 | 2.6 | 3 | 0 | 4.0 | 19.2 | 0.10 |
| 17 | 377.0 | 1.5 | 1.1 | 381.3 | 4.8 | 3 | 0 | 0.0 | 19.2 | 0.00 |
| 18 | 340.0 | 17.8 | 12.9 | 326.7 | 19.6 | 3 | 0 | -37.0 | 19.2 | 0.96 |
| 19 | 388.8 | 16.1 | 11.6 | 389.6 | 6.9 | 3 | 0 | 11.8 | 19.2 | 0.31 |
| 20 | 372.5 | 32.2 | 23.3 | 378.3 | 17.8 | 3 | 0 | -4.5 | 19.2 | 0.12 |
| 21 | 399.0 | 69.7 | 50.4 | 403.3 | 31.0 | 3 | 0 | 22.0 | 19.2 | 0.57 |
| 22 | 427.0 | 3.0 | 2.1 | 426.3 | 1.8 | 3 | 0 | 50.0 | 19.2 | 1.30 |
| 23 | 356.6 | 4.6 | 3.3 | 359.8 | 4.8 | 3 | 0 | -20.4 | 19.2 | 0.53 |
| 24 | 374.0 | 1.5 | 1.1 | 380.7 | 7.2 | 3 | 0 | -3.0 | 19.2 | 0.08 |
| 25 | 392.0 | 1.5 | 1.1 | 394.7 | 3.2 | 3 | 0 | 15.0 | 19.2 | 0.39 |
| 26 | 368.3 | 0.7 | 0.5 | 368.2 | 0.4 | 3 | 0 | -8.7 | 19.2 | 0.23 |
| 27 | 396.4 | 1.9 | 1.4 | 395.1 | 2.0 | 3 | 0 | 19.4 | 19.2 | 0.50 |
| 28 | 377.0 | 3.0 | 2.1 | 373.7 | 4.4 | 3 | 0 | 0.0 | 19.2 | 0.00 |
| 29 | 354.6 | 9.8 | 7.1 | 355.5 | 4.6 | 3 | 0 | -22.4 | 19.2 | 0.58 |
| 30 | 380.1 | 17.2 | 12.4 | 384.0 | 10.3 | 3 | 0 | 3.1 | 19.2 | 0.08 |
| 31 | 348.0 | 13.3 | 9.7 | 337.0 | 15.7 | 3 | 0 | -29.0 | 19.2 | 0.75 |
| 32 | 427.4 | 0.7 | 0.5 | 428.1 | 1.0 | 3 | 0 | 50.4 | 19.2 | 1.31 |
| 33 | 351.0 | 3.0 | 2.1 | 356.0 | 6.0 | 3 | 0 | -26.0 | 19.2 | 0.68 |
| 34 | 402.0 | 105.3 | 76.2 | 401.7 | 41.3 | 3 | 0 | 25.0 | 19.2 | 0.65 |
| 35 | 370.0 | 5.9 | 4.3 | 368.0 | 4.2 | 3 | 0 | -7.0 | 19.2 | 0.18 |
| 36 | 377.5 | 6.3 | 4.5 | 378.4 | 3.2 | 3 | 0 | 0.5 | 19.2 | 0.01 |
| 37 | 373.1 | 3.1 | 2.3 | 375.0 | 3.0 | 3 | 0 | -3.9 | 19.2 | 0.10 |
| 38 | 389.0 | 11.9 | 8.6 | 390.0 | 5.5 | 3 | 0 | 12.0 | 19.2 | 0.31 |
| 39 | 359.0 | 4.4 | 3.2 | 355.7 | 4.9 | 3 | 0 | -18.0 | 19.2 | 0.47 |
| 40 | 398.5 | 9.6 | 7.0 | 400.5 | 5.6 | 3 | 0 | 21.5 | 19.2 | 0.56 |
| 41 | 360.1 | 14.1 | 10.2 | 359.2 | 6.3 | 3 | 0 | -16.9 | 19.2 | 0.44 |
| 42 | 398.8 | 13.9 | 10.1 | 398.7 | 5.5 | 3 | 0 | 21.8 | 19.2 | 0.57 |
| 43 | 344.0 | 8.7 | 6.3 | 344.7 | 4.0 | 3 | 0 | -33.0 | 19.2 | 0.86 |
| 44 | 391.0 | 4.4 | 3.2 | 389.3 | 3.3 | 3 | 0 | 14.0 | 19.2 | 0.36 |
| **Alanine** | | | | | | | | | | |
| 45 | 392.2 | 6.7 | 4.8 | 385.5 | 9.0 | 3 | 0 | 15.2 | 19.2 | 0.40 |
| 46 | 373.0 | 14.8 | 10.7 | 371.3 | 7.3 | 3 | 0 | -4.0 | 19.2 | 0.10 |
| 47 | 374.0 | 8.9 | 6.4 | 380.3 | 9.5 | 3 | 0 | -3.0 | 19.2 | 0.08 |
| 48 | 389.7 | 1.2 | 0.9 | 392.1 | 2.8 | 3 | 0 | 12.7 | 19.2 | 0.33 |
| 49 | 367.0 | 0.0 | 0.0 | 367.0 | 0.0 | 3 | 0 | -10.0 | 19.2 | 0.26 |
| 50 | 358.0 | 7.4 | 5.4 | 355.3 | 5.4 | 3 | 0 | -19.0 | 19.2 | 0.49 |
| 51 | 376.0 | 1.5 | 1.1 | 373.3 | 3.2 | 3 | 0 | -1.0 | 19.2 | 0.03 |
| 52 | 392.0 | 5.9 | 4.3 | 402.7 | 12.7 | 3 | 0 | 15.0 | 19.2 | 0.39 |
| 53 | 407.9 | 3.4 | 2.5 | 411.9 | 5.2 | 3 | 0 | 30.9 | 19.2 | 0.80 |
| 54 | 387.7 | 1.0 | 0.7 | 384.4 | 3.7 | 3 | 0 | 10.7 | 19.2 | 0.28 |
| 55 | 378.0 | 3.0 | 2.1 | 378.0 | 1.2 | 3 | 0 | 1.0 | 19.2 | 0.03 |
| 56 | 364.4 | 10.1 | 7.3 | 362.7 | 5.5 | 3 | 0 | -12.6 | 19.2 | 0.33 |
| 57 | 382.4 | 0.0 | 0.0 | 376.8 | 5.6 | 3 | 0 | 5.4 | 19.2 | 0.14 |
| 58 | 375.0 | 2.8 | 2.1 | 370.8 | 5.2 | 3 | 0 | -2.0 | 19.2 | 0.05 |
| 59 | 374.0 | 0.0 | 0.0 | 368.3 | 5.7 | 3 | 0 | -3.0 | 19.2 | 0.08 |
| 60 | 386.0 | 1.5 | 1.1 | 393.7 | 8.2 | 3 | 0 | 9.0 | 19.2 | 0.23 |
| 61 | 413.2 | 1.4 | 1.0 | 413.0 | 0.7 | 3 | 0 | 36.2 | 19.2 | 0.94 |
| 62 | 371.0 | 3.0 | 2.1 | 377.7 | 7.7 | 3 | 0 | -6.0 | 19.2 | 0.16 |
| 63 | 372.0 | 3.7 | 2.7 | 373.7 | 3.0 | 3 | 0 | -5.0 | 19.2 | 0.13 |
| 64 | 346.0 | 22.2 | 16.1 | 345.7 | 9.0 | 3 | 0 | -31.0 | 19.2 | 0.81 |
| 65 | 426.0 | 18.4 | 13.3 | 416.6 | 16.0 | 3 | 0 | 49.0 | 19.2 | 1.28 |
| 66 | 409.0 | 0.0 | 0.0 | 411.7 | 2.7 | 3 | 0 | 32.0 | 19.2 | 0.83 |
| 67 | 376.0 | 14.8 | 10.7 | 367.3 | 14.0 | 3 | 0 | -1.0 | 19.2 | 0.03 |
| 68 | 398.3 | 6.5 | 4.7 | 407.9 | 11.9 | 3 | 0 | 21.3 | 19.2 | 0.55 |
| 69 | 424.4 | 10.8 | 7.8 | 425.2 | 4.9 | 3 | 0 | 47.4 | 19.2 | 1.23 |
| 70 | 367.0 | 13.3 | 9.7 | 360.0 | 11.8 | 3 | 0 | -10.0 | 19.2 | 0.26 |
| 71 | 336.0 | 11.9 | 8.6 | 342.7 | 10.9 | 3 | 0 | -41.0 | 19.2 | 1.07 |
| 72 | 358.0 | 8.9 | 6.4 | 356.7 | 4.7 | 3 | 0 | -19.0 | 19.2 | 0.49 |
| 73 | 367.0 | 7.4 | 5.4 | 367.0 | 2.9 | 3 | 0 | -10.0 | 19.2 | 0.26 |
| 74 | 412.8 | 7.7 | 5.6 | 405.8 | 9.7 | 3 | 0 | 35.8 | 19.2 | 0.93 |
| 75 | 406.7 | 9.9 | 7.2 | 405.2 | 5.3 | 3 | 0 | 29.7 | 19.2 | 0.77 |
| 76 | 413.5 | 4.9 | 3.5 | 412.7 | 2.7 | 3 | 0 | 36.5 | 19.2 | 0.95 |
| 77 | 363.0 | 10.4 | 7.5 | 371.3 | 12.0 | 3 | 0 | -14.0 | 19.2 | 0.36 |
| 78 | 366.0 | 10.4 | 7.5 | 369.0 | 6.8 | 3 | 0 | -11.0 | 19.2 | 0.29 |
| 79 | 357.0 | 0.0 | 0.0 | 356.3 | 0.7 | 3 | 0 | -20.0 | 19.2 | 0.52 |
| 80 | 195.0 | 1.5 | 1.1 | 197.3 | 2.8 | 3 | 0 | -182.0 | 19.2 | 4.74 |
| 81 | 376.1 | 21.6 | 15.7 | 376.5 | 8.7 | 3 | 0 | -0.9 | 19.2 | 0.02 |
| 83 | 379.8 | 3.0 | 2.1 | 378.6 | 2.3 | 3 | 0 | 2.8 | 19.2 | 0.07 |
| 84 | 399.4 | 18.9 | 13.7 | 394.1 | 12.3 | 3 | 0 | 22.4 | 19.2 | 0.58 |
| 85 | 389.0 | 40.0 | 29.0 | 376.7 | 27.0 | 3 | 0 | 12.0 | 19.2 | 0.31 |
| 86 | 402.0 | 13.3 | 9.7 | 403.7 | 6.7 | 3 | 0 | 25.0 | 19.2 | 0.65 |
| 87 | 373.0 | 11.9 | 8.6 | 372.3 | 5.2 | 3 | 0 | -4.0 | 19.2 | 0.10 |
| 88 | 388.1 | 28.3 | 20.5 | 380.2 | 18.3 | 3 | 0 | 11.1 | 19.2 | 0.29 |
| 89 | 400.0 | 13.3 | 9.7 | 389.0 | 15.7 | 3 | 0 | 23.0 | 19.2 | 0.60 |
| 90 | 393.7 | 7.1 | 5.1 | 390.8 | 5.4 | 3 | 0 | 16.7 | 19.2 | 0.43 |

**Anserine**

| **Lab** | **Median** | **MAD** | **u (median)** | **Mean** | **u (mean)** | **n** | **Non-detects** | **DoE** | **u (DoE)** | **Score** |
| --- | --- | --- | --- | --- | --- | --- | --- | --- | --- | --- |
| 1 | 0.0 | 0.0 | 0.0 | 0.0 | 0.0 | 3 | 1 | 0.0 | 0.0 | N/A |
| 2 | 0.0 | 0.0 | 0.0 | 0.0 | 0.0 | 3 | 1 | 0.0 | 0.0 | N/A |
| 6 | 0.0 | 0.0 | 0.0 | 0.0 | 0.0 | 3 | 1 | 0.0 | 0.0 | N/A |
| 7 | 0.0 | 0.0 | 0.0 | 0.0 | 0.0 | 3 | 1 | 0.0 | 0.0 | N/A |
| 10 | 0.0 | 0.0 | 0.0 | 0.2 | 0.2 | 3 | 1 | 0.0 | 0.0 | N/A |
| 11 | 0.0 | 0.0 | 0.0 | 0.0 | 0.0 | 3 | 1 | 0.0 | 0.0 | N/A |
| 15 | 0.0 | 0.0 | 0.0 | 0.0 | 0.0 | 3 | 1 | 0.0 | 0.0 | N/A |
| 17 | 0.0 | 0.0 | 0.0 | 0.0 | 0.0 | 3 | 1 | 0.0 | 0.0 | N/A |
| 19 | 0.0 | 0.0 | 0.0 | 0.0 | 0.0 | 3 | 1 | 0.0 | 0.0 | N/A |
| 27 | 0.0 | 0.0 | 0.0 | 0.0 | 0.0 | 3 | 1 | 0.0 | 0.0 | N/A |
| 28 | 0.0 | 0.0 | 0.0 | 0.0 | 0.0 | 3 | 1 | 0.0 | 0.0 | N/A |
| 29 | 0.0 | 0.0 | 0.0 | 0.0 | 0.0 | 3 | 1 | 0.0 | 0.0 | N/A |
| 31 | 0.3 | 0.1 | 0.1 | 0.3 | 0.1 | 3 | 0 | 0.3 | 0.0 | N/A |
| 32 | 0.0 | 0.0 | 0.0 | 0.0 | 0.0 | 3 | 1 | 0.0 | 0.0 | N/A |
| 33 | 0.0 | 0.0 | 0.0 | 0.0 | 0.0 | 3 | 1 | 0.0 | 0.0 | N/A |
| 35 | 0.0 | 0.0 | 0.0 | 0.0 | 0.0 | 3 | 1 | 0.0 | 0.0 | N/A |
| 36 | 0.0 | 0.0 | 0.0 | 0.0 | 0.0 | 3 | 1 | 0.0 | 0.0 | N/A |
| 37 | 0.0 | 0.0 | 0.0 | 0.0 | 0.0 | 3 | 1 | 0.0 | 0.0 | N/A |
| 38 | 0.0 | 0.0 | 0.0 | 0.0 | 0.0 | 3 | 1 | 0.0 | 0.0 | N/A |
| 40 | 0.0 | 0.0 | 0.0 | 0.0 | 0.0 | 3 | 1 | 0.0 | 0.0 | N/A |
| 43 | 0.0 | 0.0 | 0.0 | 0.0 | 0.0 | 3 | 1 | 0.0 | 0.0 | N/A |
| 45 | 2.3 | 1.6 | 1.2 | 2.3 | 0.6 | 3 | 0 | 2.3 | 0.0 | N/A |
| 48 | 0.0 | 0.0 | 0.0 | 0.0 | 0.0 | 3 | 1 | 0.0 | 0.0 | N/A |
| 50 | 0.0 | 0.0 | 0.0 | 0.0 | 0.0 | 3 | 1 | 0.0 | 0.0 | N/A |
| 52 | 47.0 | 4.4 | 3.2 | 47.3 | 2.0 | 3 | 0 | 47.0 | 0.0 | N/A |
| 54 | 52.2 | 0.3 | 0.2 | 52.8 | 0.7 | 3 | 0 | 52.2 | 0.0 | N/A |
| 55 | 0.0 | 0.0 | 0.0 | 0.0 | 0.0 | 3 | 1 | 0.0 | 0.0 | N/A |
| 56 | 0.0 | 0.0 | 0.0 | 0.0 | 0.0 | 3 | 1 | 0.0 | 0.0 | N/A |
| 57 | 0.0 | 0.0 | 0.0 | 0.0 | 0.0 | 3 | 1 | 0.0 | 0.0 | N/A |
| 59 | 0.0 | 0.0 | 0.0 | 0.0 | 0.0 | 3 | 1 | 0.0 | 0.0 | N/A |
| 62 | 0.0 | 0.0 | 0.0 | 0.0 | 0.0 | 3 | 1 | 0.0 | 0.0 | N/A |
| 64 | 0.0 | 0.0 | 0.0 | 1.3 | 1.3 | 3 | 1 | 0.0 | 0.0 | N/A |
| 65 | 0.0 | 0.0 | 0.0 | 0.0 | 0.0 | 3 | 1 | 0.0 | 0.0 | N/A |
| 66 | 0.0 | 0.0 | 0.0 | 0.0 | 0.0 | 3 | 1 | 0.0 | 0.0 | N/A |
| 67 | 0.0 | 0.0 | 0.0 | 0.0 | 0.0 | 3 | 1 | 0.0 | 0.0 | N/A |
| 68 | 0.0 | 0.0 | 0.0 | 0.0 | 0.0 | 3 | 1 | 0.0 | 0.0 | N/A |
| 69 | 0.0 | 0.0 | 0.0 | 0.0 | 0.0 | 3 | 1 | 0.0 | 0.0 | N/A |
| 70 | 0.0 | 0.0 | 0.0 | 0.0 | 0.0 | 3 | 1 | 0.0 | 0.0 | N/A |
| 72 | 0.0 | 0.0 | 0.0 | 0.0 | 0.0 | 3 | 1 | 0.0 | 0.0 | N/A |
| 73 | 0.0 | 0.0 | 0.0 | 0.0 | 0.0 | 3 | 1 | 0.0 | 0.0 | N/A |
| 74 | 0.0 | 0.0 | 0.0 | 0.0 | 0.0 | 3 | 1 | 0.0 | 0.0 | N/A |
| 79 | 0.0 | 0.0 | 0.0 | 0.0 | 0.0 | 3 | 1 | 0.0 | 0.0 | N/A |
| 80 | 0.0 | 0.0 | 0.0 | 0.0 | 0.0 | 3 | 1 | 0.0 | 0.0 | N/A |
| 81 | 0.0 | 0.0 | 0.0 | 0.0 | 0.0 | 3 | 1 | 0.0 | 0.0 | N/A |
| **Anserine** | | | | | | | | | | |
| 84 | 0.0 | 0.0 | 0.0 | 0.0 | 0.0 | 3 | 1 | 0.0 | 0.0 | N/A |
| 88 | 0.0 | 0.0 | 0.0 | 0.0 | 0.0 | 3 | 1 | 0.0 | 0.0 | N/A |
| 90 | 7.8 | 0.1 | 0.1 | 7.7 | 0.1 | 3 | 0 | 7.8 | 0.0 | N/A |

**Arginine**

| **Lab** | **Median** | **MAD** | **u (median)** | **Mean** | **u (mean)** | **n** | **Non-detects** | **DoE** | **u (DoE)** | **Score** |
| --- | --- | --- | --- | --- | --- | --- | --- | --- | --- | --- |
| 1 | 61.6 | 0.3 | 0.2 | 63.0 | 1.5 | 3 | 0 | 2.6 | 5.5 | 0.24 |
| 2 | 53.8 | 2.5 | 1.8 | 53.3 | 1.4 | 3 | 0 | -5.2 | 5.5 | 0.47 |
| 3 | 61.0 | 4.4 | 3.2 | 59.0 | 3.6 | 3 | 0 | 2.0 | 5.5 | 0.18 |
| 4 | 60.4 | 8.7 | 6.3 | 60.1 | 3.7 | 3 | 0 | 1.4 | 5.5 | 0.13 |
| 5 | 53.0 | 0.0 | 0.0 | 52.3 | 0.7 | 3 | 0 | -6.0 | 5.5 | 0.55 |
| 6 | 62.0 | 3.0 | 2.1 | 60.7 | 2.4 | 3 | 0 | 3.0 | 5.5 | 0.27 |
| 7 | 85.0 | 1.5 | 1.1 | 79.7 | 5.8 | 3 | 0 | 26.0 | 5.5 | 2.36 |
| 8 | 55.0 | 5.9 | 4.3 | 55.3 | 2.6 | 3 | 0 | -4.0 | 5.5 | 0.36 |
| 9 | 60.2 | 13.2 | 9.5 | 74.3 | 18.7 | 3 | 0 | 1.2 | 5.5 | 0.11 |
| 10 | 58.1 | 0.4 | 0.3 | 57.5 | 0.7 | 3 | 0 | -0.9 | 5.5 | 0.08 |
| 11 | 63.1 | 4.7 | 3.4 | 71.4 | 10.0 | 3 | 0 | 4.1 | 5.5 | 0.37 |
| 12 | 52.0 | 1.5 | 1.1 | 51.7 | 0.9 | 3 | 0 | -7.0 | 5.5 | 0.64 |
| 13 | 57.0 | 4.4 | 3.2 | 58.3 | 3.0 | 3 | 0 | -2.0 | 5.5 | 0.18 |
| 14 | 50.0 | 1.5 | 1.1 | 56.0 | 6.5 | 3 | 0 | -9.0 | 5.5 | 0.82 |
| 15 | 58.9 | 4.9 | 3.6 | 59.3 | 2.3 | 3 | 0 | -0.1 | 5.5 | 0.01 |
| 16 | 59.0 | 4.4 | 3.2 | 58.3 | 2.3 | 3 | 0 | 0.0 | 5.5 | 0.00 |
| 17 | 61.0 | 5.9 | 4.3 | 64.3 | 5.5 | 3 | 0 | 2.0 | 5.5 | 0.18 |
| 18 | 68.0 | 2.1 | 1.5 | 64.5 | 4.3 | 3 | 0 | 9.0 | 5.5 | 0.82 |
| 19 | 59.8 | 1.5 | 1.1 | 60.4 | 1.1 | 3 | 0 | 0.8 | 5.5 | 0.07 |
| 20 | 55.9 | 3.3 | 2.4 | 54.4 | 2.7 | 3 | 0 | -3.1 | 5.5 | 0.28 |
| 21 | 70.0 | 4.4 | 3.2 | 71.3 | 3.0 | 3 | 0 | 11.0 | 5.5 | 1.00 |
| 22 | 62.0 | 0.0 | 0.0 | 60.7 | 1.3 | 3 | 0 | 3.0 | 5.5 | 0.27 |
| 23 | 55.1 | 2.2 | 1.6 | 55.1 | 0.9 | 3 | 0 | -3.9 | 5.5 | 0.35 |
| 24 | 54.0 | 1.5 | 1.1 | 54.3 | 0.9 | 3 | 0 | -5.0 | 5.5 | 0.45 |
| 25 | 59.0 | 1.5 | 1.1 | 59.3 | 0.9 | 3 | 0 | 0.0 | 5.5 | 0.00 |
| 26 | 54.9 | 8.8 | 6.4 | 56.1 | 4.5 | 3 | 0 | -4.1 | 5.5 | 0.37 |
| 27 | 56.3 | 4.6 | 3.3 | 56.6 | 2.1 | 3 | 0 | -2.8 | 5.5 | 0.25 |
| 28 | 47.0 | 1.5 | 1.1 | 47.7 | 1.2 | 3 | 0 | -12.0 | 5.5 | 1.09 |
| 29 | 56.0 | 9.6 | 7.0 | 58.1 | 5.7 | 3 | 0 | -3.0 | 5.5 | 0.27 |
| 30 | 65.3 | 6.4 | 4.6 | 66.4 | 3.5 | 3 | 0 | 6.3 | 5.5 | 0.57 |
| 31 | 60.0 | 1.5 | 1.1 | 61.7 | 2.2 | 3 | 0 | 1.0 | 5.5 | 0.09 |
| 32 | 66.0 | 2.5 | 1.8 | 67.7 | 2.6 | 3 | 0 | 7.0 | 5.5 | 0.64 |
| 33 | 68.0 | 8.9 | 6.4 | 67.0 | 4.4 | 3 | 0 | 9.0 | 5.5 | 0.82 |
| 34 | 61.4 | 7.4 | 5.4 | 62.0 | 3.4 | 3 | 0 | 2.4 | 5.5 | 0.22 |
| 35 | 55.0 | 0.0 | 0.0 | 56.0 | 1.0 | 3 | 0 | -4.0 | 5.5 | 0.36 |
| 36 | 53.2 | 0.2 | 0.2 | 57.9 | 4.8 | 3 | 0 | -5.8 | 5.5 | 0.53 |
| 37 | 54.8 | 1.8 | 1.3 | 55.5 | 1.3 | 3 | 0 | -4.2 | 5.5 | 0.38 |
| 38 | 70.0 | 13.3 | 9.7 | 70.7 | 5.8 | 3 | 0 | 11.0 | 5.5 | 1.00 |
| **Arginine** | | | | | | | | | | |
| 39 | 55.4 | 1.2 | 0.9 | 54.8 | 1.1 | 3 | 0 | -3.6 | 5.5 | 0.33 |
| 40 | 56.0 | 3.7 | 2.7 | 55.7 | 1.7 | 3 | 0 | -3.0 | 5.5 | 0.27 |
| 41 | 54.0 | 0.6 | 0.4 | 51.8 | 2.4 | 3 | 0 | -5.0 | 5.5 | 0.45 |
| 42 | 60.9 | 2.4 | 1.7 | 63.7 | 3.7 | 3 | 0 | 1.9 | 5.5 | 0.17 |
| 43 | 34.1 | 11.0 | 7.9 | 25.2 | 12.8 | 3 | 1 | -24.9 | 5.5 | 2.26 |
| 44 | 50.0 | 1.5 | 1.1 | 52.7 | 3.2 | 3 | 0 | -9.0 | 5.5 | 0.82 |
| 45 | 63.7 | 3.9 | 2.8 | 65.2 | 2.9 | 3 | 0 | 4.7 | 5.5 | 0.43 |
| 46 | 62.1 | 0.7 | 0.5 | 62.7 | 0.9 | 3 | 0 | 3.1 | 5.5 | 0.28 |
| 47 | 69.0 | 5.9 | 4.3 | 68.3 | 2.9 | 3 | 0 | 10.0 | 5.5 | 0.91 |
| 48 | 60.6 | 9.0 | 6.5 | 62.3 | 5.1 | 3 | 0 | 1.6 | 5.5 | 0.15 |
| 49 | 65.1 | 5.5 | 4.0 | 69.1 | 5.9 | 3 | 0 | 6.1 | 5.5 | 0.55 |
| 50 | 56.0 | 4.4 | 3.2 | 58.0 | 3.6 | 3 | 0 | -3.0 | 5.5 | 0.27 |
| 51 | 48.0 | 0.0 | 0.0 | 50.3 | 2.3 | 3 | 0 | -11.0 | 5.5 | 1.00 |
| 52 | 54.0 | 4.4 | 3.2 | 56.0 | 3.6 | 3 | 0 | -5.0 | 5.5 | 0.45 |
| 53 | 113.0 | 7.6 | 5.5 | 112.4 | 3.5 | 3 | 0 | 54.0 | 5.5 | 4.91 |
| 54 | 54.5 | 1.6 | 1.1 | 60.3 | 6.3 | 3 | 0 | -4.5 | 5.5 | 0.41 |
| 55 | 61.0 | 3.0 | 2.1 | 60.7 | 1.5 | 3 | 0 | 2.0 | 5.5 | 0.18 |
| 56 | 53.7 | 0.9 | 0.6 | 54.9 | 1.5 | 3 | 0 | -5.3 | 5.5 | 0.48 |
| 57 | 62.8 | 5.8 | 4.2 | 62.5 | 2.5 | 3 | 0 | 3.8 | 5.5 | 0.35 |
| 58 | 69.2 | 3.4 | 2.4 | 69.6 | 1.7 | 3 | 0 | 10.2 | 5.5 | 0.93 |
| 59 | 52.0 | 0.0 | 0.0 | 55.3 | 3.3 | 3 | 0 | -7.0 | 5.5 | 0.64 |
| 60 | 60.0 | 0.0 | 0.0 | 62.0 | 2.0 | 3 | 0 | 1.0 | 5.5 | 0.09 |
| 61 | 0.0 | 0.0 | 0.0 | 0.0 | 0.0 | 3 | 1 | -59.0 | 5.5 | 5.36 |
| 62 | 58.0 | 4.4 | 3.2 | 61.0 | 4.6 | 3 | 0 | -1.0 | 5.5 | 0.09 |
| 63 | 56.4 | 0.4 | 0.3 | 54.5 | 2.0 | 3 | 0 | -2.6 | 5.5 | 0.24 |
| 64 | 63.0 | 5.9 | 4.3 | 60.7 | 4.5 | 3 | 0 | 4.0 | 5.5 | 0.36 |
| 65 | 62.8 | 9.9 | 7.2 | 61.4 | 5.1 | 3 | 0 | 3.8 | 5.5 | 0.35 |
| 66 | 66.8 | 1.4 | 1.0 | 65.9 | 1.5 | 3 | 0 | 7.8 | 5.5 | 0.71 |
| 67 | 57.4 | 12.5 | 9.0 | 56.4 | 5.7 | 3 | 0 | -1.6 | 5.5 | 0.15 |
| 68 | 63.5 | 3.8 | 2.7 | 62.3 | 2.5 | 3 | 0 | 4.5 | 5.5 | 0.41 |
| 69 | 73.2 | 7.5 | 5.4 | 75.0 | 4.5 | 3 | 0 | 14.2 | 5.5 | 1.29 |
| 70 | 56.0 | 1.5 | 1.1 | 57.3 | 1.9 | 3 | 0 | -3.0 | 5.5 | 0.27 |
| 71 | 46.0 | 7.4 | 5.4 | 46.3 | 3.2 | 3 | 0 | -13.0 | 5.5 | 1.18 |
| 72 | 52.0 | 1.5 | 1.1 | 52.3 | 0.9 | 3 | 0 | -7.0 | 5.5 | 0.64 |
| 73 | 53.0 | 0.0 | 0.0 | 52.3 | 0.7 | 3 | 0 | -6.0 | 5.5 | 0.55 |
| 74 | 62.1 | 1.6 | 1.2 | 62.6 | 1.1 | 3 | 0 | 3.1 | 5.5 | 0.28 |
| 75 | 66.8 | 5.5 | 4.0 | 65.2 | 3.6 | 3 | 0 | 7.8 | 5.5 | 0.71 |
| 76 | 61.3 | 1.5 | 1.1 | 59.0 | 2.8 | 3 | 0 | 2.3 | 5.5 | 0.21 |
| 77 | 63.0 | 0.0 | 0.0 | 61.3 | 1.7 | 3 | 0 | 4.0 | 5.5 | 0.36 |
| 78 | 54.0 | 1.5 | 1.1 | 53.3 | 1.2 | 3 | 0 | -5.0 | 5.5 | 0.45 |
| 79 | 50.0 | 3.0 | 2.1 | 50.3 | 1.5 | 3 | 0 | -9.0 | 5.5 | 0.82 |
| 80 | 42.0 | 1.5 | 1.1 | 42.0 | 0.6 | 3 | 0 | -17.0 | 5.5 | 1.55 |
| 81 | 60.3 | 1.6 | 1.2 | 62.0 | 2.2 | 3 | 0 | 1.3 | 5.5 | 0.12 |
| 83 | 62.4 | 5.8 | 4.2 | 63.2 | 3.0 | 3 | 0 | 3.4 | 5.5 | 0.31 |
| 84 | 53.1 | 0.9 | 0.7 | 52.9 | 0.5 | 3 | 0 | -5.9 | 5.5 | 0.54 |
| **Arginine** | | | | | | | | | | |
| 85 | 59.0 | 1.5 | 1.1 | 62.3 | 3.8 | 3 | 0 | 0.0 | 5.5 | 0.00 |
| 86 | 59.0 | 1.5 | 1.1 | 59.0 | 0.6 | 3 | 0 | 0.0 | 5.5 | 0.00 |
| 87 | 58.0 | 1.5 | 1.1 | 58.7 | 1.2 | 3 | 0 | -1.0 | 5.5 | 0.09 |
| 88 | 61.5 | 1.7 | 1.3 | 64.7 | 3.8 | 3 | 0 | 2.5 | 5.5 | 0.23 |
| 89 | 77.0 | 0.0 | 0.0 | 69.7 | 7.3 | 3 | 0 | 18.0 | 5.5 | 1.64 |
| 90 | 54.7 | 0.6 | 0.4 | 55.0 | 0.5 | 3 | 0 | -4.3 | 5.5 | 0.39 |

**Carnosine**

| **Lab** | **Median** | **MAD** | **u (median)** | **Mean** | **u (mean)** | **n** | **Non-detects** | **DoE** | **u (DoE)** | **Score** |
| --- | --- | --- | --- | --- | --- | --- | --- | --- | --- | --- |
| 1 | 0.0 | 0.0 | 0.0 | 0.0 | 0.0 | 3 | 1 | 0.0 | 0.0 | N/A |
| 2 | 0.0 | 0.0 | 0.0 | 0.0 | 0.0 | 3 | 1 | 0.0 | 0.0 | N/A |
| 6 | 0.0 | 0.0 | 0.0 | 0.0 | 0.0 | 3 | 1 | 0.0 | 0.0 | N/A |
| 7 | 0.0 | 0.0 | 0.0 | 0.0 | 0.0 | 3 | 1 | 0.0 | 0.0 | N/A |
| 10 | 0.0 | 0.0 | 0.0 | 0.4 | 0.4 | 3 | 1 | 0.0 | 0.0 | N/A |
| 11 | 0.0 | 0.0 | 0.0 | 1.4 | 1.4 | 3 | 1 | 0.0 | 0.0 | N/A |
| 15 | 0.0 | 0.0 | 0.0 | 0.0 | 0.0 | 3 | 1 | 0.0 | 0.0 | N/A |
| 16 | 0.0 | 0.0 | 0.0 | 0.0 | 0.0 | 3 | 1 | 0.0 | 0.0 | N/A |
| 17 | 0.0 | 0.0 | 0.0 | 0.0 | 0.0 | 3 | 1 | 0.0 | 0.0 | N/A |
| 19 | 0.0 | 0.0 | 0.0 | 0.0 | 0.0 | 3 | 1 | 0.0 | 0.0 | N/A |
| 20 | 0.0 | 0.0 | 0.0 | 0.0 | 0.0 | 3 | 1 | 0.0 | 0.0 | N/A |
| 26 | 0.0 | 0.0 | 0.0 | 0.0 | 0.0 | 3 | 1 | 0.0 | 0.0 | N/A |
| 27 | 0.0 | 0.0 | 0.0 | 0.0 | 0.0 | 3 | 1 | 0.0 | 0.0 | N/A |
| 28 | 0.0 | 0.0 | 0.0 | 0.0 | 0.0 | 3 | 1 | 0.0 | 0.0 | N/A |
| 29 | 0.0 | 0.0 | 0.0 | 0.0 | 0.0 | 3 | 1 | 0.0 | 0.0 | N/A |
| 30 | 0.0 | 0.0 | 0.0 | 0.0 | 0.0 | 3 | 1 | 0.0 | 0.0 | N/A |
| 31 | 4.0 | 0.0 | 0.0 | 3.7 | 0.3 | 3 | 0 | 4.0 | 0.0 | N/A |
| 32 | 0.0 | 0.0 | 0.0 | 0.0 | 0.0 | 3 | 1 | 0.0 | 0.0 | N/A |
| 33 | 0.0 | 0.0 | 0.0 | 0.0 | 0.0 | 3 | 1 | 0.0 | 0.0 | N/A |
| 35 | 0.0 | 0.0 | 0.0 | 0.0 | 0.0 | 3 | 1 | 0.0 | 0.0 | N/A |
| 36 | 0.0 | 0.0 | 0.0 | 0.0 | 0.0 | 3 | 1 | 0.0 | 0.0 | N/A |
| 37 | 0.0 | 0.0 | 0.0 | 0.0 | 0.0 | 3 | 1 | 0.0 | 0.0 | N/A |
| 38 | 0.0 | 0.0 | 0.0 | 0.0 | 0.0 | 3 | 1 | 0.0 | 0.0 | N/A |
| 40 | 0.0 | 0.0 | 0.0 | 0.0 | 0.0 | 3 | 1 | 0.0 | 0.0 | N/A |
| 43 | 0.0 | 0.0 | 0.0 | 0.0 | 0.0 | 3 | 1 | 0.0 | 0.0 | N/A |
| 45 | 6.6 | 0.7 | 0.5 | 6.6 | 0.3 | 3 | 0 | 6.6 | 0.0 | N/A |
| 48 | 0.0 | 0.0 | 0.0 | 0.0 | 0.0 | 3 | 1 | 0.0 | 0.0 | N/A |
| 50 | 0.0 | 0.0 | 0.0 | 0.0 | 0.0 | 3 | 1 | 0.0 | 0.0 | N/A |
| 52 | 0.0 | 0.0 | 0.0 | 0.0 | 0.0 | 3 | 1 | 0.0 | 0.0 | N/A |
| 55 | 0.0 | 0.0 | 0.0 | 0.0 | 0.0 | 3 | 1 | 0.0 | 0.0 | N/A |
| 56 | 0.0 | 0.0 | 0.0 | 0.0 | 0.0 | 3 | 1 | 0.0 | 0.0 | N/A |
| 57 | 0.0 | 0.0 | 0.0 | 0.0 | 0.0 | 3 | 1 | 0.0 | 0.0 | N/A |
| 59 | 0.0 | 0.0 | 0.0 | 0.0 | 0.0 | 3 | 1 | 0.0 | 0.0 | N/A |
| 62 | 0.0 | 0.0 | 0.0 | 0.0 | 0.0 | 3 | 1 | 0.0 | 0.0 | N/A |
| 65 | 0.0 | 0.0 | 0.0 | 0.0 | 0.0 | 3 | 1 | 0.0 | 0.0 | N/A |
| **Carnosine** | | | | | | | | | | |
| 66 | 0.0 | 0.0 | 0.0 | 0.0 | 0.0 | 3 | 1 | 0.0 | 0.0 | N/A |
| 67 | 0.0 | 0.0 | 0.0 | 0.0 | 0.0 | 2 | NA | 0.0 | 0.0 | N/A |
| 68 | 0.0 | 0.0 | 0.0 | 0.0 | 0.0 | 3 | 1 | 0.0 | 0.0 | N/A |
| 69 | 0.0 | 0.0 | 0.0 | 0.0 | 0.0 | 3 | 1 | 0.0 | 0.0 | N/A |
| 70 | 0.0 | 0.0 | 0.0 | 0.0 | 0.0 | 3 | 1 | 0.0 | 0.0 | N/A |
| 72 | 0.0 | 0.0 | 0.0 | 0.0 | 0.0 | 3 | 1 | 0.0 | 0.0 | N/A |
| 73 | 0.0 | 0.0 | 0.0 | 0.0 | 0.0 | 3 | 1 | 0.0 | 0.0 | N/A |
| 74 | 0.0 | 0.0 | 0.0 | 0.0 | 0.0 | 3 | 1 | 0.0 | 0.0 | N/A |
| 79 | 0.0 | 0.0 | 0.0 | 0.0 | 0.0 | 3 | 1 | 0.0 | 0.0 | N/A |
| 80 | 0.0 | 0.0 | 0.0 | 0.0 | 0.0 | 3 | 1 | 0.0 | 0.0 | N/A |
| 81 | 0.0 | 0.0 | 0.0 | 0.0 | 0.0 | 3 | 1 | 0.0 | 0.0 | N/A |
| 84 | 0.0 | 0.0 | 0.0 | 0.0 | 0.0 | 3 | 1 | 0.0 | 0.0 | N/A |
| 85 | 0.0 | 0.0 | 0.0 | 0.0 | 0.0 | 3 | 1 | 0.0 | 0.0 | N/A |
| 88 | 0.0 | 0.0 | 0.0 | 0.0 | 0.0 | 3 | 1 | 0.0 | 0.0 | N/A |
| 90 | 0.0 | 0.0 | 0.0 | 0.0 | 0.0 | 3 | 1 | 0.0 | 0.0 | N/A |

**Citrulline**

| **Lab** | **Median** | **MAD** | **u (median)** | **Mean** | **u (mean)** | **n** | **Non-detects** | **DoE** | **u (DoE)** | **Score** |
| --- | --- | --- | --- | --- | --- | --- | --- | --- | --- | --- |
| 1 | 32.0 | 1.5 | 1.1 | 32.0 | 0.6 | 3 | 0 | 2.0 | 2.7 | 0.36 |
| 2 | 28.1 | 0.3 | 0.2 | 28.4 | 0.4 | 3 | 0 | -1.9 | 2.7 | 0.35 |
| 3 | 23.0 | 0.0 | 0.0 | 23.7 | 0.7 | 3 | 0 | -7.0 | 2.7 | 1.28 |
| 4 | 32.5 | 0.1 | 0.1 | 32.6 | 0.1 | 3 | 0 | 2.5 | 2.7 | 0.46 |
| 5 | 21.0 | 0.0 | 0.0 | 20.7 | 0.3 | 3 | 0 | -9.0 | 2.7 | 1.64 |
| 6 | 28.0 | 0.0 | 0.0 | 27.7 | 0.3 | 3 | 0 | -2.0 | 2.7 | 0.36 |
| 7 | 29.0 | 1.5 | 1.1 | 28.7 | 0.9 | 3 | 0 | -1.0 | 2.7 | 0.18 |
| 8 | 28.0 | 0.0 | 0.0 | 27.7 | 0.3 | 3 | 0 | -2.0 | 2.7 | 0.36 |
| 9 | 31.3 | 1.8 | 1.3 | 38.7 | 8.0 | 3 | 0 | 1.3 | 2.7 | 0.23 |
| 10 | 33.8 | 0.4 | 0.3 | 33.1 | 0.8 | 3 | 0 | 3.8 | 2.7 | 0.68 |
| 11 | 27.0 | 1.3 | 1.0 | 27.8 | 1.3 | 3 | 0 | -3.0 | 2.7 | 0.55 |
| 12 | 34.0 | 0.0 | 0.0 | 32.9 | 1.1 | 3 | 0 | 4.0 | 2.7 | 0.73 |
| 13 | 30.0 | 0.0 | 0.0 | 30.3 | 0.3 | 3 | 0 | 0.0 | 2.7 | 0.00 |
| 14 | 26.0 | 0.0 | 0.0 | 26.7 | 0.7 | 3 | 0 | -4.0 | 2.7 | 0.73 |
| 15 | 27.7 | 0.3 | 0.2 | 27.9 | 0.3 | 3 | 0 | -2.3 | 2.7 | 0.41 |
| 16 | 47.0 | 0.0 | 0.0 | 48.7 | 1.7 | 3 | 0 | 17.0 | 2.7 | 3.10 |
| 17 | 32.0 | 4.4 | 3.2 | 32.0 | 1.7 | 3 | 0 | 2.0 | 2.7 | 0.36 |
| 18 | 32.0 | 1.2 | 0.9 | 31.3 | 1.2 | 3 | 0 | 2.0 | 2.7 | 0.36 |
| 19 | 30.8 | 1.6 | 1.1 | 30.8 | 0.7 | 3 | 0 | 0.8 | 2.7 | 0.14 |
| 20 | 32.5 | 1.2 | 0.9 | 34.4 | 2.3 | 3 | 0 | 2.5 | 2.7 | 0.46 |
| 21 | 26.0 | 1.5 | 1.1 | 25.7 | 0.9 | 3 | 0 | -4.0 | 2.7 | 0.73 |
| 22 | 33.0 | 3.0 | 2.1 | 33.3 | 1.5 | 3 | 0 | 3.0 | 2.7 | 0.55 |
| 23 | 24.6 | 1.0 | 0.8 | 24.8 | 0.6 | 3 | 0 | -5.4 | 2.7 | 0.98 |
| 24 | 24.0 | 1.5 | 1.1 | 24.3 | 0.9 | 3 | 0 | -6.0 | 2.7 | 1.09 |
| 25 | 30.0 | 0.0 | 0.0 | 29.7 | 0.3 | 3 | 0 | 0.0 | 2.7 | 0.00 |
| 26 | 25.7 | 1.4 | 1.0 | 25.3 | 0.9 | 3 | 0 | -4.3 | 2.7 | 0.79 |
| **Citrulline** | | | | | | | | | | |
| 27 | 30.9 | 0.3 | 0.2 | 30.9 | 0.1 | 3 | 0 | 0.9 | 2.7 | 0.16 |
| 28 | 39.0 | 4.4 | 3.2 | 38.7 | 2.0 | 3 | 0 | 9.0 | 2.7 | 1.64 |
| 29 | 26.7 | 0.3 | 0.2 | 27.2 | 0.6 | 3 | 0 | -3.3 | 2.7 | 0.60 |
| 30 | 38.6 | 1.3 | 1.0 | 37.6 | 1.5 | 3 | 0 | 8.6 | 2.7 | 1.57 |
| 31 | 30.0 | 0.0 | 0.0 | 30.7 | 0.7 | 3 | 0 | 0.0 | 2.7 | 0.00 |
| 32 | 31.3 | 0.1 | 0.1 | 31.0 | 0.4 | 3 | 0 | 1.3 | 2.7 | 0.24 |
| 33 | 29.0 | 0.0 | 0.0 | 28.7 | 0.3 | 3 | 0 | -1.0 | 2.7 | 0.18 |
| 34 | 33.3 | 1.6 | 1.2 | 38.0 | 5.3 | 3 | 0 | 3.3 | 2.7 | 0.60 |
| 35 | 28.0 | 0.0 | 0.0 | 28.0 | 0.0 | 3 | 0 | -2.0 | 2.7 | 0.36 |
| 36 | 26.5 | 0.6 | 0.5 | 26.7 | 0.4 | 3 | 0 | -3.5 | 2.7 | 0.64 |
| 37 | 28.6 | 0.3 | 0.2 | 29.7 | 1.2 | 3 | 0 | -1.4 | 2.7 | 0.26 |
| 38 | 30.0 | 1.5 | 1.1 | 30.3 | 0.9 | 3 | 0 | 0.0 | 2.7 | 0.00 |
| 39 | 27.5 | 0.1 | 0.1 | 27.8 | 0.4 | 3 | 0 | -2.5 | 2.7 | 0.46 |
| 40 | 34.0 | 0.7 | 0.5 | 34.2 | 0.4 | 3 | 0 | 4.0 | 2.7 | 0.73 |
| 41 | 32.4 | 1.6 | 1.2 | 32.3 | 0.7 | 3 | 0 | 2.4 | 2.7 | 0.44 |
| 42 | 31.3 | 0.6 | 0.4 | 31.3 | 0.2 | 3 | 0 | 1.3 | 2.7 | 0.24 |
| 43 | 27.7 | 0.3 | 0.2 | 27.3 | 0.5 | 3 | 0 | -2.3 | 2.7 | 0.42 |
| 44 | 29.0 | 1.5 | 1.1 | 28.3 | 1.2 | 3 | 0 | -1.0 | 2.7 | 0.18 |
| 45 | 31.0 | 0.4 | 0.3 | 30.9 | 0.2 | 3 | 0 | 1.0 | 2.7 | 0.18 |
| 46 | 33.8 | 1.5 | 1.1 | 33.4 | 0.9 | 3 | 0 | 3.8 | 2.7 | 0.69 |
| 47 | 32.0 | 0.0 | 0.0 | 30.3 | 1.7 | 3 | 0 | 2.0 | 2.7 | 0.36 |
| 48 | 29.8 | 2.7 | 1.9 | 28.1 | 2.6 | 3 | 0 | -0.2 | 2.7 | 0.04 |
| 49 | 31.3 | 0.1 | 0.1 | 30.5 | 0.8 | 3 | 0 | 1.3 | 2.7 | 0.24 |
| 50 | 28.0 | 1.5 | 1.1 | 27.7 | 0.9 | 3 | 0 | -2.0 | 2.7 | 0.36 |
| 51 | 33.0 | 1.5 | 1.1 | 33.0 | 0.6 | 3 | 0 | 3.0 | 2.7 | 0.55 |
| 52 | 30.0 | 0.0 | 0.0 | 30.7 | 0.7 | 3 | 0 | 0.0 | 2.7 | 0.00 |
| 53 | 45.9 | 6.7 | 4.8 | 47.4 | 4.0 | 3 | 0 | 15.9 | 2.7 | 2.90 |
| 54 | 33.5 | 0.3 | 0.2 | 32.7 | 0.9 | 3 | 0 | 3.5 | 2.7 | 0.64 |
| 55 | 29.0 | 0.0 | 0.0 | 29.3 | 0.3 | 3 | 0 | -1.0 | 2.7 | 0.18 |
| 56 | 27.6 | 0.8 | 0.5 | 27.4 | 0.5 | 3 | 0 | -2.4 | 2.7 | 0.44 |
| 57 | 27.8 | 0.4 | 0.3 | 28.3 | 0.6 | 3 | 0 | -2.2 | 2.7 | 0.40 |
| 58 | 28.3 | 0.6 | 0.4 | 28.2 | 0.3 | 3 | 0 | -1.8 | 2.7 | 0.32 |
| 59 | 30.0 | 0.0 | 0.0 | 29.7 | 0.3 | 3 | 0 | 0.0 | 2.7 | 0.00 |
| 60 | 32.0 | 3.0 | 2.1 | 32.0 | 1.2 | 3 | 0 | 2.0 | 2.7 | 0.36 |
| 61 | 0.0 | 0.0 | 0.0 | 0.0 | 0.0 | 3 | 1 | -30.0 | 2.7 | 5.46 |
| 62 | 25.0 | 0.0 | 0.0 | 26.0 | 1.0 | 3 | 0 | -5.0 | 2.7 | 0.91 |
| 63 | 31.6 | 0.7 | 0.5 | 31.4 | 0.4 | 3 | 0 | 1.6 | 2.7 | 0.29 |
| 64 | 35.0 | 0.0 | 0.0 | 37.0 | 2.0 | 3 | 0 | 5.0 | 2.7 | 0.91 |
| 65 | 50.4 | 2.6 | 1.9 | 47.6 | 3.7 | 3 | 0 | 20.4 | 2.7 | 3.72 |
| 66 | 33.9 | 0.1 | 0.1 | 33.3 | 0.6 | 3 | 0 | 3.9 | 2.7 | 0.71 |
| 67 | 32.3 | 1.6 | 1.2 | 31.8 | 1.1 | 3 | 0 | 2.3 | 2.7 | 0.42 |
| 68 | 33.5 | 1.1 | 0.8 | 32.5 | 1.4 | 3 | 0 | 3.5 | 2.7 | 0.64 |
| 69 | 33.4 | 0.7 | 0.5 | 33.2 | 0.5 | 3 | 0 | 3.4 | 2.7 | 0.62 |
| 70 | 30.0 | 0.0 | 0.0 | 28.3 | 1.7 | 3 | 0 | 0.0 | 2.7 | 0.00 |
| 71 | 28.0 | 1.5 | 1.1 | 27.3 | 1.2 | 3 | 0 | -2.0 | 2.7 | 0.36 |
| **Citrulline** | | | | | | | | | | |
| 72 | 28.0 | 1.5 | 1.1 | 28.3 | 0.9 | 3 | 0 | -2.0 | 2.7 | 0.36 |
| 73 | 29.0 | 0.0 | 0.0 | 29.3 | 0.3 | 3 | 0 | -1.0 | 2.7 | 0.18 |
| 74 | 31.4 | 0.1 | 0.1 | 31.4 | 0.1 | 3 | 0 | 1.4 | 2.7 | 0.26 |
| 75 | 35.5 | 0.1 | 0.1 | 35.1 | 0.5 | 3 | 0 | 5.5 | 2.7 | 1.00 |
| 76 | 33.5 | 1.5 | 1.1 | 33.4 | 0.7 | 3 | 0 | 3.5 | 2.7 | 0.64 |
| 77 | 29.0 | 1.5 | 1.1 | 30.0 | 1.5 | 3 | 0 | -1.0 | 2.7 | 0.18 |
| 78 | 30.0 | 1.5 | 1.1 | 30.7 | 1.2 | 3 | 0 | 0.0 | 2.7 | 0.00 |
| 79 | 26.0 | 0.0 | 0.0 | 26.3 | 0.3 | 3 | 0 | -4.0 | 2.7 | 0.73 |
| 80 | 17.0 | 0.0 | 0.0 | 16.7 | 0.3 | 3 | 0 | -13.0 | 2.7 | 2.37 |
| 81 | 35.0 | 0.7 | 0.5 | 34.6 | 0.7 | 3 | 0 | 5.0 | 2.7 | 0.91 |
| 83 | 28.9 | 0.3 | 0.2 | 28.9 | 0.1 | 3 | 0 | -1.1 | 2.7 | 0.20 |
| 84 | 28.4 | 1.1 | 0.8 | 29.3 | 1.2 | 3 | 0 | -1.6 | 2.7 | 0.29 |
| 85 | 31.0 | 1.5 | 1.1 | 31.0 | 0.6 | 3 | 0 | 1.0 | 2.7 | 0.18 |
| 86 | 31.0 | 0.4 | 0.3 | 31.0 | 0.2 | 3 | 0 | 1.0 | 2.7 | 0.18 |
| 87 | 28.0 | 1.5 | 1.1 | 28.0 | 0.6 | 3 | 0 | -2.0 | 2.7 | 0.36 |
| 88 | 29.8 | 1.0 | 0.7 | 28.3 | 1.8 | 3 | 0 | -0.2 | 2.7 | 0.04 |
| 89 | 30.0 | 4.4 | 3.2 | 30.3 | 2.0 | 3 | 0 | 0.0 | 2.7 | 0.00 |
| 90 | 31.6 | 2.4 | 1.7 | 30.6 | 1.9 | 3 | 0 | 1.6 | 2.7 | 0.29 |

**Glutamate**

| **Lab** | **Median** | **MAD** | **u (median)** | **Mean** | **u (mean)** | **n** | **Non-detects** | **DoE** | **u (DoE)** | **Score** |
| --- | --- | --- | --- | --- | --- | --- | --- | --- | --- | --- |
| 1 | 122.6 | 1.2 | 0.9 | 122.1 | 1.0 | 3 | 0 | 18.1 | 9.0 | 1.01 |
| 2 | 107.0 | 7.4 | 5.4 | 106.7 | 3.2 | 3 | 0 | 2.5 | 9.0 | 0.14 |
| 3 | 98.0 | 0.0 | 0.0 | 103.7 | 5.7 | 3 | 0 | -6.5 | 9.0 | 0.36 |
| 4 | 100.0 | 0.7 | 0.5 | 101.8 | 2.1 | 3 | 0 | -4.5 | 9.0 | 0.25 |
| 5 | 96.0 | 10.4 | 7.5 | 94.3 | 5.5 | 3 | 0 | -8.5 | 9.0 | 0.48 |
| 6 | 105.0 | 3.0 | 2.1 | 102.7 | 3.4 | 3 | 0 | 0.5 | 9.0 | 0.03 |
| 7 | 139.0 | 20.8 | 15.0 | 131.0 | 15.5 | 3 | 0 | 34.5 | 9.0 | 1.92 |
| 8 | 92.0 | 1.5 | 1.1 | 92.3 | 0.9 | 3 | 0 | -12.5 | 9.0 | 0.70 |
| 9 | 100.9 | 14.2 | 10.3 | 119.5 | 23.5 | 3 | 0 | -3.6 | 9.0 | 0.20 |
| 10 | 104.0 | 0.4 | 0.3 | 104.1 | 0.2 | 3 | 0 | -0.5 | 9.0 | 0.03 |
| 11 | 115.0 | 7.4 | 5.4 | 114.3 | 3.5 | 3 | 0 | 10.5 | 9.0 | 0.58 |
| 12 | 108.0 | 1.5 | 1.1 | 107.5 | 1.1 | 3 | 0 | 3.5 | 9.0 | 0.19 |
| 13 | 114.0 | 1.5 | 1.1 | 114.3 | 0.9 | 3 | 0 | 9.5 | 9.0 | 0.53 |
| 14 | 101.0 | 4.4 | 3.2 | 101.3 | 2.0 | 3 | 0 | -3.5 | 9.0 | 0.20 |
| 15 | 104.7 | 0.5 | 0.4 | 104.5 | 0.4 | 3 | 0 | 0.1 | 9.0 | 0.01 |
| 16 | 87.0 | 0.0 | 0.0 | 86.7 | 0.3 | 3 | 0 | -17.5 | 9.0 | 0.98 |
| 17 | 112.0 | 1.5 | 1.1 | 113.7 | 2.2 | 3 | 0 | 7.5 | 9.0 | 0.42 |
| 18 | 90.0 | 1.5 | 1.1 | 92.7 | 3.2 | 3 | 0 | -14.5 | 9.0 | 0.81 |
| 19 | 102.1 | 3.5 | 2.5 | 102.1 | 1.4 | 3 | 0 | -2.5 | 9.0 | 0.14 |
| 20 | 96.9 | 4.9 | 3.5 | 99.3 | 4.2 | 3 | 0 | -7.6 | 9.0 | 0.43 |
| 21 | 101.0 | 0.0 | 0.0 | 99.3 | 1.7 | 3 | 0 | -3.5 | 9.0 | 0.20 |
| 22 | 108.0 | 0.0 | 0.0 | 107.3 | 0.7 | 3 | 0 | 3.5 | 9.0 | 0.19 |
| 23 | 95.4 | 3.1 | 2.3 | 95.0 | 1.6 | 3 | 0 | -9.1 | 9.0 | 0.51 |
| **Glutamate** | | | | | | | | | | |
| 24 | 106.0 | 1.5 | 1.1 | 106.3 | 0.9 | 3 | 0 | 1.5 | 9.0 | 0.08 |
| 25 | 114.0 | 1.5 | 1.1 | 111.0 | 3.5 | 3 | 0 | 9.5 | 9.0 | 0.53 |
| 26 | 121.0 | 8.7 | 6.3 | 116.8 | 7.4 | 3 | 0 | 16.5 | 9.0 | 0.92 |
| 27 | 110.8 | 1.4 | 1.0 | 110.2 | 1.1 | 3 | 0 | 6.3 | 9.0 | 0.35 |
| 28 | 89.0 | 1.5 | 1.1 | 90.7 | 2.2 | 3 | 0 | -15.5 | 9.0 | 0.87 |
| 29 | 99.1 | 4.9 | 3.5 | 103.5 | 6.1 | 3 | 0 | -5.4 | 9.0 | 0.30 |
| 30 | 104.4 | 0.0 | 0.0 | 101.0 | 3.4 | 3 | 0 | -0.1 | 9.0 | 0.01 |
| 31 | 109.0 | 4.4 | 3.2 | 104.0 | 6.6 | 3 | 0 | 4.5 | 9.0 | 0.25 |
| 32 | 126.2 | 0.1 | 0.1 | 125.9 | 0.4 | 3 | 0 | 21.7 | 9.0 | 1.21 |
| 33 | 101.0 | 4.4 | 3.2 | 102.3 | 3.0 | 3 | 0 | -3.5 | 9.0 | 0.20 |
| 34 | 105.0 | 8.9 | 6.4 | 103.8 | 4.5 | 3 | 0 | 0.5 | 9.0 | 0.03 |
| 35 | 107.0 | 0.0 | 0.0 | 105.7 | 1.3 | 3 | 0 | 2.5 | 9.0 | 0.14 |
| 36 | 109.6 | 1.9 | 1.4 | 109.7 | 0.9 | 3 | 0 | 5.0 | 9.0 | 0.28 |
| 37 | 122.9 | 1.7 | 1.2 | 123.1 | 0.8 | 3 | 0 | 18.4 | 9.0 | 1.02 |
| 38 | 128.0 | 1.5 | 1.1 | 127.0 | 1.5 | 3 | 0 | 23.5 | 9.0 | 1.31 |
| 39 | 97.6 | 0.7 | 0.5 | 97.2 | 0.6 | 3 | 0 | -6.9 | 9.0 | 0.39 |
| 40 | 111.0 | 0.0 | 0.0 | 111.2 | 0.2 | 3 | 0 | 6.5 | 9.0 | 0.36 |
| 41 | 90.2 | 6.2 | 4.5 | 92.4 | 4.5 | 3 | 0 | -14.3 | 9.0 | 0.80 |
| 42 | 108.1 | 1.5 | 1.1 | 105.0 | 3.6 | 3 | 0 | 3.6 | 9.0 | 0.20 |
| 43 | 95.1 | 1.3 | 0.9 | 95.6 | 1.0 | 3 | 0 | -9.5 | 9.0 | 0.53 |
| 44 | 107.0 | 3.0 | 2.1 | 106.0 | 2.1 | 3 | 0 | 2.5 | 9.0 | 0.14 |
| 45 | 106.8 | 2.4 | 1.7 | 104.9 | 2.7 | 3 | 0 | 2.3 | 9.0 | 0.13 |
| 46 | 102.5 | 0.4 | 0.3 | 103.1 | 0.7 | 3 | 0 | -2.0 | 9.0 | 0.11 |
| 47 | 106.0 | 0.0 | 0.0 | 109.7 | 3.7 | 3 | 0 | 1.5 | 9.0 | 0.08 |
| 48 | 96.4 | 2.8 | 2.0 | 94.6 | 2.8 | 3 | 0 | -8.1 | 9.0 | 0.45 |
| 49 | 102.0 | 3.0 | 2.1 | 106.3 | 5.4 | 3 | 0 | -2.5 | 9.0 | 0.14 |
| 50 | 105.0 | 1.5 | 1.1 | 104.7 | 0.9 | 3 | 0 | 0.5 | 9.0 | 0.03 |
| 51 | 81.0 | 1.5 | 1.1 | 80.7 | 0.9 | 3 | 0 | -23.5 | 9.0 | 1.31 |
| 52 | 110.0 | 4.4 | 3.2 | 110.3 | 2.0 | 3 | 0 | 5.5 | 9.0 | 0.30 |
| 53 | 118.9 | 1.9 | 1.4 | 119.0 | 0.9 | 3 | 0 | 14.4 | 9.0 | 0.80 |
| 54 | 119.8 | 1.4 | 1.0 | 117.7 | 2.6 | 3 | 0 | 15.3 | 9.0 | 0.85 |
| 55 | 105.0 | 0.0 | 0.0 | 105.3 | 0.3 | 3 | 0 | 0.5 | 9.0 | 0.03 |
| 56 | 95.2 | 2.4 | 1.7 | 95.7 | 1.4 | 3 | 0 | -9.3 | 9.0 | 0.52 |
| 57 | 100.7 | 2.2 | 1.6 | 100.0 | 1.5 | 3 | 0 | -3.8 | 9.0 | 0.21 |
| 58 | 99.2 | 5.8 | 4.2 | 99.1 | 2.3 | 3 | 0 | -5.4 | 9.0 | 0.30 |
| 59 | 105.0 | 3.0 | 2.1 | 104.7 | 1.5 | 3 | 0 | 0.5 | 9.0 | 0.03 |
| 60 | 100.0 | 0.0 | 0.0 | 97.7 | 2.3 | 3 | 0 | -4.5 | 9.0 | 0.25 |
| 61 | 97.6 | 4.9 | 3.5 | 96.9 | 2.5 | 3 | 0 | -6.9 | 9.0 | 0.39 |
| 62 | 95.0 | 3.0 | 2.1 | 95.0 | 1.2 | 3 | 0 | -9.5 | 9.0 | 0.53 |
| 63 | 97.3 | 2.1 | 1.5 | 97.5 | 1.0 | 3 | 0 | -7.2 | 9.0 | 0.40 |
| 64 | 97.0 | 1.5 | 1.1 | 91.7 | 5.8 | 3 | 0 | -7.5 | 9.0 | 0.42 |
| 65 | 116.8 | 5.9 | 4.2 | 116.5 | 2.5 | 3 | 0 | 12.3 | 9.0 | 0.68 |
| 66 | 120.0 | 8.9 | 6.4 | 120.0 | 3.5 | 3 | 0 | 15.5 | 9.0 | 0.86 |
| 67 | 101.0 | 3.0 | 2.1 | 99.1 | 3.0 | 3 | 0 | -3.5 | 9.0 | 0.20 |
| 68 | 118.7 | 9.3 | 6.8 | 121.4 | 6.1 | 3 | 0 | 14.2 | 9.0 | 0.79 |
| **Glutamate** | | | | | | | | | | |
| 69 | 114.2 | 4.8 | 3.5 | 111.2 | 4.7 | 3 | 0 | 9.7 | 9.0 | 0.54 |
| 70 | 101.0 | 4.4 | 3.2 | 98.7 | 3.9 | 3 | 0 | -3.5 | 9.0 | 0.20 |
| 71 | 97.0 | 3.0 | 2.1 | 96.7 | 1.5 | 3 | 0 | -7.5 | 9.0 | 0.42 |
| 72 | 95.0 | 0.0 | 0.0 | 96.3 | 1.3 | 3 | 0 | -9.5 | 9.0 | 0.53 |
| 73 | 98.0 | 3.0 | 2.1 | 98.0 | 1.2 | 3 | 0 | -6.5 | 9.0 | 0.36 |
| 74 | 136.0 | 12.9 | 9.3 | 134.7 | 6.2 | 3 | 0 | 31.5 | 9.0 | 1.75 |
| 75 | 104.9 | 0.4 | 0.3 | 107.5 | 2.8 | 3 | 0 | 0.4 | 9.0 | 0.02 |
| 76 | 105.2 | 0.1 | 0.1 | 104.4 | 0.8 | 3 | 0 | 0.7 | 9.0 | 0.04 |
| 77 | 98.0 | 4.4 | 3.2 | 97.0 | 2.6 | 3 | 0 | -6.5 | 9.0 | 0.36 |
| 79 | 109.0 | 1.5 | 1.1 | 108.7 | 0.9 | 3 | 0 | 4.5 | 9.0 | 0.25 |
| 80 | 66.0 | 3.0 | 2.1 | 65.3 | 1.8 | 3 | 0 | -38.5 | 9.0 | 2.15 |
| 81 | 99.9 | 4.9 | 3.5 | 101.9 | 3.7 | 3 | 0 | -4.6 | 9.0 | 0.26 |
| 83 | 112.9 | 0.3 | 0.2 | 112.6 | 0.4 | 3 | 0 | 8.4 | 9.0 | 0.47 |
| 84 | 95.9 | 0.1 | 0.1 | 98.3 | 2.4 | 3 | 0 | -8.7 | 9.0 | 0.48 |
| 85 | 108.0 | 1.5 | 1.1 | 111.7 | 4.2 | 3 | 0 | 3.5 | 9.0 | 0.19 |
| 86 | 97.6 | 3.6 | 2.6 | 98.9 | 2.6 | 3 | 0 | -6.9 | 9.0 | 0.39 |
| 87 | 102.0 | 1.5 | 1.1 | 101.7 | 0.9 | 3 | 0 | -2.5 | 9.0 | 0.14 |
| 88 | 93.3 | 6.2 | 4.5 | 97.1 | 6.0 | 3 | 0 | -11.3 | 9.0 | 0.63 |
| 89 | 118.0 | 16.3 | 11.8 | 119.0 | 7.2 | 3 | 0 | 13.5 | 9.0 | 0.75 |
| 90 | 116.0 | 11.9 | 8.6 | 112.9 | 7.4 | 3 | 0 | 11.5 | 9.0 | 0.64 |

**Glutamine**

| **Lab** | **Median** | **MAD** | **u (median)** | **Mean** | **u (mean)** | **n** | **Non-detects** | **DoE** | **u (DoE)** | **Score** |
| --- | --- | --- | --- | --- | --- | --- | --- | --- | --- | --- |
| 1 | 457.5 | 13.5 | 9.8 | 457.7 | 5.4 | 3 | 0 | 8.5 | 35.7 | 0.12 |
| 2 | 438.0 | 11.9 | 8.6 | 441.0 | 7.4 | 3 | 0 | -11.0 | 35.7 | 0.15 |
| 3 | 432.0 | 3.0 | 2.1 | 442.7 | 11.7 | 3 | 0 | -17.0 | 35.7 | 0.24 |
| 4 | 420.0 | 20.8 | 15.0 | 413.0 | 14.6 | 3 | 0 | -29.0 | 35.7 | 0.41 |
| 5 | 465.0 | 25.2 | 18.2 | 449.7 | 24.3 | 3 | 0 | 16.0 | 35.7 | 0.22 |
| 6 | 423.0 | 0.0 | 0.0 | 421.0 | 2.0 | 3 | 0 | -26.0 | 35.7 | 0.36 |
| 7 | 432.0 | 37.1 | 26.8 | 421.0 | 24.6 | 3 | 0 | -17.0 | 35.7 | 0.24 |
| 8 | 439.0 | 7.4 | 5.4 | 429.0 | 12.6 | 3 | 0 | -10.0 | 35.7 | 0.14 |
| 9 | 609.8 | 82.7 | 59.9 | 697.6 | 116.8 | 3 | 0 | 160.8 | 35.7 | 2.25 |
| 10 | 448.0 | 89.4 | 64.7 | 443.9 | 38.4 | 3 | 0 | -1.1 | 35.7 | 0.01 |
| 11 | 383.0 | 7.4 | 5.4 | 431.3 | 50.9 | 3 | 0 | -66.0 | 35.7 | 0.92 |
| 12 | 450.0 | 18.8 | 13.6 | 451.1 | 8.3 | 3 | 0 | 1.0 | 35.7 | 0.01 |
| 13 | 484.0 | 8.9 | 6.4 | 484.3 | 3.8 | 3 | 0 | 35.0 | 35.7 | 0.49 |
| 14 | 430.0 | 11.9 | 8.6 | 428.7 | 5.8 | 3 | 0 | -19.0 | 35.7 | 0.27 |
| 15 | 451.6 | 3.7 | 2.7 | 450.1 | 2.9 | 3 | 0 | 2.6 | 35.7 | 0.04 |
| 16 | 611.0 | 22.2 | 16.1 | 608.0 | 11.4 | 3 | 0 | 162.0 | 35.7 | 2.27 |
| 17 | 493.0 | 1.5 | 1.1 | 488.0 | 5.5 | 3 | 0 | 44.0 | 35.7 | 0.62 |
| 18 | 433.0 | 17.8 | 12.9 | 440.0 | 13.5 | 3 | 0 | -16.0 | 35.7 | 0.22 |
| 19 | 495.6 | 19.1 | 13.8 | 499.6 | 11.0 | 3 | 0 | 46.6 | 35.7 | 0.65 |
| 20 | 444.9 | 17.9 | 13.0 | 451.9 | 13.5 | 3 | 0 | -4.1 | 35.7 | 0.06 |
| 21 | 481.0 | 19.3 | 13.9 | 480.3 | 8.1 | 3 | 0 | 32.0 | 35.7 | 0.45 |
| **Glutamine** | | | | | | | | | | |
| 22 | 500.0 | 8.9 | 6.4 | 497.3 | 5.9 | 3 | 0 | 51.0 | 35.7 | 0.71 |
| 23 | 361.1 | 0.4 | 0.3 | 358.0 | 3.2 | 3 | 0 | -87.9 | 35.7 | 1.23 |
| 24 | 449.0 | 11.9 | 8.6 | 453.0 | 8.3 | 3 | 0 | 0.0 | 35.7 | 0.00 |
| 25 | 434.0 | 7.4 | 5.4 | 434.0 | 2.9 | 3 | 0 | -15.0 | 35.7 | 0.21 |
| 26 | 412.1 | 23.4 | 16.9 | 407.1 | 13.6 | 3 | 0 | -36.9 | 35.7 | 0.52 |
| 27 | 462.7 | 0.3 | 0.2 | 465.2 | 2.6 | 3 | 0 | 13.7 | 35.7 | 0.19 |
| 28 | 428.0 | 8.9 | 6.4 | 426.0 | 5.3 | 3 | 0 | -21.0 | 35.7 | 0.29 |
| 29 | 403.7 | 4.3 | 3.1 | 405.0 | 2.9 | 3 | 0 | -45.3 | 35.7 | 0.63 |
| 30 | 466.2 | 12.2 | 8.8 | 477.2 | 15.3 | 3 | 0 | 17.2 | 35.7 | 0.24 |
| 31 | 406.0 | 1.5 | 1.1 | 421.7 | 16.2 | 3 | 0 | -43.0 | 35.7 | 0.60 |
| 32 | 421.0 | 11.6 | 8.4 | 416.9 | 8.3 | 3 | 0 | -28.0 | 35.7 | 0.39 |
| 33 | 448.0 | 3.0 | 2.1 | 451.7 | 4.7 | 3 | 0 | -1.0 | 35.7 | 0.01 |
| 34 | 528.0 | 32.6 | 23.6 | 514.0 | 25.8 | 3 | 0 | 79.0 | 35.7 | 1.11 |
| 35 | 422.0 | 3.0 | 2.1 | 417.3 | 5.7 | 3 | 0 | -27.0 | 35.7 | 0.38 |
| 36 | 460.4 | 9.3 | 6.8 | 460.8 | 3.9 | 3 | 0 | 11.4 | 35.7 | 0.16 |
| 37 | 504.1 | 2.2 | 1.6 | 500.6 | 4.2 | 3 | 0 | 55.1 | 35.7 | 0.77 |
| 38 | 439.0 | 16.3 | 11.8 | 441.7 | 8.8 | 3 | 0 | -10.0 | 35.7 | 0.14 |
| 39 | 390.0 | 0.0 | 0.0 | 396.3 | 6.3 | 3 | 0 | -59.0 | 35.7 | 0.83 |
| 40 | 464.5 | 14.1 | 10.2 | 467.3 | 8.1 | 3 | 0 | 15.5 | 35.7 | 0.22 |
| 41 | 484.2 | 19.6 | 14.2 | 478.9 | 12.5 | 3 | 0 | 35.2 | 35.7 | 0.49 |
| 42 | 487.0 | 45.5 | 32.9 | 479.3 | 24.7 | 3 | 0 | 38.0 | 35.7 | 0.53 |
| 43 | 404.9 | 22.5 | 16.3 | 405.8 | 9.6 | 3 | 0 | -44.1 | 35.7 | 0.62 |
| 44 | 460.0 | 19.3 | 13.9 | 459.3 | 8.1 | 3 | 0 | 11.0 | 35.7 | 0.15 |
| 45 | 441.5 | 1.3 | 1.0 | 438.7 | 3.2 | 3 | 0 | -7.5 | 35.7 | 0.11 |
| 46 | 473.5 | 7.0 | 5.0 | 476.9 | 5.9 | 3 | 0 | 24.5 | 35.7 | 0.34 |
| 47 | 426.0 | 7.4 | 5.4 | 421.7 | 7.0 | 3 | 0 | -23.0 | 35.7 | 0.32 |
| 48 | 432.1 | 9.9 | 7.2 | 430.8 | 5.0 | 3 | 0 | -16.9 | 35.7 | 0.24 |
| 49 | 519.0 | 23.7 | 17.2 | 524.3 | 14.1 | 3 | 0 | 70.0 | 35.7 | 0.98 |
| 50 | 452.0 | 22.2 | 16.1 | 448.0 | 12.3 | 3 | 0 | 3.0 | 35.7 | 0.04 |
| 51 | 475.0 | 0.0 | 0.0 | 476.0 | 1.0 | 3 | 0 | 26.0 | 35.7 | 0.36 |
| 52 | 461.0 | 0.0 | 0.0 | 475.7 | 14.7 | 3 | 0 | 12.0 | 35.7 | 0.17 |
| 53 | 382.9 | 15.0 | 10.8 | 379.5 | 8.9 | 3 | 0 | -66.1 | 35.7 | 0.93 |
| 54 | 484.3 | 4.1 | 2.9 | 489.7 | 6.8 | 3 | 0 | 35.3 | 35.7 | 0.50 |
| 55 | 433.0 | 0.0 | 0.0 | 433.7 | 0.7 | 3 | 0 | -16.0 | 35.7 | 0.22 |
| 56 | 419.5 | 12.6 | 9.2 | 417.5 | 6.7 | 3 | 0 | -29.5 | 35.7 | 0.41 |
| 57 | 401.2 | 1.6 | 1.2 | 398.3 | 3.4 | 3 | 0 | -47.8 | 35.7 | 0.67 |
| 58 | 457.7 | 4.3 | 3.1 | 457.1 | 2.2 | 3 | 0 | 8.7 | 35.7 | 0.12 |
| 59 | 436.0 | 1.5 | 1.1 | 430.3 | 6.2 | 3 | 0 | -13.0 | 35.7 | 0.18 |
| 60 | 484.0 | 3.0 | 2.1 | 486.0 | 3.1 | 3 | 0 | 35.0 | 35.7 | 0.49 |
| 61 | 539.7 | 5.6 | 4.1 | 538.5 | 3.3 | 3 | 0 | 90.7 | 35.7 | 1.27 |
| 62 | 410.0 | 3.0 | 2.1 | 416.0 | 7.0 | 3 | 0 | -39.0 | 35.7 | 0.55 |
| 63 | 474.0 | 6.2 | 4.5 | 473.0 | 3.3 | 3 | 0 | 25.0 | 35.7 | 0.35 |
| 64 | 320.0 | 1.5 | 1.1 | 323.0 | 3.5 | 3 | 0 | -129.0 | 35.7 | 1.81 |
| 65 | 470.5 | 17.2 | 12.4 | 455.7 | 20.9 | 3 | 0 | 21.5 | 35.7 | 0.30 |
| 66 | 513.0 | 10.4 | 7.5 | 507.0 | 9.7 | 3 | 0 | 64.0 | 35.7 | 0.90 |
| **Glutamine** | | | | | | | | | | |
| 67 | 430.0 | 13.3 | 9.7 | 415.0 | 19.7 | 3 | 0 | -19.0 | 35.7 | 0.27 |
| 68 | 500.6 | 7.8 | 5.6 | 515.5 | 17.6 | 3 | 0 | 51.6 | 35.7 | 0.72 |
| 69 | 488.4 | 10.7 | 7.7 | 487.3 | 5.1 | 3 | 0 | 39.4 | 35.7 | 0.55 |
| 70 | 433.0 | 7.4 | 5.4 | 424.0 | 11.6 | 3 | 0 | -16.0 | 35.7 | 0.22 |
| 71 | 434.0 | 7.4 | 5.4 | 443.7 | 12.3 | 3 | 0 | -15.0 | 35.7 | 0.21 |
| 72 | 441.0 | 4.4 | 3.2 | 440.7 | 2.0 | 3 | 0 | -8.0 | 35.7 | 0.11 |
| 73 | 428.0 | 1.5 | 1.1 | 429.7 | 2.2 | 3 | 0 | -21.0 | 35.7 | 0.29 |
| 74 | 413.4 | 12.2 | 8.8 | 412.7 | 5.4 | 3 | 0 | -35.6 | 35.7 | 0.50 |
| 75 | 450.1 | 8.2 | 5.9 | 446.8 | 6.3 | 3 | 0 | 1.1 | 35.7 | 0.02 |
| 76 | 457.9 | 5.3 | 3.9 | 452.5 | 7.3 | 3 | 0 | 8.9 | 35.7 | 0.12 |
| 77 | 463.0 | 23.7 | 17.2 | 465.7 | 11.6 | 3 | 0 | 14.0 | 35.7 | 0.20 |
| 78 | 430.0 | 7.4 | 5.4 | 427.0 | 5.7 | 3 | 0 | -19.0 | 35.7 | 0.27 |
| 79 | 510.0 | 1.5 | 1.1 | 507.7 | 2.8 | 3 | 0 | 61.0 | 35.7 | 0.85 |
| 80 | 287.0 | 1.5 | 1.1 | 291.0 | 4.5 | 3 | 0 | -162.0 | 35.7 | 2.27 |
| 81 | 490.2 | 1.5 | 1.1 | 490.5 | 0.8 | 3 | 0 | 41.2 | 35.7 | 0.58 |
| 83 | 109.7 | 1.5 | 1.1 | 110.0 | 0.9 | 3 | 0 | -339.3 | 35.7 | 4.75 |
| 84 | 525.7 | 17.8 | 12.9 | 518.8 | 13.4 | 3 | 0 | 76.7 | 35.7 | 1.07 |
| 85 | 445.0 | 5.9 | 4.3 | 436.7 | 10.4 | 3 | 0 | -4.0 | 35.7 | 0.06 |
| 86 | 495.0 | 4.4 | 3.2 | 495.7 | 2.3 | 3 | 0 | 46.0 | 35.7 | 0.64 |
| 87 | 427.0 | 14.8 | 10.7 | 428.3 | 7.0 | 3 | 0 | -22.0 | 35.7 | 0.31 |
| 88 | 504.2 | 68.4 | 49.5 | 501.2 | 29.3 | 3 | 0 | 55.2 | 35.7 | 0.77 |
| 89 | 487.0 | 4.4 | 3.2 | 473.7 | 14.9 | 3 | 0 | 38.0 | 35.7 | 0.53 |
| 90 | 496.5 | 36.2 | 26.2 | 465.1 | 44.2 | 3 | 0 | 47.5 | 35.7 | 0.67 |

**Glycine**

| **Lab** | **Median** | **MAD** | **u (median)** | **Mean** | **u (mean)** | **n** | **Non-detects** | **DoE** | **u (DoE)** | **Score** |
| --- | --- | --- | --- | --- | --- | --- | --- | --- | --- | --- |
| 1 | 265.9 | 1.3 | 1.0 | 264.5 | 1.9 | 3 | 0 | 8.9 | 11.7 | 0.38 |
| 2 | 257.0 | 3.0 | 2.1 | 258.7 | 2.7 | 3 | 0 | 0.0 | 11.7 | 0.00 |
| 3 | 242.0 | 1.5 | 1.1 | 247.7 | 6.2 | 3 | 0 | -15.0 | 11.7 | 0.64 |
| 4 | 260.0 | 4.4 | 3.2 | 269.0 | 10.5 | 3 | 0 | 3.0 | 11.7 | 0.13 |
| 5 | 249.0 | 10.4 | 7.5 | 245.3 | 7.4 | 3 | 0 | -8.0 | 11.7 | 0.34 |
| 6 | 252.0 | 1.5 | 1.1 | 252.7 | 1.2 | 3 | 0 | -5.0 | 11.7 | 0.21 |
| 7 | 256.0 | 20.8 | 15.0 | 279.0 | 30.3 | 3 | 0 | -1.0 | 11.7 | 0.04 |
| 8 | 255.0 | 3.0 | 2.1 | 255.0 | 1.2 | 3 | 0 | -2.0 | 11.7 | 0.09 |
| 9 | 304.8 | 31.5 | 22.8 | 356.7 | 62.8 | 3 | 0 | 47.8 | 11.7 | 2.05 |
| 10 | 252.6 | 1.6 | 1.1 | 256.6 | 4.6 | 3 | 0 | -4.4 | 11.7 | 0.19 |
| 11 | 254.0 | 35.6 | 25.7 | 257.3 | 16.8 | 3 | 0 | -3.0 | 11.7 | 0.13 |
| 12 | 252.0 | 5.9 | 4.3 | 254.7 | 4.8 | 3 | 0 | -5.0 | 11.7 | 0.21 |
| 13 | 280.0 | 4.4 | 3.2 | 279.0 | 2.6 | 3 | 0 | 23.0 | 11.7 | 0.99 |
| 14 | 256.0 | 3.0 | 2.1 | 256.7 | 1.8 | 3 | 0 | -1.0 | 11.7 | 0.04 |
| 15 | 256.4 | 1.8 | 1.3 | 255.5 | 1.5 | 3 | 0 | -0.6 | 11.7 | 0.03 |
| 16 | 255.0 | 1.5 | 1.1 | 257.0 | 2.5 | 3 | 0 | -2.0 | 11.7 | 0.09 |
| 17 | 268.0 | 1.5 | 1.1 | 268.7 | 1.2 | 3 | 0 | 11.0 | 11.7 | 0.47 |
| 18 | 271.0 | 4.4 | 3.2 | 271.0 | 1.7 | 3 | 0 | 14.0 | 11.7 | 0.60 |
| **Glycine** | | | | | | | | | | |
| 19 | 269.7 | 10.9 | 7.9 | 269.7 | 4.3 | 3 | 0 | 12.7 | 11.7 | 0.54 |
| 20 | 256.3 | 5.3 | 3.9 | 265.4 | 10.9 | 3 | 0 | -0.7 | 11.7 | 0.03 |
| 21 | 276.0 | 11.9 | 8.6 | 275.0 | 5.5 | 3 | 0 | 19.0 | 11.7 | 0.81 |
| 22 | 284.0 | 0.0 | 0.0 | 284.3 | 0.3 | 3 | 0 | 27.0 | 11.7 | 1.16 |
| 23 | 248.5 | 7.1 | 5.1 | 249.9 | 4.0 | 3 | 0 | -8.5 | 11.7 | 0.36 |
| 24 | 253.0 | 1.5 | 1.1 | 258.0 | 5.5 | 3 | 0 | -4.0 | 11.7 | 0.17 |
| 25 | 268.0 | 1.5 | 1.1 | 269.3 | 1.9 | 3 | 0 | 11.0 | 11.7 | 0.47 |
| 26 | 233.4 | 13.1 | 9.5 | 242.1 | 13.4 | 3 | 0 | -23.7 | 11.7 | 1.01 |
| 27 | 271.9 | 1.3 | 0.9 | 270.9 | 1.4 | 3 | 0 | 14.9 | 11.7 | 0.64 |
| 28 | 242.0 | 7.4 | 5.4 | 241.7 | 3.2 | 3 | 0 | -15.0 | 11.7 | 0.64 |
| 29 | 241.4 | 6.2 | 4.5 | 242.5 | 3.4 | 3 | 0 | -15.6 | 11.7 | 0.67 |
| 30 | 271.8 | 19.9 | 14.4 | 272.5 | 8.3 | 3 | 0 | 14.8 | 11.7 | 0.63 |
| 31 | 284.0 | 1.5 | 1.1 | 287.3 | 3.8 | 3 | 0 | 27.0 | 11.7 | 1.16 |
| 32 | 308.6 | 0.7 | 0.5 | 309.4 | 1.0 | 3 | 0 | 51.6 | 11.7 | 2.21 |
| 33 | 250.0 | 5.9 | 4.3 | 246.7 | 5.5 | 3 | 0 | -7.0 | 11.7 | 0.30 |
| 34 | 284.0 | 26.7 | 19.3 | 307.0 | 32.4 | 3 | 0 | 27.0 | 11.7 | 1.16 |
| 35 | 244.0 | 5.9 | 4.3 | 242.3 | 3.8 | 3 | 0 | -13.0 | 11.7 | 0.56 |
| 36 | 265.0 | 4.0 | 2.9 | 265.5 | 2.0 | 3 | 0 | 8.0 | 11.7 | 0.34 |
| 37 | 248.0 | 6.0 | 4.4 | 249.3 | 3.5 | 3 | 0 | -9.0 | 11.7 | 0.39 |
| 38 | 266.0 | 5.9 | 4.3 | 264.7 | 3.5 | 3 | 0 | 9.0 | 11.7 | 0.39 |
| 39 | 241.0 | 4.4 | 3.2 | 242.0 | 2.6 | 3 | 0 | -16.0 | 11.7 | 0.69 |
| 40 | 274.0 | 6.7 | 4.8 | 275.5 | 4.0 | 3 | 0 | 17.0 | 11.7 | 0.73 |
| 41 | 250.0 | 9.6 | 7.0 | 251.3 | 5.0 | 3 | 0 | -7.0 | 11.7 | 0.30 |
| 42 | 266.0 | 11.0 | 7.9 | 264.4 | 5.7 | 3 | 0 | 9.0 | 11.7 | 0.39 |
| 43 | 238.0 | 6.9 | 5.0 | 238.2 | 2.9 | 3 | 0 | -19.0 | 11.7 | 0.82 |
| 44 | 262.0 | 1.5 | 1.1 | 259.3 | 3.2 | 3 | 0 | 5.0 | 11.7 | 0.21 |
| 45 | 259.5 | 3.3 | 2.4 | 259.3 | 1.4 | 3 | 0 | 2.5 | 11.7 | 0.11 |
| 46 | 258.7 | 0.6 | 0.4 | 262.0 | 3.5 | 3 | 0 | 1.7 | 11.7 | 0.07 |
| 47 | 280.0 | 8.9 | 6.4 | 275.3 | 7.9 | 3 | 0 | 23.0 | 11.7 | 0.99 |
| 48 | 251.5 | 2.2 | 1.6 | 252.8 | 2.1 | 3 | 0 | -5.5 | 11.7 | 0.24 |
| 49 | 294.0 | 10.4 | 7.5 | 286.0 | 11.7 | 3 | 0 | 37.0 | 11.7 | 1.59 |
| 50 | 258.0 | 8.9 | 6.4 | 257.3 | 4.1 | 3 | 0 | 1.0 | 11.7 | 0.04 |
| 51 | 265.0 | 1.5 | 1.1 | 267.0 | 2.5 | 3 | 0 | 8.0 | 11.7 | 0.34 |
| 52 | 265.0 | 1.5 | 1.1 | 271.0 | 6.5 | 3 | 0 | 8.0 | 11.7 | 0.34 |
| 53 | 264.1 | 14.7 | 10.6 | 262.2 | 7.4 | 3 | 0 | 7.1 | 11.7 | 0.30 |
| 54 | 258.4 | 0.4 | 0.3 | 257.9 | 0.7 | 3 | 0 | 1.4 | 11.7 | 0.06 |
| 55 | 256.0 | 0.0 | 0.0 | 256.3 | 0.3 | 3 | 0 | -1.0 | 11.7 | 0.04 |
| 56 | 251.3 | 8.1 | 5.8 | 250.6 | 3.8 | 3 | 0 | -5.7 | 11.7 | 0.24 |
| 57 | 262.1 | 2.4 | 1.7 | 263.5 | 2.2 | 3 | 0 | 5.1 | 11.7 | 0.22 |
| 58 | 250.6 | 1.8 | 1.3 | 252.3 | 2.3 | 3 | 0 | -6.4 | 11.7 | 0.28 |
| 59 | 256.0 | 0.0 | 0.0 | 252.0 | 4.0 | 3 | 0 | -1.0 | 11.7 | 0.04 |
| 60 | 252.0 | 0.0 | 0.0 | 256.3 | 4.3 | 3 | 0 | -5.0 | 11.7 | 0.21 |
| 61 | 188.6 | 0.1 | 0.1 | 188.4 | 0.3 | 3 | 0 | -68.4 | 11.7 | 2.93 |
| 62 | 235.0 | 4.4 | 3.2 | 239.7 | 6.2 | 3 | 0 | -22.0 | 11.7 | 0.94 |
| 63 | 252.7 | 0.7 | 0.5 | 254.1 | 1.7 | 3 | 0 | -4.3 | 11.7 | 0.18 |
| **Glycine** | | | | | | | | | | |
| 64 | 259.0 | 0.0 | 0.0 | 247.0 | 12.0 | 3 | 0 | 2.0 | 11.7 | 0.09 |
| 65 | 305.9 | 3.6 | 2.6 | 302.0 | 5.1 | 3 | 0 | 48.9 | 11.7 | 2.09 |
| 66 | 288.0 | 3.0 | 2.1 | 289.3 | 2.4 | 3 | 0 | 31.0 | 11.7 | 1.33 |
| 67 | 263.0 | 4.4 | 3.2 | 255.0 | 9.5 | 3 | 0 | 6.0 | 11.7 | 0.26 |
| 68 | 277.6 | 5.0 | 3.6 | 284.3 | 8.4 | 3 | 0 | 20.6 | 11.7 | 0.88 |
| 69 | 271.5 | 8.9 | 6.4 | 271.4 | 3.5 | 3 | 0 | 14.5 | 11.7 | 0.62 |
| 70 | 248.0 | 10.4 | 7.5 | 244.3 | 7.4 | 3 | 0 | -9.0 | 11.7 | 0.39 |
| 71 | 253.0 | 5.9 | 4.3 | 254.7 | 3.8 | 3 | 0 | -4.0 | 11.7 | 0.17 |
| 72 | 252.0 | 11.9 | 8.6 | 251.3 | 5.2 | 3 | 0 | -5.0 | 11.7 | 0.21 |
| 73 | 252.0 | 5.9 | 4.3 | 252.0 | 2.3 | 3 | 0 | -5.0 | 11.7 | 0.21 |
| 74 | 237.6 | 6.2 | 4.5 | 239.6 | 4.3 | 3 | 0 | -19.4 | 11.7 | 0.83 |
| 75 | 278.3 | 5.0 | 3.6 | 276.6 | 3.5 | 3 | 0 | 21.3 | 11.7 | 0.91 |
| 76 | 271.7 | 15.1 | 10.9 | 271.2 | 6.3 | 3 | 0 | 14.7 | 11.7 | 0.63 |
| 77 | 242.0 | 7.4 | 5.4 | 249.7 | 10.3 | 3 | 0 | -15.0 | 11.7 | 0.64 |
| 78 | 242.0 | 7.4 | 5.4 | 245.0 | 5.7 | 3 | 0 | -15.0 | 11.7 | 0.64 |
| 79 | 241.0 | 0.0 | 0.0 | 240.3 | 0.7 | 3 | 0 | -16.0 | 11.7 | 0.69 |
| 80 | 158.0 | 4.4 | 3.2 | 160.7 | 4.3 | 3 | 0 | -99.0 | 11.7 | 4.24 |
| 81 | 258.6 | 3.7 | 2.7 | 257.7 | 2.3 | 3 | 0 | 1.6 | 11.7 | 0.07 |
| 83 | 201.1 | 1.8 | 1.3 | 203.0 | 2.5 | 3 | 0 | -55.9 | 11.7 | 2.40 |
| 84 | 256.3 | 9.7 | 7.0 | 255.1 | 4.9 | 3 | 0 | -0.7 | 11.7 | 0.03 |
| 85 | 265.0 | 44.5 | 32.2 | 273.0 | 24.6 | 3 | 0 | 8.0 | 11.7 | 0.34 |
| 86 | 264.0 | 5.2 | 3.8 | 264.8 | 2.8 | 3 | 0 | 7.0 | 11.7 | 0.30 |
| 87 | 256.0 | 4.4 | 3.2 | 256.3 | 2.0 | 3 | 0 | -1.0 | 11.7 | 0.04 |
| 88 | 263.2 | 13.2 | 9.6 | 258.2 | 9.8 | 3 | 0 | 6.2 | 11.7 | 0.26 |
| 89 | 271.0 | 8.9 | 6.4 | 264.0 | 10.1 | 3 | 0 | 14.0 | 11.7 | 0.60 |
| 90 | 279.7 | 9.6 | 7.0 | 275.6 | 7.6 | 3 | 0 | 22.7 | 11.7 | 0.97 |

**Histidine**

| **Lab** | **Median** | **MAD** | **u (median)** | **Mean** | **u (mean)** | **n** | **Non-detects** | **DoE** | **u (DoE)** | **Score** |
| --- | --- | --- | --- | --- | --- | --- | --- | --- | --- | --- |
| 1 | 86.3 | 0.4 | 0.3 | 85.8 | 0.7 | 3 | 0 | 5.7 | 6.2 | 0.46 |
| 2 | 76.8 | 1.3 | 1.0 | 76.3 | 1.0 | 3 | 0 | -3.8 | 6.2 | 0.31 |
| 3 | 76.0 | 4.4 | 3.2 | 75.0 | 2.6 | 3 | 0 | -4.6 | 6.2 | 0.37 |
| 4 | 85.3 | 0.4 | 0.3 | 85.4 | 0.3 | 3 | 0 | 4.7 | 6.2 | 0.38 |
| 5 | 79.0 | 1.5 | 1.1 | 78.0 | 1.5 | 3 | 0 | -1.6 | 6.2 | 0.13 |
| 6 | 77.0 | 0.0 | 0.0 | 77.7 | 0.7 | 3 | 0 | -3.6 | 6.2 | 0.29 |
| 7 | 89.0 | 4.4 | 3.2 | 90.3 | 3.0 | 3 | 0 | 8.4 | 6.2 | 0.68 |
| 8 | 77.0 | 1.5 | 1.1 | 77.3 | 0.9 | 3 | 0 | -3.6 | 6.2 | 0.29 |
| 9 | 84.5 | 62.9 | 45.5 | 88.8 | 28.4 | 3 | 0 | 3.9 | 6.2 | 0.31 |
| 10 | 83.8 | 3.7 | 2.7 | 82.4 | 2.8 | 3 | 0 | 3.2 | 6.2 | 0.26 |
| 11 | 79.7 | 3.4 | 2.5 | 82.3 | 3.8 | 3 | 0 | -0.9 | 6.2 | 0.07 |
| 12 | 69.8 | 5.7 | 4.1 | 70.3 | 2.6 | 3 | 0 | -10.8 | 6.2 | 0.87 |
| 13 | 88.0 | 1.5 | 1.1 | 87.3 | 1.2 | 3 | 0 | 7.4 | 6.2 | 0.60 |
| 14 | 77.0 | 1.5 | 1.1 | 76.7 | 0.9 | 3 | 0 | -3.6 | 6.2 | 0.29 |
| 15 | 80.6 | 0.4 | 0.3 | 80.2 | 0.6 | 3 | 0 | 0.0 | 6.2 | 0.00 |
| **Histidine** | | | | | | | | | | |
| 16 | 82.0 | 1.5 | 1.1 | 81.7 | 0.9 | 3 | 0 | 1.4 | 6.2 | 0.11 |
| 17 | 85.0 | 3.0 | 2.1 | 85.3 | 1.5 | 3 | 0 | 4.4 | 6.2 | 0.36 |
| 18 | 92.0 | 1.5 | 1.1 | 91.0 | 1.5 | 3 | 0 | 11.4 | 6.2 | 0.92 |
| 19 | 91.4 | 3.7 | 2.7 | 90.7 | 2.0 | 3 | 0 | 10.8 | 6.2 | 0.87 |
| 20 | 74.2 | 4.4 | 3.2 | 74.4 | 1.9 | 3 | 0 | -6.4 | 6.2 | 0.52 |
| 21 | 78.0 | 0.0 | 0.0 | 77.7 | 0.3 | 3 | 0 | -2.6 | 6.2 | 0.21 |
| 22 | 78.0 | 0.0 | 0.0 | 78.7 | 0.7 | 3 | 0 | -2.6 | 6.2 | 0.21 |
| 23 | 76.1 | 0.4 | 0.3 | 76.0 | 0.3 | 3 | 0 | -4.5 | 6.2 | 0.36 |
| 24 | 78.0 | 1.5 | 1.1 | 78.7 | 1.2 | 3 | 0 | -2.6 | 6.2 | 0.21 |
| 25 | 81.0 | 3.0 | 2.1 | 81.7 | 1.8 | 3 | 0 | 0.4 | 6.2 | 0.03 |
| 26 | 126.1 | 17.2 | 12.5 | 128.3 | 8.7 | 3 | 0 | 45.5 | 6.2 | 3.69 |
| 27 | 81.1 | 1.5 | 1.1 | 80.6 | 1.1 | 3 | 0 | 0.5 | 6.2 | 0.04 |
| 28 | 41.0 | 1.5 | 1.1 | 41.3 | 0.9 | 3 | 0 | -39.6 | 6.2 | 3.21 |
| 29 | 66.2 | 2.5 | 1.8 | 68.0 | 2.7 | 3 | 0 | -14.4 | 6.2 | 1.17 |
| 30 | 88.9 | 4.4 | 3.2 | 90.5 | 3.3 | 3 | 0 | 8.3 | 6.2 | 0.67 |
| 31 | 88.0 | 4.4 | 3.2 | 92.0 | 5.6 | 3 | 0 | 7.4 | 6.2 | 0.60 |
| 32 | 90.9 | 0.3 | 0.2 | 90.3 | 0.7 | 3 | 0 | 10.3 | 6.2 | 0.83 |
| 33 | 82.0 | 1.5 | 1.1 | 81.3 | 1.2 | 3 | 0 | 1.4 | 6.2 | 0.11 |
| 34 | 99.2 | 10.1 | 7.3 | 98.3 | 4.7 | 3 | 0 | 18.6 | 6.2 | 1.51 |
| 35 | 78.0 | 3.0 | 2.1 | 77.3 | 1.8 | 3 | 0 | -2.6 | 6.2 | 0.21 |
| 36 | 81.4 | 1.6 | 1.1 | 81.3 | 0.7 | 3 | 0 | 0.8 | 6.2 | 0.07 |
| 37 | 78.8 | 1.5 | 1.1 | 79.4 | 1.1 | 3 | 0 | -1.8 | 6.2 | 0.14 |
| 38 | 74.0 | 3.0 | 2.1 | 76.7 | 3.7 | 3 | 0 | -6.6 | 6.2 | 0.53 |
| 39 | 73.0 | 2.1 | 1.5 | 73.6 | 1.4 | 3 | 0 | -7.6 | 6.2 | 0.62 |
| 40 | 81.0 | 0.7 | 0.5 | 80.0 | 1.3 | 3 | 0 | 0.4 | 6.2 | 0.03 |
| 41 | 73.0 | 0.6 | 0.4 | 73.7 | 0.9 | 3 | 0 | -7.6 | 6.2 | 0.62 |
| 42 | 83.5 | 0.6 | 0.4 | 86.2 | 2.9 | 3 | 0 | 2.9 | 6.2 | 0.23 |
| 43 | 71.0 | 1.2 | 0.9 | 70.9 | 0.6 | 3 | 0 | -9.6 | 6.2 | 0.78 |
| 44 | 75.0 | 3.0 | 2.1 | 75.0 | 1.2 | 3 | 0 | -5.6 | 6.2 | 0.45 |
| 45 | 80.7 | 1.5 | 1.1 | 80.4 | 0.9 | 3 | 0 | 0.1 | 6.2 | 0.01 |
| 46 | 79.3 | 0.9 | 0.6 | 77.7 | 1.9 | 3 | 0 | -1.3 | 6.2 | 0.11 |
| 47 | 91.0 | 0.0 | 0.0 | 90.0 | 1.0 | 3 | 0 | 10.4 | 6.2 | 0.84 |
| 48 | 80.6 | 1.6 | 1.2 | 77.5 | 3.6 | 3 | 0 | 0.0 | 6.2 | 0.00 |
| 49 | 83.7 | 7.0 | 5.0 | 87.3 | 6.1 | 3 | 0 | 3.1 | 6.2 | 0.25 |
| 50 | 86.0 | 1.5 | 1.1 | 83.0 | 3.5 | 3 | 0 | 5.4 | 6.2 | 0.44 |
| 51 | 80.0 | 3.0 | 2.1 | 80.3 | 1.5 | 3 | 0 | -0.6 | 6.2 | 0.05 |
| 52 | 66.0 | 1.5 | 1.1 | 70.0 | 4.5 | 3 | 0 | -14.6 | 6.2 | 1.18 |
| 53 | 107.3 | 3.1 | 2.3 | 108.8 | 2.7 | 3 | 0 | 26.7 | 6.2 | 2.16 |
| 54 | 85.8 | 0.1 | 0.1 | 86.2 | 0.4 | 3 | 0 | 5.2 | 6.2 | 0.42 |
| 55 | 78.0 | 0.0 | 0.0 | 78.3 | 0.3 | 3 | 0 | -2.6 | 6.2 | 0.21 |
| 56 | 76.8 | 2.3 | 1.7 | 76.9 | 1.0 | 3 | 0 | -3.8 | 6.2 | 0.31 |
| 57 | 80.4 | 1.9 | 1.4 | 81.1 | 1.4 | 3 | 0 | -0.2 | 6.2 | 0.02 |
| 58 | 84.9 | 2.1 | 1.5 | 84.1 | 1.6 | 3 | 0 | 4.3 | 6.2 | 0.35 |
| 59 | 77.0 | 0.0 | 0.0 | 75.7 | 1.3 | 3 | 0 | -3.6 | 6.2 | 0.29 |
| 60 | 83.0 | 0.0 | 0.0 | 82.3 | 0.7 | 3 | 0 | 2.4 | 6.2 | 0.19 |
| **Histidine** | | | | | | | | | | |
| 61 | 93.0 | 1.6 | 1.2 | 95.7 | 3.3 | 3 | 0 | 12.4 | 6.2 | 1.00 |
| 62 | 77.0 | 1.5 | 1.1 | 76.7 | 0.9 | 3 | 0 | -3.6 | 6.2 | 0.29 |
| 63 | 79.1 | 1.5 | 1.1 | 79.0 | 0.6 | 3 | 0 | -1.5 | 6.2 | 0.12 |
| 64 | 84.0 | 1.5 | 1.1 | 81.0 | 3.5 | 3 | 0 | 3.4 | 6.2 | 0.28 |
| 65 | 88.5 | 3.1 | 2.2 | 89.0 | 1.7 | 3 | 0 | 7.9 | 6.2 | 0.64 |
| 66 | 87.0 | 0.9 | 0.7 | 86.0 | 1.4 | 3 | 0 | 6.4 | 6.2 | 0.52 |
| 67 | 74.0 | 2.2 | 1.6 | 71.4 | 3.4 | 3 | 0 | -6.6 | 6.2 | 0.53 |
| 68 | 82.8 | 2.3 | 1.7 | 85.6 | 3.6 | 3 | 0 | 2.2 | 6.2 | 0.18 |
| 69 | 97.1 | 27.3 | 19.7 | 98.8 | 12.2 | 3 | 0 | 16.5 | 6.2 | 1.33 |
| 70 | 75.0 | 0.0 | 0.0 | 73.0 | 2.0 | 3 | 0 | -5.6 | 6.2 | 0.45 |
| 71 | 73.0 | 11.9 | 8.6 | 73.0 | 4.6 | 3 | 0 | -7.6 | 6.2 | 0.62 |
| 72 | 76.0 | 0.0 | 0.0 | 76.3 | 0.3 | 3 | 0 | -4.6 | 6.2 | 0.37 |
| 73 | 75.0 | 1.5 | 1.1 | 75.0 | 0.6 | 3 | 0 | -5.6 | 6.2 | 0.45 |
| 74 | 81.2 | 6.8 | 4.9 | 80.8 | 3.0 | 3 | 0 | 0.6 | 6.2 | 0.05 |
| 75 | 88.4 | 1.0 | 0.8 | 89.6 | 1.6 | 3 | 0 | 7.8 | 6.2 | 0.63 |
| 76 | 83.5 | 3.3 | 2.4 | 87.6 | 5.2 | 3 | 0 | 2.9 | 6.2 | 0.23 |
| 77 | 75.0 | 7.4 | 5.4 | 76.7 | 4.4 | 3 | 0 | -5.6 | 6.2 | 0.45 |
| 78 | 72.0 | 0.0 | 0.0 | 73.3 | 1.3 | 3 | 0 | -8.6 | 6.2 | 0.70 |
| 79 | 76.0 | 1.5 | 1.1 | 76.0 | 0.6 | 3 | 0 | -4.6 | 6.2 | 0.37 |
| 80 | 47.0 | 1.5 | 1.1 | 47.3 | 0.9 | 3 | 0 | -33.6 | 6.2 | 2.72 |
| 81 | 85.2 | 1.0 | 0.8 | 84.9 | 0.6 | 3 | 0 | 4.6 | 6.2 | 0.37 |
| 83 | 64.1 | 0.6 | 0.4 | 63.6 | 0.7 | 3 | 0 | -16.5 | 6.2 | 1.34 |
| 84 | 54.9 | 0.4 | 0.3 | 54.6 | 0.5 | 3 | 0 | -25.7 | 6.2 | 2.08 |
| 85 | 81.0 | 3.0 | 2.1 | 81.7 | 1.8 | 3 | 0 | 0.4 | 6.2 | 0.03 |
| 86 | 79.0 | 1.0 | 0.8 | 79.1 | 0.5 | 3 | 0 | -1.6 | 6.2 | 0.13 |
| 87 | 79.0 | 4.4 | 3.2 | 79.7 | 2.3 | 3 | 0 | -1.6 | 6.2 | 0.13 |
| 88 | 83.1 | 1.6 | 1.1 | 79.6 | 4.0 | 3 | 0 | 2.5 | 6.2 | 0.20 |
| 89 | 82.0 | 3.0 | 2.1 | 81.0 | 2.1 | 3 | 0 | 1.4 | 6.2 | 0.11 |
| 90 | 89.4 | 0.4 | 0.3 | 89.8 | 0.6 | 3 | 0 | 8.8 | 6.2 | 0.71 |

**Isoleucine**

| **Lab** | **Median** | **MAD** | **u (median)** | **Mean** | **u (mean)** | **n** | **Non-detects** | **DoE** | **u (DoE)** | **Score** |
| --- | --- | --- | --- | --- | --- | --- | --- | --- | --- | --- |
| 1 | 90.9 | 2.1 | 1.5 | 90.6 | 1.0 | 3 | 0 | 9.9 | 5.5 | 0.90 |
| 2 | 75.1 | 0.4 | 0.3 | 77.8 | 2.9 | 3 | 0 | -5.9 | 5.5 | 0.54 |
| 3 | 76.0 | 3.0 | 2.1 | 76.3 | 1.5 | 3 | 0 | -5.0 | 5.5 | 0.46 |
| 4 | 81.5 | 0.7 | 0.6 | 81.5 | 0.5 | 2 | NA | 0.5 | 5.3 | 0.04 |
| 5 | 78.0 | 0.0 | 0.0 | 78.7 | 0.7 | 3 | 0 | -3.0 | 5.5 | 0.27 |
| 6 | 81.0 | 0.0 | 0.0 | 80.3 | 0.7 | 3 | 0 | 0.0 | 5.5 | 0.00 |
| 7 | 86.0 | 3.0 | 2.1 | 87.0 | 2.1 | 3 | 0 | 5.0 | 5.5 | 0.46 |
| 8 | 74.0 | 0.0 | 0.0 | 74.0 | 0.0 | 3 | 0 | -7.0 | 5.5 | 0.64 |
| 9 | 132.5 | 2.3 | 1.7 | 171.5 | 39.8 | 3 | 0 | 51.5 | 5.5 | 4.69 |
| 10 | 79.8 | 0.1 | 0.1 | 79.2 | 0.6 | 3 | 0 | -1.2 | 5.5 | 0.11 |
| 11 | 87.1 | 1.8 | 1.3 | 84.9 | 2.9 | 3 | 0 | 6.1 | 5.5 | 0.56 |
| 12 | 86.0 | 4.4 | 3.2 | 85.9 | 1.8 | 3 | 0 | 5.0 | 5.5 | 0.46 |
| **Isoleucine** | | | | | | | | | | |
| 13 | 84.0 | 0.0 | 0.0 | 85.0 | 1.0 | 3 | 0 | 3.0 | 5.5 | 0.27 |
| 14 | 88.0 | 0.0 | 0.0 | 86.7 | 1.3 | 3 | 0 | 7.0 | 5.5 | 0.64 |
| 15 | 78.3 | 0.7 | 0.5 | 78.5 | 0.4 | 3 | 0 | -2.7 | 5.5 | 0.25 |
| 16 | 81.0 | 0.0 | 0.0 | 80.7 | 0.3 | 3 | 0 | 0.0 | 5.5 | 0.00 |
| 17 | 78.0 | 3.0 | 2.1 | 78.7 | 1.8 | 3 | 0 | -3.0 | 5.5 | 0.27 |
| 18 | 83.0 | 1.5 | 1.1 | 82.7 | 0.9 | 3 | 0 | 2.0 | 5.5 | 0.18 |
| 19 | 92.1 | 1.7 | 1.2 | 91.6 | 1.1 | 3 | 0 | 11.1 | 5.5 | 1.01 |
| 20 | 79.4 | 4.2 | 3.0 | 80.6 | 2.7 | 3 | 0 | -1.6 | 5.5 | 0.15 |
| 21 | 86.0 | 0.0 | 0.0 | 86.3 | 0.3 | 3 | 0 | 5.0 | 5.5 | 0.46 |
| 22 | 90.0 | 1.5 | 1.1 | 89.0 | 1.5 | 3 | 0 | 9.0 | 5.5 | 0.82 |
| 23 | 77.3 | 0.1 | 0.1 | 75.3 | 2.1 | 3 | 0 | -3.7 | 5.5 | 0.34 |
| 24 | 78.0 | 0.0 | 0.0 | 79.7 | 1.7 | 3 | 0 | -3.0 | 5.5 | 0.27 |
| 25 | 86.0 | 0.0 | 0.0 | 84.7 | 1.3 | 3 | 0 | 5.0 | 5.5 | 0.46 |
| 26 | 65.2 | 1.3 | 1.0 | 65.2 | 0.6 | 3 | 0 | -15.8 | 5.5 | 1.44 |
| 27 | 83.6 | 0.6 | 0.4 | 82.8 | 1.0 | 3 | 0 | 2.6 | 5.5 | 0.24 |
| 28 | 66.0 | 0.0 | 0.0 | 64.7 | 1.3 | 3 | 0 | -15.0 | 5.5 | 1.37 |
| 29 | 80.7 | 1.8 | 1.3 | 82.6 | 2.5 | 3 | 0 | -0.3 | 5.5 | 0.03 |
| 30 | 84.0 | 7.9 | 5.7 | 86.4 | 5.2 | 3 | 0 | 3.0 | 5.5 | 0.27 |
| 31 | 83.0 | 1.5 | 1.1 | 83.0 | 0.6 | 3 | 0 | 2.0 | 5.5 | 0.18 |
| 32 | 85.4 | 1.6 | 1.2 | 85.0 | 1.0 | 3 | 0 | 4.4 | 5.5 | 0.40 |
| 33 | 78.0 | 3.0 | 2.1 | 78.0 | 1.2 | 3 | 0 | -3.0 | 5.5 | 0.27 |
| 34 | 74.0 | 0.6 | 0.4 | 75.2 | 1.4 | 3 | 0 | -7.0 | 5.5 | 0.64 |
| 35 | 81.0 | 1.5 | 1.1 | 81.7 | 1.2 | 3 | 0 | 0.0 | 5.5 | 0.00 |
| 36 | 77.6 | 2.1 | 1.5 | 77.6 | 0.9 | 3 | 0 | -3.4 | 5.5 | 0.31 |
| 37 | 77.2 | 0.5 | 0.4 | 77.7 | 0.6 | 3 | 0 | -3.8 | 5.5 | 0.34 |
| 38 | 85.0 | 3.0 | 2.1 | 84.7 | 1.5 | 3 | 0 | 4.0 | 5.5 | 0.36 |
| 39 | 72.5 | 0.6 | 0.4 | 73.8 | 1.5 | 3 | 0 | -8.5 | 5.5 | 0.77 |
| 40 | 81.0 | 2.2 | 1.6 | 82.0 | 1.8 | 3 | 0 | 0.0 | 5.5 | 0.00 |
| 41 | 75.5 | 0.1 | 0.1 | 76.0 | 0.6 | 3 | 0 | -5.5 | 5.5 | 0.50 |
| 42 | 77.7 | 0.9 | 0.6 | 77.4 | 0.6 | 3 | 0 | -3.3 | 5.5 | 0.30 |
| 43 | 69.5 | 1.7 | 1.2 | 70.3 | 1.4 | 3 | 0 | -11.5 | 5.5 | 1.05 |
| 44 | 78.0 | 0.0 | 0.0 | 78.7 | 0.7 | 3 | 0 | -3.0 | 5.5 | 0.27 |
| 45 | 81.4 | 0.0 | 0.0 | 80.9 | 0.5 | 3 | 0 | 0.4 | 5.5 | 0.04 |
| 46 | 80.6 | 0.1 | 0.1 | 81.3 | 0.8 | 3 | 0 | -0.4 | 5.5 | 0.04 |
| 47 | 78.0 | 3.0 | 2.1 | 80.3 | 3.4 | 3 | 0 | -3.0 | 5.5 | 0.27 |
| 48 | 78.3 | 1.2 | 0.9 | 78.9 | 1.0 | 3 | 0 | -2.7 | 5.5 | 0.25 |
| 49 | 88.2 | 1.6 | 1.2 | 89.5 | 1.9 | 3 | 0 | 7.2 | 5.5 | 0.66 |
| 50 | 72.0 | 3.0 | 2.1 | 72.3 | 1.5 | 3 | 0 | -9.0 | 5.5 | 0.82 |
| 51 | 85.0 | 3.0 | 2.1 | 85.0 | 1.2 | 3 | 0 | 4.0 | 5.5 | 0.36 |
| 52 | 83.0 | 1.5 | 1.1 | 85.3 | 2.8 | 3 | 0 | 2.0 | 5.5 | 0.18 |
| 53 | 68.0 | 0.6 | 0.4 | 62.9 | 5.3 | 3 | 0 | -13.0 | 5.5 | 1.18 |
| 54 | 85.6 | 0.5 | 0.3 | 85.1 | 0.7 | 3 | 0 | 4.6 | 5.5 | 0.42 |
| 55 | 80.0 | 0.0 | 0.0 | 79.7 | 0.3 | 3 | 0 | -1.0 | 5.5 | 0.09 |
| 56 | 80.1 | 1.7 | 1.2 | 80.0 | 0.7 | 3 | 0 | -0.9 | 5.5 | 0.08 |
| 57 | 82.8 | 2.2 | 1.6 | 82.5 | 1.2 | 3 | 0 | 1.8 | 5.5 | 0.16 |
| **Isoleucine** | | | | | | | | | | |
| 58 | 86.2 | 3.1 | 2.3 | 86.2 | 1.2 | 3 | 0 | 5.2 | 5.5 | 0.47 |
| 59 | 75.0 | 1.5 | 1.1 | 74.0 | 1.5 | 3 | 0 | -6.0 | 5.5 | 0.55 |
| 60 | 69.0 | 3.0 | 2.1 | 71.0 | 3.1 | 3 | 0 | -12.0 | 5.5 | 1.09 |
| 61 | 68.7 | 0.6 | 0.4 | 73.0 | 4.5 | 3 | 0 | -12.3 | 5.5 | 1.12 |
| 62 | 76.0 | 3.0 | 2.1 | 78.0 | 3.1 | 3 | 0 | -5.0 | 5.5 | 0.46 |
| 63 | 83.1 | 0.7 | 0.5 | 83.1 | 0.3 | 3 | 0 | 2.1 | 5.5 | 0.19 |
| 64 | 79.0 | 4.4 | 3.2 | 77.7 | 3.0 | 3 | 0 | -2.0 | 5.5 | 0.18 |
| 65 | 81.8 | 4.6 | 3.4 | 82.0 | 2.0 | 3 | 0 | 0.8 | 5.5 | 0.07 |
| 66 | 99.6 | 3.7 | 2.7 | 100.2 | 2.0 | 3 | 0 | 18.6 | 5.5 | 1.69 |
| 67 | 82.0 | 1.0 | 0.8 | 78.5 | 3.9 | 3 | 0 | 1.0 | 5.5 | 0.09 |
| 68 | 84.8 | 1.8 | 1.3 | 86.6 | 2.4 | 3 | 0 | 3.8 | 5.5 | 0.35 |
| 69 | 75.7 | 0.5 | 0.4 | 77.2 | 1.7 | 3 | 0 | -5.3 | 5.5 | 0.48 |
| 70 | 80.0 | 1.5 | 1.1 | 77.7 | 2.8 | 3 | 0 | -1.0 | 5.5 | 0.09 |
| 71 | 76.0 | 1.5 | 1.1 | 80.0 | 4.5 | 3 | 0 | -5.0 | 5.5 | 0.46 |
| 72 | 85.0 | 4.4 | 3.2 | 84.0 | 2.6 | 3 | 0 | 4.0 | 5.5 | 0.36 |
| 73 | 79.0 | 0.0 | 0.0 | 78.3 | 0.7 | 3 | 0 | -2.0 | 5.5 | 0.18 |
| 74 | 81.6 | 1.8 | 1.3 | 81.6 | 0.7 | 3 | 0 | 0.6 | 5.5 | 0.05 |
| 75 | 86.8 | 0.9 | 0.6 | 88.2 | 1.7 | 3 | 0 | 5.8 | 5.5 | 0.53 |
| 76 | 80.0 | 2.8 | 2.0 | 80.5 | 1.5 | 3 | 0 | -1.0 | 5.5 | 0.09 |
| 77 | 82.0 | 5.9 | 4.3 | 82.0 | 2.3 | 3 | 0 | 1.0 | 5.5 | 0.09 |
| 78 | 81.0 | 4.4 | 3.2 | 80.7 | 2.0 | 3 | 0 | 0.0 | 5.5 | 0.00 |
| 79 | 91.0 | 0.0 | 0.0 | 90.7 | 0.3 | 3 | 0 | 10.0 | 5.5 | 0.91 |
| 80 | 50.0 | 0.0 | 0.0 | 48.3 | 1.7 | 3 | 0 | -31.0 | 5.5 | 2.82 |
| 81 | 90.6 | 0.6 | 0.4 | 90.3 | 0.5 | 3 | 0 | 9.6 | 5.5 | 0.87 |
| 83 | 63.7 | 1.9 | 1.4 | 63.9 | 0.9 | 3 | 0 | -17.3 | 5.5 | 1.58 |
| 84 | 73.0 | 2.7 | 2.0 | 72.3 | 1.8 | 3 | 0 | -8.0 | 5.5 | 0.73 |
| 85 | 85.0 | 7.4 | 5.4 | 87.3 | 5.0 | 3 | 0 | 4.0 | 5.5 | 0.36 |
| 86 | 83.0 | 1.5 | 1.1 | 83.7 | 1.2 | 3 | 0 | 2.0 | 5.5 | 0.18 |
| 87 | 81.0 | 1.5 | 1.1 | 80.0 | 1.5 | 3 | 0 | 0.0 | 5.5 | 0.00 |
| 88 | 87.1 | 3.5 | 2.5 | 88.6 | 2.7 | 3 | 0 | 6.1 | 5.5 | 0.56 |
| 89 | 96.0 | 3.0 | 2.1 | 92.3 | 4.7 | 3 | 0 | 15.0 | 5.5 | 1.37 |
| 90 | 91.0 | 0.9 | 0.6 | 91.0 | 0.4 | 3 | 0 | 10.0 | 5.5 | 0.91 |

**Leucine**

| **Lab** | **Median** | **MAD** | **u (median)** | **Mean** | **u (mean)** | **n** | **Non-detects** | **DoE** | **u (DoE)** | **Score** |
| --- | --- | --- | --- | --- | --- | --- | --- | --- | --- | --- |
| 1 | 157.7 | 1.5 | 1.1 | 156.3 | 1.9 | 3 | 0 | 8.7 | 8.6 | 0.50 |
| 2 | 135.0 | 1.5 | 1.1 | 136.3 | 1.9 | 3 | 0 | -14.0 | 8.6 | 0.81 |
| 3 | 144.0 | 1.5 | 1.1 | 146.7 | 3.2 | 3 | 0 | -5.0 | 8.6 | 0.29 |
| 4 | 184.0 | 0.0 | 0.0 | 182.0 | 2.0 | 3 | 0 | 35.0 | 8.6 | 2.02 |
| 5 | 142.0 | 0.0 | 0.0 | 142.3 | 0.3 | 3 | 0 | -7.0 | 8.6 | 0.40 |
| 6 | 153.0 | 1.5 | 1.1 | 153.0 | 0.6 | 3 | 0 | 4.0 | 8.6 | 0.23 |
| 7 | 172.0 | 4.4 | 3.2 | 164.3 | 9.2 | 3 | 0 | 23.0 | 8.6 | 1.33 |
| 8 | 143.0 | 0.0 | 0.0 | 142.0 | 1.0 | 3 | 0 | -6.0 | 8.6 | 0.35 |
| 9 | 143.2 | 26.1 | 18.9 | 167.4 | 33.5 | 3 | 0 | -5.8 | 8.6 | 0.34 |
| **Leucine** | | | | | | | | | | |
| 10 | 148.6 | 3.1 | 2.3 | 148.1 | 1.7 | 3 | 0 | -0.4 | 8.6 | 0.03 |
| 11 | 162.0 | 4.4 | 3.2 | 159.7 | 3.9 | 3 | 0 | 13.0 | 8.6 | 0.75 |
| 12 | 148.1 | 0.1 | 0.1 | 151.0 | 3.0 | 3 | 0 | -0.9 | 8.6 | 0.05 |
| 13 | 156.0 | 0.0 | 0.0 | 157.3 | 1.3 | 3 | 0 | 7.0 | 8.6 | 0.40 |
| 14 | 147.0 | 1.5 | 1.1 | 147.3 | 0.9 | 3 | 0 | -2.0 | 8.6 | 0.12 |
| 15 | 145.5 | 1.3 | 0.9 | 145.3 | 0.7 | 3 | 0 | -3.6 | 8.6 | 0.21 |
| 16 | 150.0 | 1.5 | 1.1 | 149.7 | 0.9 | 3 | 0 | 1.0 | 8.6 | 0.06 |
| 17 | 149.0 | 0.0 | 0.0 | 150.0 | 1.0 | 3 | 0 | 0.0 | 8.6 | 0.00 |
| 18 | 153.0 | 0.0 | 0.0 | 152.3 | 0.7 | 3 | 0 | 4.0 | 8.6 | 0.23 |
| 19 | 164.7 | 4.1 | 3.0 | 163.6 | 2.6 | 3 | 0 | 15.7 | 8.6 | 0.91 |
| 20 | 146.7 | 6.7 | 4.8 | 148.5 | 4.3 | 3 | 0 | -2.3 | 8.6 | 0.13 |
| 21 | 159.0 | 3.0 | 2.1 | 158.3 | 1.8 | 3 | 0 | 10.0 | 8.6 | 0.58 |
| 22 | 165.0 | 1.5 | 1.1 | 163.3 | 2.2 | 3 | 0 | 16.0 | 8.6 | 0.93 |
| 23 | 140.8 | 1.5 | 1.1 | 140.1 | 1.3 | 3 | 0 | -8.2 | 8.6 | 0.47 |
| 24 | 143.0 | 0.0 | 0.0 | 146.3 | 3.3 | 3 | 0 | -6.0 | 8.6 | 0.35 |
| 25 | 149.0 | 1.5 | 1.1 | 148.7 | 0.9 | 3 | 0 | 0.0 | 8.6 | 0.00 |
| 26 | 127.3 | 2.5 | 1.8 | 126.6 | 1.6 | 3 | 0 | -21.7 | 8.6 | 1.26 |
| 27 | 159.9 | 1.2 | 0.9 | 159.0 | 1.3 | 3 | 0 | 10.9 | 8.6 | 0.63 |
| 28 | 122.0 | 1.5 | 1.1 | 122.7 | 1.2 | 3 | 0 | -27.0 | 8.6 | 1.56 |
| 29 | 138.9 | 3.7 | 2.7 | 140.4 | 2.8 | 3 | 0 | -10.1 | 8.6 | 0.58 |
| 30 | 152.8 | 7.9 | 5.7 | 153.4 | 3.6 | 3 | 0 | 3.8 | 8.6 | 0.22 |
| 31 | 150.0 | 0.0 | 0.0 | 151.7 | 1.7 | 3 | 0 | 1.0 | 8.6 | 0.06 |
| 32 | 162.7 | 0.0 | 0.0 | 162.2 | 0.5 | 3 | 0 | 13.7 | 8.6 | 0.79 |
| 33 | 147.0 | 1.5 | 1.1 | 145.7 | 1.9 | 3 | 0 | -2.0 | 8.6 | 0.12 |
| 34 | 112.0 | 10.4 | 7.5 | 112.3 | 4.3 | 3 | 0 | -37.0 | 8.6 | 2.14 |
| 35 | 152.0 | 1.5 | 1.1 | 153.0 | 1.5 | 3 | 0 | 3.0 | 8.6 | 0.17 |
| 36 | 150.2 | 0.5 | 0.4 | 148.8 | 1.5 | 3 | 0 | 1.2 | 8.6 | 0.07 |
| 37 | 145.3 | 0.8 | 0.6 | 146.0 | 1.0 | 3 | 0 | -3.7 | 8.6 | 0.21 |
| 38 | 151.0 | 7.4 | 5.4 | 152.3 | 4.1 | 3 | 0 | 2.0 | 8.6 | 0.12 |
| 39 | 138.0 | 4.4 | 3.2 | 138.0 | 1.7 | 3 | 0 | -11.0 | 8.6 | 0.64 |
| 40 | 153.5 | 3.7 | 2.7 | 154.7 | 2.5 | 3 | 0 | 4.5 | 8.6 | 0.26 |
| 41 | 141.1 | 1.6 | 1.2 | 141.5 | 1.0 | 3 | 0 | -7.9 | 8.6 | 0.46 |
| 42 | 155.3 | 5.2 | 3.8 | 157.3 | 3.9 | 3 | 0 | 6.3 | 8.6 | 0.36 |
| 43 | 146.6 | 0.5 | 0.4 | 145.1 | 1.6 | 3 | 0 | -2.4 | 8.6 | 0.14 |
| 44 | 152.0 | 0.0 | 0.0 | 152.7 | 0.7 | 3 | 0 | 3.0 | 8.6 | 0.17 |
| 45 | 148.4 | 2.1 | 1.5 | 147.0 | 2.2 | 3 | 0 | -0.6 | 8.6 | 0.03 |
| 46 | 151.7 | 0.3 | 0.2 | 154.1 | 2.5 | 3 | 0 | 2.7 | 8.6 | 0.16 |
| 47 | 167.0 | 1.5 | 1.1 | 163.3 | 4.2 | 3 | 0 | 18.0 | 8.6 | 1.04 |
| 48 | 145.8 | 1.5 | 1.1 | 145.1 | 1.2 | 3 | 0 | -3.2 | 8.6 | 0.19 |
| 49 | 161.0 | 5.9 | 4.3 | 161.7 | 2.9 | 3 | 0 | 12.0 | 8.6 | 0.69 |
| 50 | 162.0 | 4.4 | 3.2 | 159.7 | 3.9 | 3 | 0 | 13.0 | 8.6 | 0.75 |
| 51 | 147.0 | 1.5 | 1.1 | 147.7 | 1.2 | 3 | 0 | -2.0 | 8.6 | 0.12 |
| 52 | 150.0 | 0.0 | 0.0 | 154.7 | 4.7 | 3 | 0 | 1.0 | 8.6 | 0.06 |
| 53 | 178.0 | 10.5 | 7.6 | 181.8 | 7.7 | 3 | 0 | 29.0 | 8.6 | 1.68 |
| 54 | 144.2 | 0.8 | 0.6 | 144.4 | 0.5 | 3 | 0 | -4.8 | 8.6 | 0.28 |
| **Leucine** | | | | | | | | | | |
| 55 | 147.0 | 1.5 | 1.1 | 147.0 | 0.6 | 3 | 0 | -2.0 | 8.6 | 0.12 |
| 56 | 130.9 | 0.2 | 0.1 | 132.9 | 2.0 | 3 | 0 | -18.1 | 8.6 | 1.04 |
| 57 | 149.1 | 3.0 | 2.1 | 149.4 | 1.4 | 3 | 0 | 0.1 | 8.6 | 0.01 |
| 58 | 157.5 | 1.3 | 0.9 | 155.5 | 2.5 | 3 | 0 | 8.5 | 8.6 | 0.49 |
| 59 | 153.0 | 3.0 | 2.1 | 150.7 | 3.4 | 3 | 0 | 4.0 | 8.6 | 0.23 |
| 60 | 103.0 | 1.5 | 1.1 | 104.0 | 1.5 | 3 | 0 | -46.0 | 8.6 | 2.66 |
| 61 | 122.1 | 9.6 | 7.0 | 122.4 | 4.0 | 3 | 0 | -26.9 | 8.6 | 1.56 |
| 62 | 138.0 | 3.0 | 2.1 | 140.3 | 3.4 | 3 | 0 | -11.0 | 8.6 | 0.64 |
| 63 | 146.2 | 0.4 | 0.3 | 146.6 | 0.6 | 3 | 0 | -2.8 | 8.6 | 0.16 |
| 64 | 142.0 | 11.9 | 8.6 | 147.0 | 9.3 | 3 | 0 | -7.0 | 8.6 | 0.40 |
| 65 | 144.8 | 0.9 | 0.7 | 147.2 | 2.7 | 3 | 0 | -4.2 | 8.6 | 0.24 |
| 66 | 180.0 | 1.5 | 1.1 | 181.3 | 1.9 | 3 | 0 | 31.0 | 8.6 | 1.79 |
| 67 | 148.0 | 1.5 | 1.1 | 142.3 | 6.2 | 3 | 0 | -1.0 | 8.6 | 0.06 |
| 68 | 156.7 | 1.3 | 0.9 | 161.1 | 4.8 | 3 | 0 | 7.7 | 8.6 | 0.44 |
| 69 | 167.1 | 1.9 | 1.4 | 165.9 | 1.8 | 3 | 0 | 18.1 | 8.6 | 1.04 |
| 70 | 140.0 | 5.9 | 4.3 | 138.3 | 3.8 | 3 | 0 | -9.0 | 8.6 | 0.52 |
| 71 | 130.0 | 1.5 | 1.1 | 134.7 | 5.2 | 3 | 0 | -19.0 | 8.6 | 1.10 |
| 72 | 143.0 | 1.5 | 1.1 | 142.0 | 1.5 | 3 | 0 | -6.0 | 8.6 | 0.35 |
| 73 | 144.0 | 1.5 | 1.1 | 143.3 | 1.2 | 3 | 0 | -5.0 | 8.6 | 0.29 |
| 74 | 163.7 | 4.7 | 3.4 | 165.4 | 3.4 | 3 | 0 | 14.7 | 8.6 | 0.85 |
| 75 | 159.2 | 3.1 | 2.3 | 158.7 | 1.7 | 3 | 0 | 10.2 | 8.6 | 0.59 |
| 76 | 169.3 | 3.3 | 2.4 | 167.3 | 3.1 | 3 | 0 | 20.3 | 8.6 | 1.17 |
| 77 | 145.0 | 5.9 | 4.3 | 146.3 | 3.5 | 3 | 0 | -4.0 | 8.6 | 0.23 |
| 78 | 141.0 | 7.4 | 5.4 | 141.7 | 3.5 | 3 | 0 | -8.0 | 8.6 | 0.46 |
| 79 | 153.0 | 0.0 | 0.0 | 152.3 | 0.7 | 3 | 0 | 4.0 | 8.6 | 0.23 |
| 80 | 86.0 | 3.0 | 2.1 | 86.0 | 1.2 | 3 | 0 | -63.0 | 8.6 | 3.64 |
| 81 | 150.7 | 2.7 | 1.9 | 150.1 | 1.6 | 3 | 0 | 1.7 | 8.6 | 0.10 |
| 83 | 150.1 | 4.4 | 3.2 | 150.2 | 1.8 | 3 | 0 | 1.1 | 8.6 | 0.06 |
| 84 | 142.6 | 7.8 | 5.7 | 142.8 | 3.3 | 3 | 0 | -6.4 | 8.6 | 0.37 |
| 85 | 160.0 | 13.3 | 9.7 | 157.0 | 7.9 | 3 | 0 | 11.0 | 8.6 | 0.64 |
| 86 | 160.0 | 3.6 | 2.6 | 160.9 | 2.2 | 3 | 0 | 11.0 | 8.6 | 0.64 |
| 87 | 145.0 | 1.5 | 1.1 | 146.0 | 1.5 | 3 | 0 | -4.0 | 8.6 | 0.23 |
| 88 | 148.2 | 10.8 | 7.8 | 149.2 | 5.1 | 3 | 0 | -0.8 | 8.6 | 0.05 |
| 89 | 159.0 | 3.0 | 2.1 | 154.7 | 5.4 | 3 | 0 | 10.0 | 8.6 | 0.58 |
| 90 | 158.6 | 1.2 | 0.9 | 157.9 | 1.1 | 3 | 0 | 9.6 | 8.6 | 0.56 |

**Lysine**

| **Lab** | **Median** | **MAD** | **u (median)** | **Mean** | **u (mean)** | **n** | **Non-detects** | **DoE** | **u (DoE)** | **Score** |
| --- | --- | --- | --- | --- | --- | --- | --- | --- | --- | --- |
| 1 | 179.4 | 1.8 | 1.3 | 179.6 | 0.9 | 3 | 0 | 3.4 | 11.0 | 0.15 |
| 2 | 172.0 | 1.5 | 1.1 | 173.3 | 1.9 | 3 | 0 | -4.0 | 11.0 | 0.18 |
| 3 | 169.0 | 3.0 | 2.1 | 172.7 | 4.7 | 3 | 0 | -7.0 | 11.0 | 0.32 |
| 4 | 174.0 | 5.9 | 4.3 | 171.3 | 4.8 | 3 | 0 | -2.0 | 11.0 | 0.09 |
| 5 | 180.0 | 5.9 | 4.3 | 178.7 | 3.5 | 3 | 0 | 4.0 | 11.0 | 0.18 |
| 6 | 175.0 | 3.0 | 2.1 | 175.3 | 1.5 | 3 | 0 | -1.0 | 11.0 | 0.05 |
| 7 | 179.0 | 4.4 | 3.2 | 178.0 | 2.6 | 3 | 0 | 3.0 | 11.0 | 0.14 |
| 8 | 172.0 | 1.5 | 1.1 | 171.7 | 0.9 | 3 | 0 | -4.0 | 11.0 | 0.18 |
| 9 | 189.0 | 36.9 | 26.7 | 236.8 | 60.6 | 3 | 0 | 13.0 | 11.0 | 0.59 |
| 10 | 181.4 | 0.7 | 0.5 | 180.3 | 1.3 | 3 | 0 | 5.4 | 11.0 | 0.25 |
| 11 | 170.0 | 8.9 | 6.4 | 173.7 | 6.9 | 3 | 0 | -6.0 | 11.0 | 0.27 |
| 12 | 170.6 | 11.2 | 8.1 | 171.2 | 4.9 | 3 | 0 | -5.4 | 11.0 | 0.25 |
| 13 | 173.0 | 4.4 | 3.2 | 172.3 | 2.3 | 3 | 0 | -3.0 | 11.0 | 0.14 |
| 14 | 172.0 | 5.9 | 4.3 | 172.0 | 2.3 | 3 | 0 | -4.0 | 11.0 | 0.18 |
| 15 | 175.8 | 0.7 | 0.5 | 175.0 | 1.1 | 3 | 0 | -0.3 | 11.0 | 0.01 |
| 16 | 184.0 | 4.4 | 3.2 | 184.0 | 1.7 | 3 | 0 | 8.0 | 11.0 | 0.36 |
| 17 | 184.0 | 0.0 | 0.0 | 184.3 | 0.3 | 3 | 0 | 8.0 | 11.0 | 0.36 |
| 18 | 172.0 | 1.5 | 1.1 | 173.3 | 1.9 | 3 | 0 | -4.0 | 11.0 | 0.18 |
| 19 | 182.6 | 5.4 | 3.9 | 183.8 | 3.2 | 3 | 0 | 6.6 | 11.0 | 0.30 |
| 20 | 169.0 | 13.8 | 10.0 | 169.3 | 5.7 | 3 | 0 | -7.0 | 11.0 | 0.32 |
| 21 | 183.0 | 1.5 | 1.1 | 183.3 | 0.9 | 3 | 0 | 7.0 | 11.0 | 0.32 |
| 22 | 196.0 | 0.0 | 0.0 | 194.0 | 2.0 | 3 | 0 | 20.0 | 11.0 | 0.91 |
| 23 | 166.0 | 2.7 | 1.9 | 166.5 | 1.5 | 3 | 0 | -10.0 | 11.0 | 0.46 |
| 24 | 172.0 | 0.0 | 0.0 | 176.3 | 4.3 | 3 | 0 | -4.0 | 11.0 | 0.18 |
| 25 | 182.0 | 3.0 | 2.1 | 182.3 | 1.5 | 3 | 0 | 6.0 | 11.0 | 0.27 |
| 26 | 168.0 | 6.8 | 5.0 | 163.2 | 7.2 | 3 | 0 | -8.0 | 11.0 | 0.37 |
| 27 | 189.3 | 3.5 | 2.5 | 187.6 | 2.9 | 3 | 0 | 13.3 | 11.0 | 0.60 |
| 28 | 143.0 | 3.0 | 2.1 | 144.3 | 2.4 | 3 | 0 | -33.0 | 11.0 | 1.50 |
| 29 | 169.5 | 7.2 | 5.2 | 169.1 | 3.1 | 3 | 0 | -6.5 | 11.0 | 0.30 |
| 30 | 177.9 | 14.1 | 10.2 | 177.2 | 6.1 | 3 | 0 | 1.9 | 11.0 | 0.09 |
| 31 | 158.0 | 19.3 | 13.9 | 151.0 | 14.0 | 3 | 0 | -18.0 | 11.0 | 0.82 |
| 32 | 177.3 | 0.6 | 0.4 | 177.8 | 0.7 | 3 | 0 | 1.3 | 11.0 | 0.06 |
| 33 | 182.0 | 5.9 | 4.3 | 182.0 | 2.3 | 3 | 0 | 6.0 | 11.0 | 0.27 |
| 34 | 189.0 | 14.8 | 10.7 | 192.0 | 8.5 | 3 | 0 | 13.0 | 11.0 | 0.59 |
| 35 | 176.0 | 1.5 | 1.1 | 174.7 | 1.9 | 3 | 0 | 0.0 | 11.0 | 0.00 |
| 36 | 180.0 | 1.4 | 1.0 | 181.3 | 1.8 | 3 | 0 | 4.0 | 11.0 | 0.18 |
| 37 | 206.5 | 3.4 | 2.5 | 206.4 | 1.4 | 3 | 0 | 30.5 | 11.0 | 1.39 |
| 38 | 188.0 | 1.5 | 1.1 | 186.3 | 2.2 | 3 | 0 | 12.0 | 11.0 | 0.55 |
| 39 | 144.0 | 0.0 | 0.0 | 144.3 | 0.3 | 3 | 0 | -32.0 | 11.0 | 1.46 |
| 40 | 187.5 | 6.7 | 4.8 | 187.3 | 2.7 | 3 | 0 | 11.5 | 11.0 | 0.52 |
| 41 | 161.2 | 3.0 | 2.1 | 160.9 | 1.4 | 3 | 0 | -14.8 | 11.0 | 0.67 |
| 42 | 176.1 | 21.9 | 15.9 | 173.6 | 10.8 | 3 | 0 | 0.1 | 11.0 | 0.00 |
| 43 | 159.1 | 3.6 | 2.6 | 159.2 | 1.5 | 3 | 0 | -16.9 | 11.0 | 0.77 |
| 44 | 186.0 | 0.0 | 0.0 | 185.3 | 0.7 | 3 | 0 | 10.0 | 11.0 | 0.46 |
| **Lysine** | | | | | | | | | | |
| 45 | 198.2 | 2.8 | 2.0 | 191.0 | 8.2 | 3 | 0 | 22.2 | 11.0 | 1.01 |
| 46 | 176.5 | 7.7 | 5.6 | 174.2 | 5.2 | 3 | 0 | 0.5 | 11.0 | 0.02 |
| 47 | 179.0 | 3.0 | 2.1 | 178.0 | 2.1 | 3 | 0 | 3.0 | 11.0 | 0.14 |
| 48 | 168.7 | 4.9 | 3.5 | 168.9 | 2.1 | 3 | 0 | -7.3 | 11.0 | 0.33 |
| 49 | 176.0 | 5.9 | 4.3 | 176.3 | 2.6 | 3 | 0 | 0.0 | 11.0 | 0.00 |
| 50 | 200.0 | 0.0 | 0.0 | 194.7 | 5.3 | 3 | 0 | 24.0 | 11.0 | 1.09 |
| 51 | 173.0 | 3.0 | 2.1 | 172.0 | 2.1 | 3 | 0 | -3.0 | 11.0 | 0.14 |
| 52 | 150.0 | 1.5 | 1.1 | 157.0 | 7.5 | 3 | 0 | -26.0 | 11.0 | 1.18 |
| 53 | 210.9 | 9.9 | 7.2 | 206.5 | 8.0 | 3 | 0 | 34.9 | 11.0 | 1.59 |
| 54 | 162.2 | 1.7 | 1.2 | 161.3 | 1.5 | 3 | 0 | -13.8 | 11.0 | 0.63 |
| 55 | 175.0 | 1.5 | 1.1 | 175.0 | 0.6 | 3 | 0 | -1.0 | 11.0 | 0.05 |
| 56 | 166.8 | 1.9 | 1.4 | 164.8 | 2.7 | 3 | 0 | -9.2 | 11.0 | 0.42 |
| 57 | 176.9 | 5.5 | 4.0 | 176.7 | 2.3 | 3 | 0 | 0.9 | 11.0 | 0.04 |
| 58 | 201.2 | 1.1 | 0.8 | 198.1 | 3.4 | 3 | 0 | 25.2 | 11.0 | 1.15 |
| 59 | 173.0 | 4.4 | 3.2 | 171.3 | 3.3 | 3 | 0 | -3.0 | 11.0 | 0.14 |
| 60 | 174.0 | 5.9 | 4.3 | 176.0 | 4.2 | 3 | 0 | -2.0 | 11.0 | 0.09 |
| 61 | 195.1 | 0.9 | 0.6 | 195.4 | 0.6 | 3 | 0 | 19.1 | 11.0 | 0.87 |
| 62 | 190.0 | 4.4 | 3.2 | 193.0 | 4.6 | 3 | 0 | 14.0 | 11.0 | 0.64 |
| 63 | 173.2 | 0.9 | 0.6 | 174.1 | 1.2 | 3 | 0 | -2.8 | 11.0 | 0.13 |
| 64 | 165.0 | 0.0 | 0.0 | 161.3 | 3.7 | 3 | 0 | -11.0 | 11.0 | 0.50 |
| 65 | 165.4 | 13.4 | 9.7 | 166.1 | 5.8 | 3 | 0 | -10.6 | 11.0 | 0.48 |
| 66 | 199.0 | 0.0 | 0.0 | 201.3 | 2.3 | 3 | 0 | 23.0 | 11.0 | 1.05 |
| 67 | 180.0 | 3.0 | 2.1 | 173.7 | 7.4 | 3 | 0 | 4.0 | 11.0 | 0.18 |
| 68 | 184.0 | 6.8 | 4.9 | 188.6 | 7.0 | 3 | 0 | 8.0 | 11.0 | 0.36 |
| 69 | 195.2 | 2.5 | 1.8 | 195.9 | 1.6 | 3 | 0 | 19.2 | 11.0 | 0.87 |
| 70 | 170.0 | 7.4 | 5.4 | 168.3 | 4.4 | 3 | 0 | -6.0 | 11.0 | 0.27 |
| 71 | 144.0 | 3.0 | 2.1 | 144.3 | 1.5 | 3 | 0 | -32.0 | 11.0 | 1.46 |
| 72 | 164.0 | 0.0 | 0.0 | 165.7 | 1.7 | 3 | 0 | -12.0 | 11.0 | 0.55 |
| 73 | 168.0 | 1.5 | 1.1 | 168.0 | 0.6 | 3 | 0 | -8.0 | 11.0 | 0.36 |
| 74 | 186.4 | 0.1 | 0.1 | 184.9 | 1.5 | 3 | 0 | 10.4 | 11.0 | 0.47 |
| 75 | 186.7 | 3.4 | 2.5 | 187.2 | 1.8 | 3 | 0 | 10.7 | 11.0 | 0.49 |
| 76 | 190.6 | 19.3 | 13.9 | 191.5 | 8.3 | 3 | 0 | 14.6 | 11.0 | 0.66 |
| 77 | 158.0 | 11.9 | 8.6 | 159.0 | 5.5 | 3 | 0 | -18.0 | 11.0 | 0.82 |
| 78 | 172.0 | 10.4 | 7.5 | 172.3 | 4.3 | 3 | 0 | -4.0 | 11.0 | 0.18 |
| 79 | 178.0 | 0.0 | 0.0 | 177.0 | 1.0 | 3 | 0 | 2.0 | 11.0 | 0.09 |
| 80 | 101.0 | 0.0 | 0.0 | 100.7 | 0.3 | 3 | 0 | -75.0 | 11.0 | 3.42 |
| 81 | 171.0 | 0.9 | 0.6 | 170.4 | 0.9 | 3 | 0 | -5.0 | 11.0 | 0.23 |
| 83 | 108.8 | 1.3 | 1.0 | 110.8 | 2.5 | 3 | 0 | -67.2 | 11.0 | 3.06 |
| 84 | 175.4 | 2.2 | 1.6 | 174.0 | 2.1 | 3 | 0 | -0.6 | 11.0 | 0.03 |
| 85 | 156.0 | 7.4 | 5.4 | 160.3 | 7.0 | 3 | 0 | -20.0 | 11.0 | 0.91 |
| 86 | 200.0 | 11.6 | 8.4 | 200.1 | 4.6 | 3 | 0 | 24.0 | 11.0 | 1.09 |
| 87 | 178.0 | 5.9 | 4.3 | 178.3 | 2.6 | 3 | 0 | 2.0 | 11.0 | 0.09 |
| 88 | 165.7 | 5.0 | 3.6 | 168.0 | 4.1 | 3 | 0 | -10.3 | 11.0 | 0.47 |
| 89 | 189.0 | 3.0 | 2.1 | 182.7 | 7.4 | 3 | 0 | 13.0 | 11.0 | 0.59 |
| 90 | 194.4 | 1.0 | 0.8 | 192.7 | 2.1 | 3 | 0 | 18.4 | 11.0 | 0.84 |

**Methionine**

| **Lab** | **Median** | **MAD** | **u (median)** | **Mean** | **u (mean)** | **n** | **Non-detects** | **DoE** | **u (DoE)** | **Score** |
| --- | --- | --- | --- | --- | --- | --- | --- | --- | --- | --- |
| 1 | 25.5 | 0.3 | 0.2 | 26.4 | 1.0 | 3 | 0 | -1.6 | 2.3 | 0.34 |
| 2 | 26.0 | 1.0 | 0.8 | 26.0 | 0.4 | 3 | 0 | -1.1 | 2.3 | 0.24 |
| 3 | 25.0 | 0.0 | 0.0 | 24.7 | 0.3 | 3 | 0 | -2.1 | 2.3 | 0.45 |
| 4 | 34.9 | 1.2 | 0.9 | 34.8 | 0.5 | 3 | 0 | 7.8 | 2.3 | 1.67 |
| 5 | 24.0 | 1.5 | 1.1 | 23.0 | 1.5 | 3 | 0 | -3.1 | 2.3 | 0.66 |
| 6 | 24.0 | 0.0 | 0.0 | 24.7 | 0.7 | 3 | 0 | -3.1 | 2.3 | 0.66 |
| 7 | 30.0 | 0.0 | 0.0 | 29.3 | 0.7 | 3 | 0 | 2.9 | 2.3 | 0.62 |
| 8 | 26.0 | 0.0 | 0.0 | 25.7 | 0.3 | 3 | 0 | -1.1 | 2.3 | 0.24 |
| 9 | 32.4 | 7.1 | 5.1 | 38.0 | 8.1 | 3 | 0 | 5.3 | 2.3 | 1.14 |
| 10 | 23.3 | 0.1 | 0.1 | 23.3 | 0.1 | 3 | 0 | -3.8 | 2.3 | 0.81 |
| 11 | 26.4 | 1.0 | 0.8 | 25.9 | 0.9 | 3 | 0 | -0.7 | 2.3 | 0.15 |
| 12 | 27.0 | 0.4 | 0.3 | 27.9 | 1.0 | 3 | 0 | -0.1 | 2.3 | 0.02 |
| 13 | 29.0 | 0.0 | 0.0 | 29.3 | 0.3 | 3 | 0 | 1.9 | 2.3 | 0.41 |
| 14 | 28.0 | 0.0 | 0.0 | 28.0 | 0.0 | 3 | 0 | 0.9 | 2.3 | 0.19 |
| 15 | 26.6 | 0.6 | 0.4 | 27.0 | 0.6 | 3 | 0 | -0.5 | 2.3 | 0.10 |
| 16 | 29.0 | 0.0 | 0.0 | 29.0 | 0.0 | 3 | 0 | 1.9 | 2.3 | 0.41 |
| 17 | 28.0 | 1.5 | 1.1 | 28.0 | 0.6 | 3 | 0 | 0.9 | 2.3 | 0.19 |
| 18 | 24.0 | 3.0 | 2.1 | 24.3 | 1.5 | 3 | 0 | -3.1 | 2.3 | 0.66 |
| 19 | 28.2 | 1.5 | 1.1 | 28.1 | 0.6 | 3 | 0 | 1.1 | 2.3 | 0.23 |
| 20 | 26.9 | 0.0 | 0.0 | 27.4 | 0.5 | 3 | 0 | -0.2 | 2.3 | 0.04 |
| 21 | 30.0 | 0.0 | 0.0 | 29.7 | 0.3 | 3 | 0 | 2.9 | 2.3 | 0.62 |
| 22 | 30.0 | 0.0 | 0.0 | 30.0 | 0.0 | 3 | 0 | 2.9 | 2.3 | 0.62 |
| 23 | 26.4 | 0.7 | 0.5 | 26.1 | 0.6 | 3 | 0 | -0.7 | 2.3 | 0.15 |
| 24 | 25.0 | 0.0 | 0.0 | 26.0 | 1.0 | 3 | 0 | -2.1 | 2.3 | 0.45 |
| 25 | 27.0 | 0.0 | 0.0 | 27.3 | 0.3 | 3 | 0 | -0.1 | 2.3 | 0.02 |
| 26 | 23.3 | 0.4 | 0.3 | 23.2 | 0.3 | 3 | 0 | -3.8 | 2.3 | 0.81 |
| 27 | 28.1 | 0.1 | 0.0 | 27.9 | 0.3 | 3 | 0 | 1.0 | 2.3 | 0.22 |
| 28 | 19.0 | 3.0 | 2.1 | 18.3 | 1.8 | 3 | 0 | -8.1 | 2.3 | 1.74 |
| 29 | 25.8 | 0.3 | 0.2 | 25.6 | 0.3 | 3 | 0 | -1.3 | 2.3 | 0.28 |
| 30 | 28.8 | 1.0 | 0.8 | 28.1 | 1.0 | 3 | 0 | 1.7 | 2.3 | 0.36 |
| 31 | 34.0 | 4.4 | 3.2 | 33.7 | 2.0 | 3 | 0 | 6.9 | 2.3 | 1.48 |
| 32 | 27.9 | 0.3 | 0.2 | 27.9 | 0.1 | 3 | 0 | 0.8 | 2.3 | 0.17 |
| 33 | 27.0 | 0.0 | 0.0 | 26.7 | 0.3 | 3 | 0 | -0.1 | 2.3 | 0.02 |
| 34 | 29.4 | 2.1 | 1.5 | 27.7 | 2.4 | 3 | 0 | 2.3 | 2.3 | 0.49 |
| 35 | 25.0 | 1.5 | 1.1 | 25.0 | 0.6 | 3 | 0 | -2.1 | 2.3 | 0.45 |
| 36 | 26.0 | 0.3 | 0.2 | 25.9 | 0.2 | 3 | 0 | -1.1 | 2.3 | 0.24 |
| 37 | 27.1 | 0.1 | 0.1 | 26.3 | 0.8 | 3 | 0 | 0.0 | 2.3 | 0.01 |
| 38 | 28.0 | 0.0 | 0.0 | 27.7 | 0.3 | 3 | 0 | 0.9 | 2.3 | 0.19 |
| 39 | 26.2 | 0.4 | 0.3 | 26.1 | 0.3 | 3 | 0 | -0.9 | 2.3 | 0.19 |
| 40 | 29.0 | 1.5 | 1.1 | 29.0 | 0.6 | 3 | 0 | 1.9 | 2.3 | 0.41 |
| 41 | 25.4 | 0.1 | 0.1 | 25.3 | 0.2 | 3 | 0 | -1.7 | 2.3 | 0.36 |
| 42 | 28.2 | 1.3 | 1.0 | 28.5 | 0.8 | 3 | 0 | 1.1 | 2.3 | 0.24 |
| 43 | 25.1 | 0.7 | 0.5 | 24.7 | 0.6 | 3 | 0 | -2.0 | 2.3 | 0.44 |
| 44 | 29.0 | 1.5 | 1.1 | 29.3 | 0.9 | 3 | 0 | 1.9 | 2.3 | 0.41 |
| **Methionine** | | | | | | | | | | |
| 45 | 27.1 | 0.3 | 0.2 | 27.1 | 0.1 | 3 | 0 | 0.0 | 2.3 | 0.00 |
| 46 | 27.6 | 0.0 | 0.0 | 27.9 | 0.3 | 3 | 0 | 0.5 | 2.3 | 0.11 |
| 47 | 27.0 | 1.5 | 1.1 | 26.7 | 0.9 | 3 | 0 | -0.1 | 2.3 | 0.02 |
| 48 | 29.7 | 0.1 | 0.1 | 28.0 | 1.7 | 3 | 0 | 2.6 | 2.3 | 0.56 |
| 49 | 31.4 | 0.6 | 0.4 | 30.1 | 1.5 | 3 | 0 | 4.3 | 2.3 | 0.92 |
| 50 | 30.0 | 1.5 | 1.1 | 30.0 | 0.6 | 3 | 0 | 2.9 | 2.3 | 0.62 |
| 51 | 30.0 | 0.0 | 0.0 | 30.3 | 0.3 | 3 | 0 | 2.9 | 2.3 | 0.62 |
| 52 | 27.0 | 0.0 | 0.0 | 28.0 | 1.0 | 3 | 0 | -0.1 | 2.3 | 0.02 |
| 53 | 35.3 | 3.4 | 2.5 | 35.2 | 1.4 | 3 | 0 | 8.2 | 2.3 | 1.76 |
| 54 | 28.9 | 0.2 | 0.1 | 28.8 | 0.2 | 3 | 0 | 1.8 | 2.3 | 0.38 |
| 55 | 27.0 | 0.0 | 0.0 | 27.3 | 0.3 | 3 | 0 | -0.1 | 2.3 | 0.02 |
| 56 | 25.4 | 1.5 | 1.1 | 25.4 | 0.6 | 3 | 0 | -1.7 | 2.3 | 0.36 |
| 57 | 28.4 | 0.1 | 0.1 | 28.5 | 0.2 | 3 | 0 | 1.3 | 2.3 | 0.28 |
| 58 | 29.5 | 1.1 | 0.8 | 29.8 | 0.6 | 3 | 0 | 2.4 | 2.3 | 0.52 |
| 59 | 27.0 | 0.0 | 0.0 | 26.3 | 0.7 | 3 | 0 | -0.1 | 2.3 | 0.02 |
| 60 | 29.0 | 1.5 | 1.1 | 29.0 | 0.6 | 3 | 0 | 1.9 | 2.3 | 0.41 |
| 61 | 92.6 | 0.7 | 0.5 | 91.7 | 1.1 | 3 | 0 | 65.5 | 2.3 | 14.04 |
| 62 | 26.0 | 1.5 | 1.1 | 26.3 | 0.9 | 3 | 0 | -1.1 | 2.3 | 0.24 |
| 63 | 26.9 | 0.3 | 0.2 | 26.8 | 0.2 | 3 | 0 | -0.2 | 2.3 | 0.04 |
| 64 | 26.0 | 0.0 | 0.0 | 25.7 | 0.3 | 3 | 0 | -1.1 | 2.3 | 0.24 |
| 65 | 29.2 | 0.3 | 0.2 | 29.4 | 0.3 | 3 | 0 | 2.1 | 2.3 | 0.45 |
| 66 | 31.1 | 0.3 | 0.2 | 30.5 | 0.7 | 3 | 0 | 4.0 | 2.3 | 0.87 |
| 67 | 25.9 | 0.0 | 0.0 | 24.8 | 1.1 | 3 | 0 | -1.2 | 2.3 | 0.26 |
| 68 | 23.6 | 1.3 | 0.9 | 24.0 | 0.9 | 3 | 0 | -3.6 | 2.3 | 0.76 |
| 69 | 29.7 | 1.2 | 0.8 | 29.7 | 0.5 | 3 | 0 | 2.6 | 2.3 | 0.55 |
| 70 | 25.0 | 0.0 | 0.0 | 25.3 | 0.3 | 3 | 0 | -2.1 | 2.3 | 0.45 |
| 71 | 25.0 | 0.0 | 0.0 | 25.3 | 0.3 | 3 | 0 | -2.1 | 2.3 | 0.45 |
| 72 | 26.0 | 0.0 | 0.0 | 26.3 | 0.3 | 3 | 0 | -1.1 | 2.3 | 0.24 |
| 73 | 28.0 | 0.0 | 0.0 | 28.3 | 0.3 | 3 | 0 | 0.9 | 2.3 | 0.19 |
| 74 | 29.7 | 0.0 | 0.0 | 30.0 | 0.3 | 3 | 0 | 2.6 | 2.3 | 0.56 |
| 75 | 32.8 | 0.9 | 0.6 | 32.5 | 0.6 | 3 | 0 | 5.7 | 2.3 | 1.22 |
| 76 | 29.9 | 0.1 | 0.1 | 29.7 | 0.3 | 3 | 0 | 2.8 | 2.3 | 0.60 |
| 77 | 27.0 | 0.0 | 0.0 | 26.0 | 1.0 | 3 | 0 | -0.1 | 2.3 | 0.02 |
| 78 | 27.0 | 0.0 | 0.0 | 26.7 | 0.3 | 3 | 0 | -0.1 | 2.3 | 0.02 |
| 79 | 25.0 | 0.0 | 0.0 | 25.0 | 0.0 | 3 | 0 | -2.1 | 2.3 | 0.45 |
| 80 | 17.0 | 0.0 | 0.0 | 16.7 | 0.3 | 3 | 0 | -10.1 | 2.3 | 2.16 |
| 81 | 30.0 | 0.3 | 0.2 | 29.8 | 0.3 | 3 | 0 | 2.9 | 2.3 | 0.62 |
| 83 | 27.6 | 0.9 | 0.6 | 27.4 | 0.5 | 3 | 0 | 0.5 | 2.3 | 0.11 |
| 84 | 31.6 | 1.2 | 0.8 | 31.3 | 0.8 | 3 | 0 | 4.5 | 2.3 | 0.97 |
| 85 | 25.0 | 0.0 | 0.0 | 26.0 | 1.0 | 3 | 0 | -2.1 | 2.3 | 0.45 |
| 86 | 28.0 | 0.4 | 0.3 | 28.7 | 0.9 | 3 | 0 | 0.9 | 2.3 | 0.19 |
| 87 | 26.0 | 0.0 | 0.0 | 26.3 | 0.3 | 3 | 0 | -1.1 | 2.3 | 0.24 |
| 88 | 28.0 | 1.5 | 1.1 | 27.7 | 0.9 | 3 | 0 | 0.9 | 2.3 | 0.19 |
| 89 | 27.0 | 1.5 | 1.1 | 26.7 | 0.9 | 3 | 0 | -0.1 | 2.3 | 0.02 |
| 90 | 28.7 | 0.1 | 0.1 | 28.6 | 0.2 | 3 | 0 | 1.6 | 2.3 | 0.34 |

**Ornithine**

| **Lab** | **Median** | **MAD** | **u (median)** | **Mean** | **u (mean)** | **n** | **Non-detects** | **DoE** | **u (DoE)** | **Score** |
| --- | --- | --- | --- | --- | --- | --- | --- | --- | --- | --- |
| 1 | 111.9 | 1.5 | 1.1 | 111.2 | 1.2 | 3 | 0 | 2.9 | 7.7 | 0.19 |
| 2 | 109.0 | 3.0 | 2.1 | 109.7 | 1.8 | 3 | 0 | 0.0 | 7.7 | 0.00 |
| 3 | 100.0 | 1.5 | 1.1 | 102.7 | 3.2 | 3 | 0 | -9.0 | 7.7 | 0.58 |
| 4 | 94.7 | 2.8 | 2.0 | 93.5 | 2.2 | 3 | 0 | -14.3 | 7.7 | 0.93 |
| 5 | 109.0 | 1.5 | 1.1 | 108.3 | 1.2 | 3 | 0 | 0.0 | 7.7 | 0.00 |
| 6 | 106.0 | 0.0 | 0.0 | 106.7 | 0.7 | 3 | 0 | -3.0 | 7.7 | 0.19 |
| 7 | 85.0 | 1.5 | 1.1 | 82.3 | 3.2 | 3 | 0 | -24.0 | 7.7 | 1.56 |
| 8 | 109.0 | 0.0 | 0.0 | 108.0 | 1.0 | 3 | 0 | 0.0 | 7.7 | 0.00 |
| 9 | 133.7 | 24.0 | 17.4 | 161.9 | 36.7 | 3 | 0 | 24.7 | 7.7 | 1.60 |
| 10 | 113.7 | 5.1 | 3.7 | 113.8 | 2.1 | 3 | 0 | 4.7 | 7.7 | 0.31 |
| 11 | 111.0 | 3.0 | 2.1 | 108.3 | 3.7 | 3 | 0 | 2.0 | 7.7 | 0.13 |
| 12 | 91.0 | 0.0 | 0.0 | 92.1 | 1.1 | 3 | 0 | -18.0 | 7.7 | 1.17 |
| 13 | 109.0 | 3.0 | 2.1 | 109.3 | 1.5 | 3 | 0 | 0.0 | 7.7 | 0.00 |
| 14 | 130.0 | 4.4 | 3.2 | 136.7 | 8.2 | 3 | 0 | 21.0 | 7.7 | 1.36 |
| 15 | 110.7 | 0.7 | 0.5 | 110.7 | 0.3 | 3 | 0 | 1.7 | 7.7 | 0.11 |
| 16 | 119.0 | 5.9 | 4.3 | 118.0 | 3.2 | 3 | 0 | 10.0 | 7.7 | 0.65 |
| 17 | 115.0 | 1.5 | 1.1 | 115.7 | 1.2 | 3 | 0 | 6.0 | 7.7 | 0.39 |
| 18 | 105.0 | 3.0 | 2.1 | 101.7 | 4.4 | 3 | 0 | -4.0 | 7.7 | 0.26 |
| 19 | 116.9 | 5.4 | 3.9 | 117.2 | 2.4 | 3 | 0 | 7.9 | 7.7 | 0.51 |
| 20 | 93.1 | 22.5 | 16.3 | 89.4 | 12.2 | 3 | 0 | -15.9 | 7.7 | 1.03 |
| 21 | 112.0 | 3.0 | 2.1 | 111.7 | 1.5 | 3 | 0 | 3.0 | 7.7 | 0.19 |
| 22 | 118.0 | 0.0 | 0.0 | 118.3 | 0.3 | 3 | 0 | 9.0 | 7.7 | 0.58 |
| 23 | 105.7 | 3.7 | 2.7 | 105.5 | 1.6 | 3 | 0 | -3.3 | 7.7 | 0.21 |
| 24 | 108.0 | 1.5 | 1.1 | 109.7 | 2.2 | 3 | 0 | -1.0 | 7.7 | 0.06 |
| 25 | 113.0 | 1.5 | 1.1 | 113.7 | 1.2 | 3 | 0 | 4.0 | 7.7 | 0.26 |
| 26 | 113.7 | 11.1 | 8.0 | 112.1 | 5.7 | 3 | 0 | 4.7 | 7.7 | 0.31 |
| 27 | 123.7 | 0.7 | 0.5 | 122.9 | 1.0 | 3 | 0 | 14.7 | 7.7 | 0.95 |
| 28 | 107.0 | 0.0 | 0.0 | 107.7 | 0.7 | 3 | 0 | -2.0 | 7.7 | 0.13 |
| 29 | 108.4 | 1.9 | 1.4 | 107.9 | 1.2 | 3 | 0 | -0.6 | 7.7 | 0.04 |
| 30 | 110.2 | 4.2 | 3.0 | 112.4 | 3.7 | 3 | 0 | 1.2 | 7.7 | 0.08 |
| 31 | 107.0 | 5.9 | 4.3 | 102.0 | 7.1 | 3 | 0 | -2.0 | 7.7 | 0.13 |
| 32 | 116.2 | 0.1 | 0.1 | 115.2 | 1.1 | 3 | 0 | 7.2 | 7.7 | 0.47 |
| 33 | 105.0 | 3.0 | 2.1 | 105.3 | 1.5 | 3 | 0 | -4.0 | 7.7 | 0.26 |
| 34 | 88.9 | 13.0 | 9.4 | 90.0 | 6.1 | 3 | 0 | -20.1 | 7.7 | 1.31 |
| 35 | 113.0 | 0.0 | 0.0 | 111.3 | 1.7 | 3 | 0 | 4.0 | 7.7 | 0.26 |
| 36 | 112.3 | 1.8 | 1.3 | 112.7 | 1.0 | 3 | 0 | 3.3 | 7.7 | 0.22 |
| 37 | 103.4 | 0.4 | 0.3 | 103.2 | 0.4 | 3 | 0 | -5.6 | 7.7 | 0.36 |
| 38 | 111.0 | 3.0 | 2.1 | 112.3 | 2.4 | 3 | 0 | 2.0 | 7.7 | 0.13 |
| 39 | 100.0 | 1.5 | 1.1 | 99.8 | 0.8 | 3 | 0 | -9.0 | 7.7 | 0.58 |
| 40 | 117.0 | 2.2 | 1.6 | 118.0 | 1.8 | 3 | 0 | 8.0 | 7.7 | 0.52 |
| 41 | 100.7 | 1.5 | 1.1 | 99.9 | 1.3 | 3 | 0 | -8.3 | 7.7 | 0.54 |
| 42 | 109.7 | 0.1 | 0.1 | 112.0 | 2.3 | 3 | 0 | 0.7 | 7.7 | 0.05 |
| 43 | 101.8 | 2.2 | 1.6 | 101.5 | 1.1 | 3 | 0 | -7.2 | 7.7 | 0.47 |
| 44 | 113.0 | 1.5 | 1.1 | 113.0 | 0.6 | 3 | 0 | 4.0 | 7.7 | 0.26 |
| **Ornithine** | | | | | | | | | | |
| 45 | 120.7 | 4.0 | 2.9 | 117.8 | 4.4 | 3 | 0 | 11.7 | 7.7 | 0.76 |
| 46 | 109.0 | 5.0 | 3.6 | 107.1 | 3.8 | 3 | 0 | 0.0 | 7.7 | 0.00 |
| 47 | 109.0 | 7.4 | 5.4 | 110.0 | 3.8 | 3 | 0 | 0.0 | 7.7 | 0.00 |
| 48 | 105.2 | 0.0 | 0.0 | 106.5 | 1.3 | 3 | 0 | -3.8 | 7.7 | 0.25 |
| 49 | 123.0 | 14.8 | 10.7 | 128.0 | 10.4 | 3 | 0 | 14.0 | 7.7 | 0.91 |
| 50 | 134.0 | 1.5 | 1.1 | 133.0 | 1.5 | 3 | 0 | 25.0 | 7.7 | 1.62 |
| 51 | 112.0 | 0.0 | 0.0 | 113.0 | 1.0 | 3 | 0 | 3.0 | 7.7 | 0.19 |
| 52 | 88.0 | 0.0 | 0.0 | 91.7 | 3.7 | 3 | 0 | -21.0 | 7.7 | 1.36 |
| 53 | 96.9 | 11.7 | 8.5 | 96.9 | 4.6 | 3 | 0 | -12.1 | 7.7 | 0.79 |
| 54 | 101.6 | 3.0 | 2.1 | 100.7 | 1.9 | 3 | 0 | -7.4 | 7.7 | 0.48 |
| 55 | 108.0 | 0.0 | 0.0 | 107.7 | 0.3 | 3 | 0 | -1.0 | 7.7 | 0.06 |
| 56 | 102.2 | 4.4 | 3.2 | 101.8 | 2.1 | 3 | 0 | -6.8 | 7.7 | 0.44 |
| 57 | 108.3 | 0.3 | 0.2 | 108.6 | 0.4 | 3 | 0 | -0.7 | 7.7 | 0.05 |
| 58 | 111.3 | 0.1 | 0.1 | 108.7 | 2.6 | 3 | 0 | 2.3 | 7.7 | 0.15 |
| 59 | 108.0 | 0.0 | 0.0 | 106.3 | 1.7 | 3 | 0 | -1.0 | 7.7 | 0.06 |
| 60 | 110.0 | 10.4 | 7.5 | 111.0 | 4.9 | 3 | 0 | 1.0 | 7.7 | 0.06 |
| 61 | 0.0 | 0.0 | 0.0 | 0.0 | 0.0 | 3 | 1 | -109.0 | 7.7 | 7.08 |
| 62 | 103.0 | 1.5 | 1.1 | 104.7 | 2.2 | 3 | 0 | -6.0 | 7.7 | 0.39 |
| 63 | 108.1 | 2.1 | 1.5 | 108.4 | 1.1 | 3 | 0 | -0.9 | 7.7 | 0.06 |
| 64 | 96.0 | 3.0 | 2.1 | 98.0 | 3.1 | 3 | 0 | -13.0 | 7.7 | 0.84 |
| 65 | 110.3 | 1.1 | 0.8 | 120.5 | 10.5 | 3 | 0 | 1.3 | 7.7 | 0.09 |
| 66 | 129.0 | 3.0 | 2.1 | 129.6 | 1.7 | 3 | 0 | 20.0 | 7.7 | 1.30 |
| 67 | 113.0 | 1.5 | 1.1 | 109.3 | 4.2 | 3 | 0 | 4.0 | 7.7 | 0.26 |
| 68 | 114.9 | 3.3 | 2.4 | 117.8 | 4.1 | 3 | 0 | 5.9 | 7.7 | 0.38 |
| 69 | 116.8 | 6.8 | 4.9 | 117.8 | 3.5 | 3 | 0 | 7.8 | 7.7 | 0.51 |
| 70 | 106.0 | 5.9 | 4.3 | 105.3 | 2.9 | 3 | 0 | -3.0 | 7.7 | 0.19 |
| 71 | 90.0 | 0.0 | 0.0 | 89.0 | 1.0 | 3 | 0 | -19.0 | 7.7 | 1.23 |
| 72 | 106.0 | 1.5 | 1.1 | 105.7 | 0.9 | 3 | 0 | -3.0 | 7.7 | 0.19 |
| 73 | 103.0 | 0.0 | 0.0 | 102.7 | 0.3 | 3 | 0 | -6.0 | 7.7 | 0.39 |
| 74 | 118.1 | 4.4 | 3.2 | 119.3 | 2.8 | 3 | 0 | 9.1 | 7.7 | 0.59 |
| 75 | 117.5 | 0.6 | 0.4 | 116.7 | 1.0 | 3 | 0 | 8.5 | 7.7 | 0.55 |
| 76 | 116.8 | 1.0 | 0.8 | 116.3 | 0.9 | 3 | 0 | 7.8 | 7.7 | 0.51 |
| 77 | 106.0 | 7.4 | 5.4 | 106.3 | 3.2 | 3 | 0 | -3.0 | 7.7 | 0.19 |
| 78 | 106.0 | 4.4 | 3.2 | 107.0 | 2.6 | 3 | 0 | -3.0 | 7.7 | 0.19 |
| 79 | 110.0 | 0.0 | 0.0 | 110.0 | 0.0 | 3 | 0 | 1.0 | 7.7 | 0.06 |
| 80 | 66.0 | 5.9 | 4.3 | 65.7 | 2.6 | 3 | 0 | -43.0 | 7.7 | 2.79 |
| 81 | 115.1 | 1.2 | 0.9 | 115.2 | 0.5 | 3 | 0 | 6.1 | 7.7 | 0.40 |
| 83 | 45.3 | 0.4 | 0.3 | 43.5 | 2.0 | 3 | 0 | -63.7 | 7.7 | 4.14 |
| 84 | 115.3 | 7.6 | 5.5 | 114.9 | 3.3 | 3 | 0 | 6.3 | 7.7 | 0.41 |
| 85 | 98.0 | 11.9 | 8.6 | 97.7 | 4.9 | 3 | 0 | -11.0 | 7.7 | 0.71 |
| 86 | 115.3 | 7.0 | 5.0 | 115.1 | 2.9 | 3 | 0 | 6.3 | 7.7 | 0.41 |
| 87 | 106.0 | 1.5 | 1.1 | 106.7 | 1.2 | 3 | 0 | -3.0 | 7.7 | 0.19 |
| 88 | 103.4 | 25.5 | 18.5 | 106.3 | 12.5 | 3 | 0 | -5.6 | 7.7 | 0.36 |
| 89 | 115.0 | 0.0 | 0.0 | 111.0 | 4.0 | 3 | 0 | 6.0 | 7.7 | 0.39 |
| 90 | 132.0 | 5.3 | 3.9 | 131.4 | 2.6 | 3 | 0 | 23.0 | 7.7 | 1.49 |

**Phenylalanine**

| **Lab** | **Median** | **MAD** | **u (median)** | **Mean** | **u (mean)** | **n** | **Non-detects** | **DoE** | **u (DoE)** | **Score** |
| --- | --- | --- | --- | --- | --- | --- | --- | --- | --- | --- |
| 1 | 363.5 | 5.0 | 3.6 | 361.5 | 3.8 | 3 | 0 | 4.5 | 17.8 | 0.13 |
| 2 | 351.0 | 5.9 | 4.3 | 353.0 | 4.2 | 3 | 0 | -8.0 | 17.8 | 0.22 |
| 3 | 352.0 | 4.4 | 3.2 | 358.3 | 7.9 | 3 | 0 | -7.0 | 17.8 | 0.20 |
| 4 | 350.0 | 0.0 | 0.0 | 348.7 | 1.3 | 3 | 0 | -9.0 | 17.8 | 0.25 |
| 5 | 355.0 | 4.4 | 3.2 | 350.3 | 6.2 | 3 | 0 | -4.0 | 17.8 | 0.11 |
| 6 | 350.0 | 3.0 | 2.1 | 350.7 | 1.8 | 3 | 0 | -9.0 | 17.8 | 0.25 |
| 7 | 346.0 | 5.9 | 4.3 | 356.3 | 12.4 | 3 | 0 | -13.0 | 17.8 | 0.36 |
| 8 | 355.0 | 3.0 | 2.1 | 353.0 | 3.1 | 3 | 0 | -4.0 | 17.8 | 0.11 |
| 9 | 388.8 | 47.1 | 34.1 | 485.3 | 112.7 | 3 | 0 | 29.8 | 17.8 | 0.84 |
| 10 | 361.9 | 5.0 | 3.6 | 359.5 | 4.2 | 3 | 0 | 2.9 | 17.8 | 0.08 |
| 11 | 347.0 | 14.8 | 10.7 | 349.3 | 7.9 | 3 | 0 | -12.0 | 17.8 | 0.34 |
| 12 | 375.0 | 14.3 | 10.3 | 379.5 | 9.7 | 3 | 0 | 16.0 | 17.8 | 0.45 |
| 13 | 356.0 | 0.0 | 0.0 | 358.0 | 2.0 | 3 | 0 | -3.0 | 17.8 | 0.08 |
| 14 | 367.0 | 0.0 | 0.0 | 368.0 | 1.0 | 3 | 0 | 8.0 | 17.8 | 0.22 |
| 15 | 348.2 | 2.0 | 1.4 | 346.9 | 2.1 | 3 | 0 | -10.8 | 17.8 | 0.30 |
| 16 | 372.0 | 1.5 | 1.1 | 372.3 | 0.9 | 3 | 0 | 13.0 | 17.8 | 0.36 |
| 17 | 355.0 | 3.0 | 2.1 | 353.0 | 3.1 | 3 | 0 | -4.0 | 17.8 | 0.11 |
| 18 | 374.0 | 3.0 | 2.1 | 377.3 | 4.4 | 3 | 0 | 15.0 | 17.8 | 0.42 |
| 19 | 376.1 | 14.6 | 10.6 | 376.3 | 5.9 | 3 | 0 | 17.1 | 17.8 | 0.48 |
| 20 | 360.0 | 13.5 | 9.8 | 362.6 | 7.6 | 3 | 0 | 1.0 | 17.8 | 0.03 |
| 21 | 397.0 | 3.0 | 2.1 | 397.0 | 1.2 | 3 | 0 | 38.0 | 17.8 | 1.06 |
| 22 | 378.0 | 0.0 | 0.0 | 378.3 | 0.3 | 3 | 0 | 19.0 | 17.8 | 0.53 |
| 23 | 337.0 | 5.8 | 4.2 | 338.1 | 3.3 | 3 | 0 | -22.0 | 17.8 | 0.62 |
| 24 | 343.0 | 0.0 | 0.0 | 351.0 | 8.0 | 3 | 0 | -16.0 | 17.8 | 0.45 |
| 25 | 368.0 | 3.0 | 2.1 | 370.3 | 3.4 | 3 | 0 | 9.0 | 17.8 | 0.25 |
| 26 | 314.1 | 3.4 | 2.5 | 315.5 | 2.6 | 3 | 0 | -44.9 | 17.8 | 1.26 |
| 27 | 383.0 | 0.1 | 0.1 | 379.2 | 3.9 | 3 | 0 | 24.0 | 17.8 | 0.67 |
| 28 | 335.0 | 3.0 | 2.1 | 338.3 | 4.4 | 3 | 0 | -24.0 | 17.8 | 0.67 |
| 29 | 339.6 | 8.3 | 6.0 | 344.8 | 8.1 | 3 | 0 | -19.4 | 17.8 | 0.54 |
| 30 | 370.2 | 28.5 | 20.6 | 366.8 | 14.1 | 3 | 0 | 11.2 | 17.8 | 0.31 |
| 31 | 373.0 | 5.9 | 4.3 | 398.0 | 27.0 | 3 | 0 | 14.0 | 17.8 | 0.39 |
| 32 | 404.0 | 4.2 | 3.0 | 403.5 | 2.1 | 3 | 0 | 45.0 | 17.8 | 1.26 |
| 33 | 360.0 | 4.4 | 3.2 | 358.0 | 3.6 | 3 | 0 | 1.0 | 17.8 | 0.03 |
| 34 | 397.0 | 3.0 | 2.1 | 414.3 | 18.3 | 3 | 0 | 38.0 | 17.8 | 1.06 |
| 35 | 363.0 | 8.9 | 6.4 | 362.3 | 4.1 | 3 | 0 | 4.0 | 17.8 | 0.11 |
| 36 | 339.1 | 6.0 | 4.3 | 339.6 | 2.8 | 3 | 0 | -19.9 | 17.8 | 0.56 |
| 37 | 346.4 | 0.3 | 0.2 | 344.8 | 1.7 | 3 | 0 | -12.6 | 17.8 | 0.35 |
| 38 | 370.0 | 5.9 | 4.3 | 369.3 | 2.9 | 3 | 0 | 11.0 | 17.8 | 0.31 |
| 39 | 265.0 | 17.8 | 12.9 | 265.3 | 7.2 | 3 | 0 | -94.0 | 17.8 | 2.63 |
| 40 | 361.5 | 8.2 | 5.9 | 363.0 | 4.5 | 3 | 0 | 2.5 | 17.8 | 0.07 |
| 41 | 354.1 | 1.9 | 1.4 | 358.7 | 5.3 | 3 | 0 | -4.9 | 17.8 | 0.14 |
| 42 | 372.8 | 11.4 | 8.3 | 371.3 | 5.8 | 3 | 0 | 13.8 | 17.8 | 0.39 |
| 43 | 309.1 | 2.7 | 2.0 | 311.1 | 3.0 | 3 | 0 | -49.9 | 17.8 | 1.40 |
| 44 | 357.0 | 8.9 | 6.4 | 354.7 | 5.6 | 3 | 0 | -2.0 | 17.8 | 0.06 |
| **Phenylalanine** | | | | | | | | | | |
| 45 | 367.0 | 1.3 | 1.0 | 367.9 | 1.4 | 3 | 0 | 8.0 | 17.8 | 0.22 |
| 46 | 356.2 | 6.1 | 4.4 | 359.1 | 5.1 | 3 | 0 | -2.8 | 17.8 | 0.08 |
| 47 | 383.0 | 1.5 | 1.1 | 383.7 | 1.2 | 3 | 0 | 24.0 | 17.8 | 0.67 |
| 48 | 350.5 | 2.2 | 1.6 | 346.3 | 4.9 | 3 | 0 | -8.5 | 17.8 | 0.24 |
| 49 | 376.0 | 3.0 | 2.1 | 387.0 | 12.0 | 3 | 0 | 17.0 | 17.8 | 0.48 |
| 50 | 320.0 | 5.9 | 4.3 | 314.7 | 7.4 | 3 | 0 | -39.0 | 17.8 | 1.09 |
| 51 | 366.0 | 4.4 | 3.2 | 365.7 | 2.0 | 3 | 0 | 7.0 | 17.8 | 0.20 |
| 52 | 342.0 | 17.8 | 12.9 | 348.7 | 13.1 | 3 | 0 | -17.0 | 17.8 | 0.48 |
| 53 | 351.6 | 5.8 | 4.2 | 344.7 | 8.9 | 3 | 0 | -7.4 | 17.8 | 0.21 |
| 54 | 330.8 | 1.4 | 1.0 | 330.8 | 0.6 | 3 | 0 | -28.2 | 17.8 | 0.79 |
| 55 | 360.0 | 1.5 | 1.1 | 359.7 | 0.9 | 3 | 0 | 1.0 | 17.8 | 0.03 |
| 56 | 339.2 | 11.1 | 8.0 | 338.3 | 5.1 | 3 | 0 | -19.8 | 17.8 | 0.55 |
| 57 | 375.4 | 10.4 | 7.5 | 375.8 | 4.4 | 3 | 0 | 16.4 | 17.8 | 0.46 |
| 58 | 366.6 | 2.0 | 1.5 | 364.1 | 3.2 | 3 | 0 | 7.6 | 17.8 | 0.21 |
| 59 | 349.0 | 3.0 | 2.1 | 345.0 | 5.0 | 3 | 0 | -10.0 | 17.8 | 0.28 |
| 60 | 366.0 | 4.4 | 3.2 | 366.0 | 1.7 | 3 | 0 | 7.0 | 17.8 | 0.20 |
| 61 | 359.6 | 5.5 | 4.0 | 359.9 | 2.4 | 3 | 0 | 0.6 | 17.8 | 0.02 |
| 62 | 327.0 | 5.9 | 4.3 | 332.7 | 7.8 | 3 | 0 | -32.0 | 17.8 | 0.90 |
| 63 | 358.1 | 1.2 | 0.9 | 360.0 | 2.3 | 3 | 0 | -0.9 | 17.8 | 0.03 |
| 64 | 348.0 | 0.0 | 0.0 | 342.3 | 5.7 | 3 | 0 | -11.0 | 17.8 | 0.31 |
| 65 | 374.4 | 2.1 | 1.5 | 377.0 | 3.3 | 3 | 0 | 15.4 | 17.8 | 0.43 |
| 66 | 389.0 | 7.4 | 5.4 | 390.3 | 4.1 | 3 | 0 | 30.0 | 17.8 | 0.84 |
| 67 | 347.0 | 0.0 | 0.0 | 332.3 | 14.7 | 3 | 0 | -12.0 | 17.8 | 0.34 |
| 68 | 389.3 | 3.5 | 2.5 | 400.5 | 12.4 | 3 | 0 | 30.3 | 17.8 | 0.85 |
| 69 | 391.7 | 6.4 | 4.7 | 392.7 | 3.4 | 3 | 0 | 32.7 | 17.8 | 0.92 |
| 70 | 347.0 | 11.9 | 8.6 | 342.7 | 8.6 | 3 | 0 | -12.0 | 17.8 | 0.34 |
| 71 | 324.0 | 13.3 | 9.7 | 316.0 | 12.8 | 3 | 0 | -35.0 | 17.8 | 0.98 |
| 72 | 359.0 | 3.0 | 2.1 | 359.0 | 1.2 | 3 | 0 | 0.0 | 17.8 | 0.00 |
| 73 | 344.0 | 1.5 | 1.1 | 342.0 | 2.5 | 3 | 0 | -15.0 | 17.8 | 0.42 |
| 74 | 406.7 | 13.9 | 10.1 | 406.5 | 5.6 | 3 | 0 | 47.7 | 17.8 | 1.34 |
| 75 | 376.5 | 1.9 | 1.4 | 372.8 | 4.3 | 3 | 0 | 17.5 | 17.8 | 0.49 |
| 76 | 361.8 | 30.1 | 21.8 | 371.0 | 20.2 | 3 | 0 | 2.8 | 17.8 | 0.08 |
| 77 | 345.0 | 20.8 | 15.0 | 347.0 | 9.9 | 3 | 0 | -14.0 | 17.8 | 0.39 |
| 78 | 354.0 | 17.8 | 12.9 | 354.7 | 7.5 | 3 | 0 | -5.0 | 17.8 | 0.14 |
| 79 | 343.0 | 1.5 | 1.1 | 343.0 | 0.6 | 3 | 0 | -16.0 | 17.8 | 0.45 |
| 80 | 234.0 | 3.0 | 2.1 | 231.7 | 3.4 | 3 | 0 | -125.0 | 17.8 | 3.50 |
| 81 | 373.8 | 8.2 | 5.9 | 373.1 | 3.8 | 3 | 0 | 14.8 | 17.8 | 0.41 |
| 83 | 367.3 | 5.2 | 3.8 | 366.9 | 2.4 | 3 | 0 | 8.3 | 17.8 | 0.23 |
| 84 | 331.2 | 11.4 | 8.2 | 332.4 | 5.5 | 3 | 0 | -27.8 | 17.8 | 0.78 |
| 85 | 373.0 | 11.9 | 8.6 | 379.3 | 10.6 | 3 | 0 | 14.0 | 17.8 | 0.39 |
| 86 | 370.0 | 3.7 | 2.7 | 372.5 | 3.8 | 3 | 0 | 11.0 | 17.8 | 0.31 |
| 87 | 354.0 | 5.9 | 4.3 | 355.0 | 3.2 | 3 | 0 | -5.0 | 17.8 | 0.14 |
| 88 | 353.5 | 8.3 | 6.0 | 364.1 | 13.6 | 3 | 0 | -5.5 | 17.8 | 0.15 |
| 89 | 376.0 | 5.9 | 4.3 | 368.3 | 9.7 | 3 | 0 | 17.0 | 17.8 | 0.48 |
| 90 | 385.7 | 1.6 | 1.2 | 381.8 | 4.5 | 3 | 0 | 26.7 | 17.8 | 0.75 |

**Proline**

| **Lab** | **Median** | **MAD** | **u (median)** | **Mean** | **u (mean)** | **n** | **Non-detects** | **DoE** | **u (DoE)** | **Score** |
| --- | --- | --- | --- | --- | --- | --- | --- | --- | --- | --- |
| 1 | 249 | 3 | 2 | 245 | 5 | 3 | 0 | 21.5 | 15.1 | 0.71 |
| 2 | 208 | 0 | 0 | 212 | 4 | 3 | 0 | -19.5 | 15.1 | 0.65 |
| 3 | 200 | 10 | 8 | 209 | 13 | 3 | 0 | -27.5 | 15.1 | 0.91 |
| 4 | 227 | 0 | 0 | 224 | 3 | 3 | 0 | -0.5 | 15.1 | 0.02 |
| 5 | 267 | 19 | 14 | 267 | 8 | 3 | 0 | 39.5 | 15.1 | 1.31 |
| 6 | 192 | 6 | 4 | 199 | 9 | 3 | 0 | -35.5 | 15.1 | 1.18 |
| 7 | 215 | 12 | 9 | 214 | 6 | 3 | 0 | -12.5 | 15.1 | 0.41 |
| 8 | 233 | 0 | 0 | 234 | 1 | 3 | 0 | 5.5 | 15.1 | 0.18 |
| 9 | 266 | 57 | 42 | 320 | 74 | 3 | 0 | 38.8 | 15.1 | 1.28 |
| 10 | 231 | 2 | 1 | 210 | 21 | 3 | 0 | 3.1 | 15.1 | 0.10 |
| 11 | 222 | 15 | 11 | 213 | 15 | 3 | 0 | -5.5 | 15.1 | 0.18 |
| 12 | 218 | 7 | 5 | 225 | 10 | 3 | 0 | -10.0 | 15.1 | 0.33 |
| 13 | 238 | 3 | 2 | 242 | 5 | 3 | 0 | 10.5 | 15.1 | 0.35 |
| 14 | 228 | 4 | 3 | 230 | 3 | 3 | 0 | 0.5 | 15.1 | 0.02 |
| 15 | 257 | 22 | 16 | 261 | 12 | 3 | 0 | 29.5 | 15.1 | 0.98 |
| 16 | 230 | 1 | 1 | 231 | 1 | 3 | 0 | 2.5 | 15.1 | 0.08 |
| 17 | 238 | 19 | 14 | 243 | 12 | 3 | 0 | 10.5 | 15.1 | 0.35 |
| 18 | 224 | 7 | 5 | 225 | 4 | 3 | 0 | -3.5 | 15.1 | 0.12 |
| 19 | 221 | 5 | 4 | 218 | 5 | 3 | 0 | -6.4 | 15.1 | 0.21 |
| 20 | 216 | 12 | 9 | 219 | 8 | 3 | 0 | -11.6 | 15.1 | 0.38 |
| 21 | 239 | 0 | 0 | 239 | 0 | 3 | 0 | 11.5 | 15.1 | 0.38 |
| 22 | 244 | 0 | 0 | 241 | 3 | 3 | 0 | 16.5 | 15.1 | 0.55 |
| 23 | 241 | 16 | 11 | 232 | 15 | 3 | 0 | 13.5 | 15.1 | 0.45 |
| 24 | 216 | 1 | 1 | 222 | 6 | 3 | 0 | -11.5 | 15.1 | 0.38 |
| 25 | 243 | 3 | 2 | 242 | 2 | 3 | 0 | 15.5 | 15.1 | 0.51 |
| 26 | 208 | 7 | 5 | 209 | 4 | 3 | 0 | -19.4 | 15.1 | 0.64 |
| 27 | 232 | 0 | 0 | 239 | 7 | 3 | 0 | 4.6 | 15.1 | 0.15 |
| 28 | 163 | 6 | 4 | 161 | 4 | 3 | 0 | -64.5 | 15.1 | 2.14 |
| 29 | 209 | 4 | 3 | 213 | 5 | 3 | 0 | -18.2 | 15.1 | 0.60 |
| 30 | 240 | 17 | 12 | 236 | 10 | 3 | 0 | 12.3 | 15.1 | 0.41 |
| 31 | 224 | 10 | 8 | 225 | 5 | 3 | 0 | -3.5 | 15.1 | 0.12 |
| 32 | 268 | 7 | 5 | 267 | 3 | 3 | 0 | 40.1 | 15.1 | 1.33 |
| 33 | 227 | 3 | 2 | 225 | 3 | 3 | 0 | -0.5 | 15.1 | 0.02 |
| 34 | 240 | 25 | 18 | 241 | 10 | 3 | 0 | 12.5 | 15.1 | 0.41 |
| 35 | 219 | 1 | 1 | 220 | 2 | 3 | 0 | -8.5 | 15.1 | 0.28 |
| 36 | 233 | 3 | 2 | 233 | 2 | 3 | 0 | 5.8 | 15.1 | 0.19 |
| 37 | 237 | 2 | 1 | 237 | 1 | 3 | 0 | 9.4 | 15.1 | 0.31 |
| 38 | 221 | 4 | 3 | 221 | 2 | 3 | 0 | -6.5 | 15.1 | 0.22 |
| 39 | 192 | 7 | 5 | 197 | 7 | 3 | 0 | -35.5 | 15.1 | 1.18 |
| 40 | 232 | 8 | 6 | 236 | 7 | 3 | 0 | 4.0 | 15.1 | 0.13 |
| 41 | 207 | 5 | 4 | 209 | 4 | 3 | 0 | -20.5 | 15.1 | 0.68 |
| 42 | 238 | 11 | 8 | 238 | 4 | 3 | 0 | 10.4 | 15.1 | 0.34 |
| 43 | 221 | 28 | 21 | 251 | 40 | 3 | 0 | -6.1 | 15.1 | 0.20 |
| 45 | 232 | 1 | 1 | 229 | 4 | 3 | 0 | 4.8 | 15.1 | 0.16 |
| **Proline** | | | | | | | | | | |
| 46 | 234 | 1 | 1 | 230 | 5 | 3 | 0 | 6.5 | 15.1 | 0.22 |
| 47 | 225 | 6 | 4 | 222 | 5 | 3 | 0 | -2.5 | 15.1 | 0.08 |
| 48 | 184 | 14 | 10 | 191 | 12 | 3 | 0 | -43.2 | 15.1 | 1.43 |
| 49 | 251 | 21 | 15 | 247 | 11 | 3 | 0 | 23.5 | 15.1 | 0.78 |
| 50 | 203 | 9 | 6 | 206 | 6 | 3 | 0 | -24.5 | 15.1 | 0.81 |
| 51 | 242 | 3 | 2 | 242 | 1 | 3 | 0 | 14.5 | 15.1 | 0.48 |
| 52 | 225 | 0 | 0 | 227 | 2 | 3 | 0 | -2.5 | 15.1 | 0.08 |
| 53 | 236 | 1 | 1 | 243 | 8 | 3 | 0 | 8.1 | 15.1 | 0.27 |
| 54 | 286 | 20 | 14 | 287 | 9 | 3 | 0 | 58.7 | 15.1 | 1.94 |
| 55 | 233 | 0 | 0 | 234 | 1 | 3 | 0 | 5.5 | 15.1 | 0.18 |
| 56 | 211 | 6 | 4 | 211 | 2 | 3 | 0 | -16.7 | 15.1 | 0.55 |
| 57 | 222 | 9 | 6 | 223 | 4 | 3 | 0 | -5.2 | 15.1 | 0.17 |
| 58 | 235 | 1 | 1 | 232 | 3 | 3 | 0 | 7.1 | 15.1 | 0.24 |
| 59 | 237 | 3 | 2 | 233 | 5 | 3 | 0 | 9.5 | 15.1 | 0.31 |
| 60 | 227 | 0 | 0 | 225 | 2 | 3 | 0 | -0.5 | 15.1 | 0.02 |
| 61 | 0 | 0 | 0 | 0 | 0 | 3 | 1 | -227.5 | 15.1 | 7.53 |
| 62 | 242 | 21 | 15 | 236 | 14 | 3 | 0 | 14.5 | 15.1 | 0.48 |
| 63 | 222 | 8 | 6 | 220 | 5 | 3 | 0 | -6.0 | 15.1 | 0.20 |
| 64 | 212 | 0 | 0 | 208 | 4 | 3 | 0 | -15.5 | 15.1 | 0.51 |
| 65 | 203 | 1 | 1 | 211 | 8 | 3 | 0 | -24.3 | 15.1 | 0.80 |
| 66 | 227 | 10 | 8 | 228 | 5 | 3 | 0 | -0.5 | 15.1 | 0.02 |
| 67 | 226 | 12 | 9 | 222 | 9 | 3 | 0 | -1.5 | 15.1 | 0.05 |
| 68 | 233 | 1 | 1 | 241 | 8 | 3 | 0 | 6.0 | 15.1 | 0.20 |
| 69 | 263 | 5 | 4 | 262 | 3 | 3 | 0 | 35.4 | 15.1 | 1.17 |
| 70 | 209 | 19 | 14 | 219 | 17 | 3 | 0 | -18.5 | 15.1 | 0.61 |
| 71 | 208 | 16 | 12 | 209 | 7 | 3 | 0 | -19.5 | 15.1 | 0.65 |
| 72 | 215 | 0 | 0 | 218 | 3 | 3 | 0 | -12.5 | 15.1 | 0.41 |
| 73 | 219 | 1 | 1 | 219 | 1 | 3 | 0 | -8.5 | 15.1 | 0.28 |
| 74 | 253 | 2 | 1 | 252 | 1 | 3 | 0 | 25.0 | 15.1 | 0.83 |
| 75 | 237 | 12 | 8 | 240 | 7 | 3 | 0 | 9.6 | 15.1 | 0.32 |
| 76 | 251 | 4 | 3 | 251 | 2 | 3 | 0 | 23.7 | 15.1 | 0.78 |
| 77 | 217 | 21 | 15 | 223 | 13 | 3 | 0 | -10.5 | 15.1 | 0.35 |
| 78 | 226 | 4 | 3 | 228 | 4 | 3 | 0 | -1.5 | 15.1 | 0.05 |
| 79 | 225 | 4 | 3 | 227 | 4 | 3 | 0 | -2.5 | 15.1 | 0.08 |
| 80 | 135 | 6 | 4 | 134 | 3 | 3 | 0 | -92.5 | 15.1 | 3.06 |
| 81 | 230 | 5 | 4 | 230 | 2 | 3 | 0 | 2.5 | 15.1 | 0.08 |
| 83 | 265 | 9 | 6 | 262 | 5 | 3 | 0 | 37.2 | 15.1 | 1.23 |
| 84 | 319 | 18 | 13 | 305 | 20 | 3 | 0 | 91.2 | 15.1 | 3.02 |
| 85 | 216 | 6 | 4 | 224 | 10 | 3 | 0 | -11.5 | 15.1 | 0.38 |
| 86 | 243 | 4 | 3 | 244 | 3 | 3 | 0 | 15.5 | 15.1 | 0.51 |
| 87 | 237 | 1 | 1 | 237 | 1 | 3 | 0 | 9.5 | 15.1 | 0.31 |
| 88 | 216 | 5 | 4 | 231 | 17 | 3 | 0 | -11.8 | 15.1 | 0.39 |
| 89 | 238 | 24 | 17 | 235 | 12 | 3 | 0 | 10.5 | 15.1 | 0.35 |
| 90 | 236 | 1 | 1 | 235 | 1 | 3 | 0 | 8 | 15.09697 | 0.26 |

**Serine**

| **Lab** | **Median** | **MAD** | **u (median)** | **Mean** | **u (mean)** | **n** | **Non-detects** | **DoE** | **u (DoE)** | **Score** |
| --- | --- | --- | --- | --- | --- | --- | --- | --- | --- | --- |
| 1 | 114.3 | 0.6 | 0.4 | 113.5 | 1.0 | 3 | 0 | 7.4 | 6.9 | 0.53 |
| 2 | 102.0 | 1.5 | 1.1 | 102.7 | 1.2 | 3 | 0 | -4.9 | 6.9 | 0.36 |
| 3 | 102.0 | 3.0 | 2.1 | 104.7 | 3.7 | 3 | 0 | -4.9 | 6.9 | 0.36 |
| 4 | 90.9 | 3.1 | 2.3 | 91.9 | 2.2 | 3 | 0 | -16.0 | 6.9 | 1.16 |
| 5 | 108.0 | 8.9 | 6.4 | 107.0 | 4.4 | 3 | 0 | 1.1 | 6.9 | 0.08 |
| 6 | 107.0 | 3.0 | 2.1 | 107.0 | 1.2 | 3 | 0 | 0.1 | 6.9 | 0.00 |
| 7 | 121.0 | 0.0 | 0.0 | 125.7 | 4.7 | 3 | 0 | 14.1 | 6.9 | 1.01 |
| 8 | 104.0 | 1.5 | 1.1 | 104.0 | 0.6 | 3 | 0 | -2.9 | 6.9 | 0.21 |
| 9 | 136.1 | 31.8 | 23.0 | 155.6 | 30.9 | 3 | 0 | 29.1 | 6.9 | 2.10 |
| 10 | 101.9 | 1.3 | 0.9 | 102.2 | 0.8 | 3 | 0 | -5.1 | 6.9 | 0.36 |
| 11 | 108.0 | 10.4 | 7.5 | 112.0 | 7.8 | 3 | 0 | 1.1 | 6.9 | 0.08 |
| 12 | 109.9 | 3.1 | 2.3 | 109.3 | 1.8 | 3 | 0 | 2.9 | 6.9 | 0.21 |
| 13 | 112.0 | 1.5 | 1.1 | 113.0 | 1.5 | 3 | 0 | 5.1 | 6.9 | 0.36 |
| 14 | 103.0 | 1.5 | 1.1 | 106.0 | 3.5 | 3 | 0 | -3.9 | 6.9 | 0.28 |
| 15 | 105.8 | 0.3 | 0.2 | 106.8 | 1.1 | 3 | 0 | -1.1 | 6.9 | 0.08 |
| 16 | 100.0 | 3.0 | 2.1 | 101.0 | 2.1 | 3 | 0 | -6.9 | 6.9 | 0.50 |
| 17 | 116.0 | 4.4 | 3.2 | 115.3 | 2.3 | 3 | 0 | 9.1 | 6.9 | 0.65 |
| 18 | 108.0 | 3.0 | 2.1 | 107.7 | 1.5 | 3 | 0 | 1.1 | 6.9 | 0.08 |
| 19 | 108.1 | 0.5 | 0.4 | 109.8 | 1.9 | 3 | 0 | 1.2 | 6.9 | 0.08 |
| 20 | 97.6 | 4.0 | 2.9 | 117.7 | 21.4 | 3 | 0 | -9.3 | 6.9 | 0.67 |
| 21 | 108.0 | 5.9 | 4.3 | 107.7 | 2.6 | 3 | 0 | 1.1 | 6.9 | 0.08 |
| 22 | 116.0 | 0.0 | 0.0 | 115.3 | 0.7 | 3 | 0 | 9.1 | 6.9 | 0.65 |
| 23 | 98.9 | 1.3 | 1.0 | 100.1 | 1.7 | 3 | 0 | -8.0 | 6.9 | 0.58 |
| 24 | 103.0 | 0.0 | 0.0 | 105.0 | 2.0 | 3 | 0 | -3.9 | 6.9 | 0.28 |
| 25 | 112.0 | 0.0 | 0.0 | 111.3 | 0.7 | 3 | 0 | 5.1 | 6.9 | 0.36 |
| 26 | 101.1 | 1.6 | 1.2 | 101.7 | 1.3 | 3 | 0 | -5.9 | 6.9 | 0.42 |
| 27 | 112.8 | 2.0 | 1.4 | 113.1 | 1.0 | 3 | 0 | 5.8 | 6.9 | 0.42 |
| 28 | 72.0 | 16.3 | 11.8 | 70.3 | 7.8 | 3 | 0 | -34.9 | 6.9 | 2.52 |
| 29 | 98.0 | 6.5 | 4.7 | 100.5 | 4.9 | 3 | 0 | -8.9 | 6.9 | 0.64 |
| 30 | 106.5 | 10.2 | 7.4 | 106.2 | 4.3 | 3 | 0 | -0.4 | 6.9 | 0.03 |
| 31 | 113.0 | 0.0 | 0.0 | 114.3 | 1.3 | 3 | 0 | 6.1 | 6.9 | 0.44 |
| 32 | 116.2 | 1.0 | 0.8 | 116.2 | 0.4 | 3 | 0 | 9.3 | 6.9 | 0.67 |
| 33 | 105.0 | 3.0 | 2.1 | 103.7 | 2.4 | 3 | 0 | -1.9 | 6.9 | 0.14 |
| 34 | 117.0 | 11.9 | 8.6 | 108.3 | 12.9 | 3 | 0 | 10.1 | 6.9 | 0.72 |
| 35 | 105.0 | 3.0 | 2.1 | 104.3 | 1.8 | 3 | 0 | -1.9 | 6.9 | 0.14 |
| 36 | 108.8 | 0.9 | 0.7 | 109.1 | 0.7 | 3 | 0 | 1.8 | 6.9 | 0.13 |
| 37 | 104.5 | 2.0 | 1.4 | 105.0 | 1.2 | 3 | 0 | -2.5 | 6.9 | 0.18 |
| 38 | 109.0 | 3.0 | 2.1 | 110.3 | 2.4 | 3 | 0 | 2.1 | 6.9 | 0.15 |
| 39 | 88.4 | 0.9 | 0.6 | 88.6 | 0.5 | 3 | 0 | -18.5 | 6.9 | 1.34 |
| 40 | 113.0 | 2.2 | 1.6 | 112.5 | 1.3 | 3 | 0 | 6.1 | 6.9 | 0.44 |
| 41 | 107.8 | 3.6 | 2.6 | 104.0 | 5.0 | 3 | 0 | 0.9 | 6.9 | 0.06 |
| 42 | 111.4 | 6.7 | 4.8 | 111.3 | 2.7 | 3 | 0 | 4.5 | 6.9 | 0.32 |
| 43 | 99.3 | 2.3 | 1.7 | 99.1 | 1.1 | 3 | 0 | -7.7 | 6.9 | 0.55 |
| 44 | 105.0 | 5.9 | 4.3 | 105.7 | 2.9 | 3 | 0 | -1.9 | 6.9 | 0.14 |
| **Serine** | | | | | | | | | | |
| 45 | 107.5 | 2.4 | 1.7 | 107.1 | 1.3 | 3 | 0 | 0.6 | 6.9 | 0.04 |
| 46 | 109.6 | 3.3 | 2.4 | 109.4 | 1.5 | 3 | 0 | 2.7 | 6.9 | 0.19 |
| 47 | 120.0 | 1.5 | 1.1 | 118.3 | 2.2 | 3 | 0 | 13.1 | 6.9 | 0.94 |
| 48 | 112.0 | 4.2 | 3.0 | 113.3 | 2.9 | 3 | 0 | 5.1 | 6.9 | 0.36 |
| 49 | 124.0 | 5.9 | 4.3 | 130.7 | 8.7 | 3 | 0 | 17.1 | 6.9 | 1.23 |
| 50 | 110.0 | 7.4 | 5.4 | 110.7 | 3.5 | 3 | 0 | 3.1 | 6.9 | 0.22 |
| 51 | 108.0 | 1.5 | 1.1 | 107.0 | 1.5 | 3 | 0 | 1.1 | 6.9 | 0.08 |
| 52 | 116.0 | 3.0 | 2.1 | 117.0 | 2.1 | 3 | 0 | 9.1 | 6.9 | 0.65 |
| 53 | 103.8 | 1.6 | 1.2 | 102.5 | 1.8 | 3 | 0 | -3.1 | 6.9 | 0.23 |
| 54 | 106.9 | 1.0 | 0.7 | 107.1 | 0.6 | 3 | 0 | 0.0 | 6.9 | 0.00 |
| 55 | 104.0 | 0.0 | 0.0 | 104.0 | 0.0 | 3 | 0 | -2.9 | 6.9 | 0.21 |
| 56 | 101.2 | 1.9 | 1.4 | 100.8 | 1.1 | 3 | 0 | -5.7 | 6.9 | 0.41 |
| 57 | 107.0 | 3.6 | 2.6 | 107.1 | 1.5 | 3 | 0 | 0.1 | 6.9 | 0.00 |
| 58 | 106.2 | 0.3 | 0.2 | 104.1 | 2.2 | 3 | 0 | -0.7 | 6.9 | 0.05 |
| 59 | 103.0 | 3.0 | 2.1 | 102.3 | 1.8 | 3 | 0 | -3.9 | 6.9 | 0.28 |
| 60 | 106.0 | 13.3 | 9.7 | 105.0 | 6.1 | 3 | 0 | -0.9 | 6.9 | 0.07 |
| 61 | 94.2 | 3.9 | 2.8 | 93.4 | 2.2 | 3 | 0 | -12.7 | 6.9 | 0.92 |
| 62 | 99.0 | 4.4 | 3.2 | 101.0 | 3.6 | 3 | 0 | -7.9 | 6.9 | 0.57 |
| 63 | 102.6 | 0.4 | 0.3 | 103.3 | 0.8 | 3 | 0 | -4.3 | 6.9 | 0.31 |
| 64 | 106.0 | 3.0 | 2.1 | 99.0 | 8.0 | 3 | 0 | -0.9 | 6.9 | 0.07 |
| 65 | 112.4 | 2.8 | 2.0 | 113.5 | 2.1 | 3 | 0 | 5.5 | 6.9 | 0.39 |
| 66 | 122.0 | 5.9 | 4.3 | 121.7 | 2.6 | 3 | 0 | 15.1 | 6.9 | 1.09 |
| 67 | 100.0 | 1.5 | 1.1 | 95.9 | 4.6 | 3 | 0 | -6.9 | 6.9 | 0.50 |
| 68 | 116.2 | 2.8 | 2.1 | 113.6 | 3.7 | 3 | 0 | 9.3 | 6.9 | 0.67 |
| 69 | 124.2 | 13.7 | 9.9 | 127.1 | 8.0 | 3 | 0 | 17.2 | 6.9 | 1.24 |
| 70 | 106.0 | 0.0 | 0.0 | 103.0 | 3.0 | 3 | 0 | -0.9 | 6.9 | 0.07 |
| 71 | 86.0 | 3.0 | 2.1 | 86.0 | 1.2 | 3 | 0 | -20.9 | 6.9 | 1.51 |
| 72 | 99.0 | 1.5 | 1.1 | 99.7 | 1.2 | 3 | 0 | -7.9 | 6.9 | 0.57 |
| 73 | 100.0 | 1.5 | 1.1 | 99.7 | 0.9 | 3 | 0 | -6.9 | 6.9 | 0.50 |
| 74 | 91.0 | 3.9 | 2.8 | 92.1 | 2.5 | 3 | 0 | -15.9 | 6.9 | 1.15 |
| 75 | 113.5 | 0.4 | 0.3 | 115.7 | 2.3 | 3 | 0 | 6.6 | 6.9 | 0.47 |
| 76 | 114.4 | 0.9 | 0.6 | 116.5 | 2.4 | 3 | 0 | 7.5 | 6.9 | 0.54 |
| 77 | 108.0 | 4.4 | 3.2 | 109.0 | 2.6 | 3 | 0 | 1.1 | 6.9 | 0.08 |
| 78 | 104.0 | 3.0 | 2.1 | 103.7 | 1.5 | 3 | 0 | -2.9 | 6.9 | 0.21 |
| 79 | 101.0 | 0.0 | 0.0 | 101.3 | 0.3 | 3 | 0 | -5.9 | 6.9 | 0.43 |
| 80 | 61.0 | 0.0 | 0.0 | 63.3 | 2.3 | 3 | 0 | -45.9 | 6.9 | 3.31 |
| 81 | 109.8 | 1.0 | 0.8 | 109.8 | 0.4 | 3 | 0 | 2.9 | 6.9 | 0.21 |
| 83 | 117.1 | 0.1 | 0.1 | 119.2 | 2.2 | 3 | 0 | 10.2 | 6.9 | 0.73 |
| 84 | 103.9 | 5.1 | 3.7 | 104.0 | 2.1 | 3 | 0 | -3.0 | 6.9 | 0.22 |
| 85 | 104.0 | 14.8 | 10.7 | 107.0 | 8.5 | 3 | 0 | -2.9 | 6.9 | 0.21 |
| 86 | 113.7 | 1.9 | 1.4 | 112.9 | 1.5 | 3 | 0 | 6.8 | 6.9 | 0.49 |
| 87 | 105.0 | 3.0 | 2.1 | 104.3 | 1.8 | 3 | 0 | -1.9 | 6.9 | 0.14 |
| 88 | 96.1 | 4.7 | 3.4 | 98.4 | 4.0 | 3 | 0 | -10.9 | 6.9 | 0.79 |
| 89 | 119.0 | 8.9 | 6.4 | 115.7 | 6.6 | 3 | 0 | 12.1 | 6.9 | 0.87 |
| 90 | 120.4 | 12.2 | 8.8 | 126.2 | 10.2 | 3 | 0 | 13.5 | 6.9 | 0.97 |

**Taurine**

| **Lab** | **Median** | **MAD** | **u (median)** | **Mean** | **u (mean)** | **n** | **Non-detects** | **DoE** | **u (DoE)** | **Score** |
| --- | --- | --- | --- | --- | --- | --- | --- | --- | --- | --- |
| 1 | 47.8 | 0.9 | 0.6 | 47.8 | 0.4 | 3 | 0 | -0.2 | 2.7 | 0.04 |
| 2 | 48.9 | 0.3 | 0.2 | 49.4 | 0.6 | 3 | 0 | 0.9 | 2.7 | 0.16 |
| 3 | 47.0 | 1.5 | 1.1 | 48.3 | 1.9 | 3 | 0 | -1.0 | 2.7 | 0.18 |
| 4 | 53.5 | 7.9 | 5.7 | 50.8 | 5.6 | 3 | 0 | 5.5 | 2.7 | 1.00 |
| 5 | 70.0 | 3.0 | 2.1 | 64.0 | 7.0 | 3 | 0 | 22.0 | 2.7 | 4.01 |
| 6 | 47.0 | 3.0 | 2.1 | 46.7 | 1.5 | 3 | 0 | -1.0 | 2.7 | 0.18 |
| 7 | 48.0 | 1.5 | 1.1 | 48.0 | 0.6 | 3 | 0 | 0.0 | 2.7 | 0.00 |
| 8 | 46.0 | 1.5 | 1.1 | 46.3 | 0.9 | 3 | 0 | -2.0 | 2.7 | 0.36 |
| 10 | 49.8 | 1.9 | 1.4 | 50.3 | 1.2 | 3 | 0 | 1.8 | 2.7 | 0.33 |
| 11 | 49.6 | 0.4 | 0.3 | 49.6 | 0.2 | 3 | 0 | 1.6 | 2.7 | 0.29 |
| 12 | 44.3 | 1.9 | 1.4 | 47.1 | 3.5 | 3 | 0 | -3.7 | 2.7 | 0.67 |
| 13 | 51.0 | 3.0 | 2.1 | 50.0 | 2.1 | 3 | 0 | 3.0 | 2.7 | 0.55 |
| 14 | 46.0 | 1.5 | 1.1 | 46.7 | 1.2 | 3 | 0 | -2.0 | 2.7 | 0.36 |
| 15 | 47.4 | 0.2 | 0.2 | 47.1 | 0.4 | 3 | 0 | -0.6 | 2.7 | 0.11 |
| 16 | 54.0 | 3.0 | 2.1 | 53.0 | 2.1 | 3 | 0 | 6.0 | 2.7 | 1.09 |
| 17 | 47.0 | 1.5 | 1.1 | 49.0 | 2.5 | 3 | 0 | -1.0 | 2.7 | 0.18 |
| 19 | 51.6 | 3.9 | 2.8 | 51.3 | 1.8 | 3 | 0 | 3.6 | 2.7 | 0.66 |
| 20 | 58.2 | 23.9 | 17.3 | 44.2 | 22.6 | 3 | 1 | 10.2 | 2.7 | 1.86 |
| 21 | 48.0 | 1.5 | 1.1 | 48.3 | 0.9 | 3 | 0 | 0.0 | 2.7 | 0.00 |
| 22 | 53.0 | 0.0 | 0.0 | 52.3 | 0.7 | 3 | 0 | 5.0 | 2.7 | 0.91 |
| 23 | 46.9 | 1.2 | 0.9 | 47.5 | 1.0 | 3 | 0 | -1.1 | 2.7 | 0.20 |
| 24 | 48.0 | 0.0 | 0.0 | 49.0 | 1.0 | 3 | 0 | 0.0 | 2.7 | 0.00 |
| 25 | 50.0 | 0.0 | 0.0 | 50.3 | 0.3 | 3 | 0 | 2.0 | 2.7 | 0.36 |
| 26 | 45.3 | 0.8 | 0.6 | 45.5 | 0.5 | 3 | 0 | -2.7 | 2.7 | 0.50 |
| 27 | 57.0 | 0.8 | 0.6 | 56.7 | 0.6 | 3 | 0 | 9.0 | 2.7 | 1.64 |
| 28 | 48.0 | 1.5 | 1.1 | 47.0 | 1.5 | 3 | 0 | 0.0 | 2.7 | 0.00 |
| 29 | 44.5 | 1.2 | 0.9 | 44.6 | 0.6 | 3 | 0 | -3.5 | 2.7 | 0.64 |
| 30 | 38.0 | 1.2 | 0.9 | 39.4 | 1.8 | 3 | 0 | -10.0 | 2.7 | 1.82 |
| 31 | 59.0 | 14.8 | 10.7 | 68.0 | 14.3 | 3 | 0 | 11.0 | 2.7 | 2.00 |
| 32 | 55.9 | 0.3 | 0.2 | 55.5 | 0.5 | 3 | 0 | 7.9 | 2.7 | 1.44 |
| 33 | 46.0 | 0.0 | 0.0 | 45.3 | 0.7 | 3 | 0 | -2.0 | 2.7 | 0.36 |
| 35 | 45.0 | 0.0 | 0.0 | 45.3 | 0.3 | 3 | 0 | -3.0 | 2.7 | 0.55 |
| 36 | 48.8 | 0.3 | 0.2 | 49.1 | 0.5 | 3 | 0 | 0.8 | 2.7 | 0.14 |
| 37 | 51.2 | 0.2 | 0.2 | 49.0 | 2.3 | 3 | 0 | 3.2 | 2.7 | 0.59 |
| 38 | 48.0 | 1.5 | 1.1 | 49.0 | 1.5 | 3 | 0 | 0.0 | 2.7 | 0.00 |
| 40 | 51.0 | 1.5 | 1.1 | 50.8 | 0.7 | 3 | 0 | 3.0 | 2.7 | 0.55 |
| 41 | 56.6 | 11.9 | 8.6 | 56.7 | 4.7 | 3 | 0 | 8.6 | 2.7 | 1.57 |
| 42 | 49.6 | 2.1 | 1.5 | 49.4 | 1.0 | 3 | 0 | 1.6 | 2.7 | 0.29 |
| 43 | 45.3 | 1.3 | 0.9 | 45.3 | 0.5 | 3 | 0 | -2.7 | 2.7 | 0.49 |
| 44 | 49.0 | 0.0 | 0.0 | 48.3 | 0.7 | 3 | 0 | 1.0 | 2.7 | 0.18 |
| 45 | 48.7 | 0.1 | 0.1 | 48.6 | 0.2 | 3 | 0 | 0.7 | 2.7 | 0.13 |
| 46 | 46.3 | 1.0 | 0.8 | 45.3 | 1.3 | 3 | 0 | -1.7 | 2.7 | 0.31 |
| 47 | 59.0 | 0.0 | 0.0 | 58.3 | 0.7 | 3 | 0 | 11.0 | 2.7 | 2.00 |
| 48 | 47.1 | 2.4 | 1.7 | 47.4 | 1.2 | 3 | 0 | -0.9 | 2.7 | 0.16 |
| **Taurine** | | | | | | | | | | |
| 49 | 46.5 | 11.9 | 8.6 | 47.5 | 5.5 | 3 | 0 | -1.5 | 2.7 | 0.27 |
| 50 | 69.0 | 3.0 | 2.1 | 68.3 | 1.8 | 3 | 0 | 21.0 | 2.7 | 3.83 |
| 51 | 40.0 | 3.0 | 2.1 | 40.3 | 1.5 | 3 | 0 | -8.0 | 2.7 | 1.46 |
| 52 | 52.0 | 0.0 | 0.0 | 53.3 | 1.3 | 3 | 0 | 4.0 | 2.7 | 0.73 |
| 53 | 44.7 | 3.6 | 2.6 | 46.8 | 3.4 | 3 | 0 | -3.3 | 2.7 | 0.60 |
| 54 | 51.5 | 0.9 | 0.6 | 51.1 | 0.8 | 3 | 0 | 3.5 | 2.7 | 0.65 |
| 55 | 48.0 | 0.0 | 0.0 | 47.7 | 0.3 | 3 | 0 | 0.0 | 2.7 | 0.00 |
| 56 | 46.7 | 1.3 | 1.0 | 46.5 | 0.8 | 3 | 0 | -1.3 | 2.7 | 0.23 |
| 57 | 51.2 | 2.2 | 1.6 | 51.2 | 0.9 | 3 | 0 | 3.2 | 2.7 | 0.58 |
| 58 | 49.7 | 1.4 | 1.0 | 49.3 | 0.9 | 3 | 0 | 1.7 | 2.7 | 0.30 |
| 59 | 48.0 | 0.0 | 0.0 | 47.3 | 0.7 | 3 | 0 | 0.0 | 2.7 | 0.00 |
| 60 | 48.0 | 1.5 | 1.1 | 48.3 | 0.9 | 3 | 0 | 0.0 | 2.7 | 0.00 |
| 61 | 0.0 | 0.0 | 0.0 | 0.0 | 0.0 | 3 | 1 | -48.0 | 2.7 | 8.74 |
| 62 | 42.0 | 1.5 | 1.1 | 42.7 | 1.2 | 3 | 0 | -6.0 | 2.7 | 1.09 |
| 63 | 46.8 | 0.1 | 0.1 | 47.1 | 0.4 | 3 | 0 | -1.2 | 2.7 | 0.22 |
| 64 | 47.0 | 1.5 | 1.1 | 52.7 | 6.2 | 3 | 0 | -1.0 | 2.7 | 0.18 |
| 65 | 46.8 | 2.0 | 1.4 | 46.2 | 1.3 | 3 | 0 | -1.2 | 2.7 | 0.23 |
| 66 | 60.2 | 1.9 | 1.4 | 59.9 | 1.0 | 3 | 0 | 12.2 | 2.7 | 2.22 |
| 67 | 50.2 | 4.4 | 3.2 | 49.9 | 2.0 | 3 | 0 | 2.2 | 2.7 | 0.40 |
| 68 | 50.2 | 3.6 | 2.6 | 50.8 | 1.9 | 3 | 0 | 2.2 | 2.7 | 0.41 |
| 69 | 52.4 | 0.3 | 0.2 | 52.3 | 0.2 | 3 | 0 | 4.4 | 2.7 | 0.80 |
| 70 | 45.0 | 3.0 | 2.1 | 45.3 | 1.5 | 3 | 0 | -3.0 | 2.7 | 0.55 |
| 72 | 62.0 | 5.9 | 4.3 | 59.3 | 4.8 | 3 | 0 | 14.0 | 2.7 | 2.55 |
| 73 | 47.0 | 1.5 | 1.1 | 46.7 | 0.9 | 3 | 0 | -1.0 | 2.7 | 0.18 |
| 74 | 51.0 | 0.7 | 0.5 | 50.6 | 0.7 | 3 | 0 | 3.0 | 2.7 | 0.55 |
| 75 | 49.7 | 0.3 | 0.2 | 50.3 | 0.7 | 3 | 0 | 1.7 | 2.7 | 0.31 |
| 76 | 50.2 | 0.3 | 0.2 | 49.9 | 0.4 | 3 | 0 | 2.2 | 2.7 | 0.40 |
| 77 | 49.0 | 1.5 | 1.1 | 49.7 | 1.2 | 3 | 0 | 1.0 | 2.7 | 0.18 |
| 78 | 48.0 | 3.0 | 2.1 | 48.0 | 1.2 | 3 | 0 | 0.0 | 2.7 | 0.00 |
| 79 | 46.0 | 0.0 | 0.0 | 46.0 | 0.0 | 3 | 0 | -2.0 | 2.7 | 0.36 |
| 80 | 27.0 | 0.0 | 0.0 | 26.7 | 0.3 | 3 | 0 | -21.0 | 2.7 | 3.83 |
| 81 | 44.5 | 3.7 | 2.7 | 43.9 | 2.0 | 3 | 0 | -3.5 | 2.7 | 0.64 |
| 83 | 47.5 | 0.6 | 0.4 | 47.4 | 0.3 | 3 | 0 | -0.5 | 2.7 | 0.09 |
| 84 | 61.1 | 2.7 | 2.0 | 61.7 | 1.6 | 3 | 0 | 13.1 | 2.7 | 2.38 |
| 85 | 47.0 | 1.5 | 1.1 | 47.3 | 0.9 | 3 | 0 | -1.0 | 2.7 | 0.18 |
| 86 | 49.0 | 3.0 | 2.1 | 49.3 | 1.4 | 3 | 0 | 1.0 | 2.7 | 0.18 |
| 87 | 47.0 | 0.0 | 0.0 | 47.3 | 0.3 | 3 | 0 | -1.0 | 2.7 | 0.18 |
| 88 | 43.9 | 1.5 | 1.1 | 43.4 | 1.1 | 3 | 0 | -4.1 | 2.7 | 0.75 |
| 89 | 50.0 | 1.5 | 1.1 | 49.0 | 1.5 | 3 | 0 | 2.0 | 2.7 | 0.36 |
| 90 | 58.2 | 0.4 | 0.3 | 64.5 | 6.5 | 3 | 0 | 10.2 | 2.7 | 1.86 |

**Threonine**

| **Lab** | **Median** | **MAD** | **u (median)** | **Mean** | **u (mean)** | **n** | **Non-detects** | **DoE** | **u (DoE)** | **Score** |
| --- | --- | --- | --- | --- | --- | --- | --- | --- | --- | --- |
| 1 | 154.4 | 2.2 | 1.6 | 154.0 | 1.3 | 3 | 0 | 6.9 | 7.6 | 0.46 |
| 2 | 145.0 | 1.5 | 1.1 | 145.7 | 1.2 | 3 | 0 | -2.5 | 7.6 | 0.16 |
| 3 | 140.0 | 0.0 | 0.0 | 145.0 | 5.0 | 3 | 0 | -7.5 | 7.6 | 0.49 |
| 4 | 144.0 | 4.4 | 3.2 | 141.7 | 3.9 | 3 | 0 | -3.5 | 7.6 | 0.23 |
| 5 | 156.0 | 7.4 | 5.4 | 152.7 | 6.0 | 3 | 0 | 8.5 | 7.6 | 0.56 |
| 6 | 147.0 | 0.0 | 0.0 | 147.7 | 0.7 | 3 | 0 | -0.5 | 7.6 | 0.03 |
| 7 | 149.0 | 3.0 | 2.1 | 147.0 | 3.1 | 3 | 0 | 1.5 | 7.6 | 0.10 |
| 8 | 145.0 | 3.0 | 2.1 | 144.3 | 1.8 | 3 | 0 | -2.5 | 7.6 | 0.16 |
| 9 | 180.4 | 20.9 | 15.2 | 213.7 | 40.6 | 3 | 0 | 32.9 | 7.6 | 2.16 |
| 10 | 149.2 | 2.1 | 1.5 | 149.2 | 0.8 | 3 | 0 | 1.7 | 7.6 | 0.11 |
| 11 | 151.0 | 5.9 | 4.3 | 156.0 | 7.1 | 3 | 0 | 3.5 | 7.6 | 0.23 |
| 12 | 142.0 | 3.0 | 2.1 | 142.9 | 2.0 | 3 | 0 | -5.5 | 7.6 | 0.36 |
| 13 | 157.0 | 0.0 | 0.0 | 155.3 | 1.7 | 3 | 0 | 9.5 | 7.6 | 0.63 |
| 14 | 143.0 | 3.0 | 2.1 | 142.7 | 1.5 | 3 | 0 | -4.5 | 7.6 | 0.29 |
| 15 | 147.8 | 1.0 | 0.7 | 147.1 | 1.1 | 3 | 0 | 0.3 | 7.6 | 0.02 |
| 16 | 145.0 | 0.0 | 0.0 | 145.3 | 0.3 | 3 | 0 | -2.5 | 7.6 | 0.16 |
| 17 | 156.0 | 1.5 | 1.1 | 156.3 | 0.9 | 3 | 0 | 8.5 | 7.6 | 0.56 |
| 18 | 153.0 | 19.3 | 13.9 | 145.3 | 14.7 | 3 | 0 | 5.5 | 7.6 | 0.36 |
| 19 | 152.1 | 4.2 | 3.0 | 153.4 | 2.9 | 3 | 0 | 4.6 | 7.6 | 0.30 |
| 20 | 140.1 | 5.0 | 3.6 | 144.7 | 6.3 | 3 | 0 | -7.4 | 7.6 | 0.48 |
| 21 | 162.0 | 3.0 | 2.1 | 159.3 | 3.7 | 3 | 0 | 14.5 | 7.6 | 0.96 |
| 22 | 160.0 | 0.0 | 0.0 | 160.3 | 0.3 | 3 | 0 | 12.5 | 7.6 | 0.82 |
| 23 | 140.7 | 3.7 | 2.7 | 141.1 | 1.8 | 3 | 0 | -6.8 | 7.6 | 0.44 |
| 24 | 145.0 | 0.0 | 0.0 | 147.7 | 2.7 | 3 | 0 | -2.5 | 7.6 | 0.16 |
| 25 | 155.0 | 3.0 | 2.1 | 155.0 | 1.2 | 3 | 0 | 7.5 | 7.6 | 0.50 |
| 26 | 141.5 | 0.7 | 0.5 | 141.7 | 0.5 | 3 | 0 | -6.0 | 7.6 | 0.39 |
| 27 | 157.7 | 0.4 | 0.3 | 157.2 | 0.7 | 3 | 0 | 10.3 | 7.6 | 0.68 |
| 28 | 115.0 | 3.0 | 2.1 | 117.7 | 3.7 | 3 | 0 | -32.5 | 7.6 | 2.13 |
| 29 | 134.2 | 3.1 | 2.3 | 136.7 | 3.6 | 3 | 0 | -13.3 | 7.6 | 0.87 |
| 30 | 152.1 | 12.5 | 9.0 | 151.9 | 5.0 | 3 | 0 | 4.6 | 7.6 | 0.31 |
| 31 | 163.0 | 4.4 | 3.2 | 167.0 | 5.6 | 3 | 0 | 15.5 | 7.6 | 1.02 |
| 32 | 153.4 | 0.1 | 0.1 | 152.9 | 0.5 | 3 | 0 | 5.9 | 7.6 | 0.39 |
| 33 | 146.0 | 1.5 | 1.1 | 144.0 | 2.5 | 3 | 0 | -1.5 | 7.6 | 0.10 |
| 34 | 128.0 | 10.4 | 7.5 | 123.0 | 8.7 | 3 | 0 | -19.5 | 7.6 | 1.28 |
| 35 | 142.0 | 3.0 | 2.1 | 141.3 | 1.8 | 3 | 0 | -5.5 | 7.6 | 0.36 |
| 36 | 151.9 | 1.6 | 1.2 | 152.2 | 1.0 | 3 | 0 | 4.4 | 7.6 | 0.29 |
| 37 | 147.5 | 0.1 | 0.1 | 145.7 | 1.8 | 3 | 0 | 0.0 | 7.6 | 0.00 |
| 38 | 155.0 | 0.0 | 0.0 | 153.3 | 1.7 | 3 | 0 | 7.5 | 7.6 | 0.50 |
| 39 | 134.0 | 3.0 | 2.1 | 134.7 | 1.8 | 3 | 0 | -13.5 | 7.6 | 0.89 |
| 40 | 156.5 | 4.4 | 3.2 | 157.0 | 2.2 | 3 | 0 | 9.0 | 7.6 | 0.59 |
| 41 | 132.9 | 0.3 | 0.2 | 137.4 | 4.6 | 3 | 0 | -14.6 | 7.6 | 0.96 |
| 42 | 153.5 | 3.6 | 2.6 | 151.7 | 3.1 | 3 | 0 | 6.0 | 7.6 | 0.40 |
| 43 | 135.0 | 1.7 | 1.2 | 134.0 | 1.7 | 3 | 0 | -12.4 | 7.6 | 0.82 |
| 44 | 149.0 | 1.5 | 1.1 | 146.3 | 3.2 | 3 | 0 | 1.5 | 7.6 | 0.10 |
| **Threonine** | | | | | | | | | | |
| 45 | 146.8 | 0.1 | 0.1 | 146.6 | 0.3 | 3 | 0 | -0.7 | 7.6 | 0.04 |
| 46 | 146.6 | 2.4 | 1.7 | 147.3 | 1.5 | 3 | 0 | -0.9 | 7.6 | 0.06 |
| 47 | 152.0 | 1.5 | 1.1 | 147.3 | 5.2 | 3 | 0 | 4.5 | 7.6 | 0.30 |
| 48 | 139.7 | 3.0 | 2.1 | 140.2 | 1.6 | 3 | 0 | -7.8 | 7.6 | 0.51 |
| 49 | 138.0 | 5.9 | 4.3 | 139.7 | 3.8 | 3 | 0 | -9.5 | 7.6 | 0.62 |
| 50 | 155.0 | 3.0 | 2.1 | 153.0 | 3.1 | 3 | 0 | 7.5 | 7.6 | 0.50 |
| 51 | 152.0 | 3.0 | 2.1 | 152.0 | 1.2 | 3 | 0 | 4.5 | 7.6 | 0.30 |
| 52 | 149.0 | 3.0 | 2.1 | 151.7 | 3.7 | 3 | 0 | 1.5 | 7.6 | 0.10 |
| 53 | 129.1 | 4.9 | 3.5 | 131.2 | 3.8 | 3 | 0 | -18.4 | 7.6 | 1.21 |
| 54 | 155.7 | 0.7 | 0.5 | 153.3 | 2.6 | 3 | 0 | 8.2 | 7.6 | 0.54 |
| 55 | 147.0 | 3.0 | 2.1 | 147.0 | 1.2 | 3 | 0 | -0.5 | 7.6 | 0.03 |
| 56 | 142.4 | 4.2 | 3.0 | 141.9 | 2.0 | 3 | 0 | -5.1 | 7.6 | 0.34 |
| 57 | 138.4 | 3.4 | 2.5 | 139.4 | 2.2 | 3 | 0 | -9.1 | 7.6 | 0.60 |
| 58 | 148.2 | 1.4 | 1.0 | 147.7 | 1.0 | 3 | 0 | 0.7 | 7.6 | 0.05 |
| 59 | 144.0 | 1.5 | 1.1 | 142.3 | 2.2 | 3 | 0 | -3.5 | 7.6 | 0.23 |
| 60 | 156.0 | 4.4 | 3.2 | 152.3 | 5.2 | 3 | 0 | 8.5 | 7.6 | 0.56 |
| 61 | 0.0 | 0.0 | 0.0 | 0.0 | 0.0 | 3 | 1 | -147.5 | 7.6 | 9.70 |
| 62 | 134.0 | 4.4 | 3.2 | 135.3 | 3.0 | 3 | 0 | -13.5 | 7.6 | 0.89 |
| 63 | 144.9 | 1.5 | 1.1 | 144.6 | 0.9 | 3 | 0 | -2.6 | 7.6 | 0.17 |
| 64 | 144.0 | 0.0 | 0.0 | 141.0 | 3.0 | 3 | 0 | -3.5 | 7.6 | 0.23 |
| 65 | 152.0 | 0.2 | 0.1 | 149.6 | 2.5 | 3 | 0 | 4.5 | 7.6 | 0.30 |
| 66 | 164.0 | 0.0 | 0.0 | 166.3 | 2.3 | 3 | 0 | 16.5 | 7.6 | 1.09 |
| 67 | 152.0 | 1.5 | 1.1 | 146.7 | 5.8 | 3 | 0 | 4.5 | 7.6 | 0.30 |
| 68 | 157.1 | 3.9 | 2.8 | 160.8 | 5.1 | 3 | 0 | 9.6 | 7.6 | 0.63 |
| 69 | 166.2 | 3.5 | 2.6 | 166.4 | 1.6 | 3 | 0 | 18.7 | 7.6 | 1.23 |
| 70 | 143.0 | 5.9 | 4.3 | 141.3 | 3.8 | 3 | 0 | -4.5 | 7.6 | 0.29 |
| 71 | 135.0 | 1.5 | 1.1 | 138.7 | 4.2 | 3 | 0 | -12.5 | 7.6 | 0.82 |
| 72 | 140.0 | 1.5 | 1.1 | 141.0 | 1.5 | 3 | 0 | -7.5 | 7.6 | 0.49 |
| 73 | 143.0 | 1.5 | 1.1 | 142.3 | 1.2 | 3 | 0 | -4.5 | 7.6 | 0.29 |
| 74 | 175.1 | 3.9 | 2.8 | 173.1 | 3.4 | 3 | 0 | 27.6 | 7.6 | 1.81 |
| 75 | 157.7 | 4.7 | 3.4 | 157.7 | 1.9 | 3 | 0 | 10.2 | 7.6 | 0.67 |
| 76 | 152.0 | 2.2 | 1.6 | 150.8 | 2.0 | 3 | 0 | 4.5 | 7.6 | 0.30 |
| 77 | 141.0 | 5.9 | 4.3 | 142.0 | 3.2 | 3 | 0 | -6.5 | 7.6 | 0.42 |
| 78 | 146.0 | 4.4 | 3.2 | 146.7 | 2.3 | 3 | 0 | -1.5 | 7.6 | 0.10 |
| 79 | 143.0 | 0.0 | 0.0 | 142.7 | 0.3 | 3 | 0 | -4.5 | 7.6 | 0.29 |
| 80 | 91.0 | 0.0 | 0.0 | 89.3 | 1.7 | 3 | 0 | -56.5 | 7.6 | 3.71 |
| 81 | 149.1 | 2.5 | 1.8 | 149.4 | 1.3 | 3 | 0 | 1.6 | 7.6 | 0.11 |
| 83 | 152.1 | 0.6 | 0.4 | 151.8 | 0.5 | 3 | 0 | 4.6 | 7.6 | 0.31 |
| 84 | 144.0 | 5.1 | 3.7 | 145.3 | 3.1 | 3 | 0 | -3.5 | 7.6 | 0.23 |
| 85 | 151.0 | 14.8 | 10.7 | 150.3 | 6.4 | 3 | 0 | 3.5 | 7.6 | 0.23 |
| 86 | 150.0 | 7.4 | 5.4 | 151.7 | 4.4 | 3 | 0 | 2.5 | 7.6 | 0.17 |
| 87 | 145.0 | 4.4 | 3.2 | 144.7 | 2.0 | 3 | 0 | -2.5 | 7.6 | 0.16 |
| 88 | 140.2 | 0.7 | 0.5 | 144.7 | 4.7 | 3 | 0 | -7.3 | 7.6 | 0.48 |
| 89 | 157.0 | 4.4 | 3.2 | 153.0 | 5.6 | 3 | 0 | 9.5 | 7.6 | 0.63 |
| 90 | 152.3 | 5.5 | 4.0 | 152.2 | 2.2 | 3 | 0 | 4.8 | 7.6 | 0.32 |

**Tryptophan**

| **Lab** | **Median** | **MAD** | **u (median)** | **Mean** | **u (mean)** | **n** | **Non-detects** | **DoE** | **u (DoE)** | **Score** |
| --- | --- | --- | --- | --- | --- | --- | --- | --- | --- | --- |
| 1 | 62.4 | 1.8 | 1.3 | 62.4 | 0.7 | 3 | 0 | 10.4 | 9.6 | 0.54 |
| 2 | 38.9 | 0.9 | 0.6 | 39.7 | 1.1 | 3 | 0 | -13.1 | 9.6 | 0.68 |
| 4 | 53.4 | 1.3 | 1.0 | 53.4 | 0.5 | 3 | 0 | 1.4 | 9.6 | 0.07 |
| 5 | 42.0 | 1.5 | 1.1 | 41.0 | 1.5 | 3 | 0 | -10.0 | 9.6 | 0.52 |
| 7 | 52.0 | 0.0 | 0.0 | 53.7 | 1.7 | 3 | 0 | 0.0 | 9.6 | 0.00 |
| 8 | 45.0 | 0.0 | 0.0 | 44.3 | 0.7 | 3 | 0 | -7.0 | 9.6 | 0.36 |
| 9 | 55.1 | 4.7 | 3.4 | 69.9 | 16.5 | 3 | 0 | 3.1 | 9.6 | 0.16 |
| 10 | 34.8 | 0.0 | 0.0 | 35.0 | 0.2 | 3 | 0 | -17.2 | 9.6 | 0.90 |
| 11 | 45.2 | 1.6 | 1.2 | 45.4 | 0.8 | 3 | 0 | -6.8 | 9.6 | 0.35 |
| 12 | 58.0 | 1.5 | 1.1 | 57.3 | 1.3 | 3 | 0 | 6.0 | 9.6 | 0.31 |
| 14 | 49.0 | 1.5 | 1.1 | 49.0 | 0.6 | 3 | 0 | -3.0 | 9.6 | 0.16 |
| 15 | 37.5 | 0.1 | 0.1 | 38.2 | 0.7 | 3 | 0 | -14.5 | 9.6 | 0.76 |
| 16 | 55.0 | 0.0 | 0.0 | 55.3 | 0.3 | 3 | 0 | 3.0 | 9.6 | 0.16 |
| 17 | 51.0 | 4.4 | 3.2 | 51.3 | 2.0 | 3 | 0 | -1.0 | 9.6 | 0.05 |
| 18 | 65.0 | 1.5 | 1.1 | 64.0 | 1.5 | 3 | 0 | 13.0 | 9.6 | 0.68 |
| 19 | 35.8 | 0.7 | 0.5 | 35.9 | 0.3 | 3 | 0 | -16.2 | 9.6 | 0.84 |
| 20 | 60.7 | 0.1 | 0.1 | 63.8 | 3.2 | 3 | 0 | 8.7 | 9.6 | 0.45 |
| 22 | 56.0 | 3.0 | 2.1 | 56.0 | 1.2 | 3 | 0 | 4.0 | 9.6 | 0.21 |
| 23 | 38.0 | 3.6 | 2.6 | 39.1 | 2.4 | 3 | 0 | -14.0 | 9.6 | 0.73 |
| 24 | 42.0 | 1.5 | 1.1 | 42.3 | 0.9 | 3 | 0 | -10.0 | 9.6 | 0.52 |
| 26 | 55.7 | 0.0 | 0.0 | 51.9 | 3.8 | 3 | 0 | 3.7 | 9.6 | 0.19 |
| 27 | 39.8 | 6.6 | 4.8 | 39.5 | 2.8 | 3 | 0 | -12.2 | 9.6 | 0.64 |
| 29 | 49.4 | 0.6 | 0.4 | 46.6 | 3.0 | 3 | 0 | -2.6 | 9.6 | 0.14 |
| 30 | 55.5 | 4.9 | 3.5 | 55.6 | 2.0 | 3 | 0 | 3.5 | 9.6 | 0.18 |
| 31 | 52.0 | 0.0 | 0.0 | 55.7 | 3.7 | 3 | 0 | 0.0 | 9.6 | 0.00 |
| 32 | 39.6 | 0.7 | 0.5 | 39.4 | 0.4 | 3 | 0 | -12.4 | 9.6 | 0.65 |
| 33 | 53.0 | 1.5 | 1.1 | 53.3 | 0.9 | 3 | 0 | 1.0 | 9.6 | 0.05 |
| 34 | 63.2 | 8.3 | 6.0 | 58.5 | 7.7 | 3 | 0 | 11.2 | 9.6 | 0.58 |
| 35 | 36.0 | 10.4 | 7.5 | 37.7 | 5.5 | 3 | 0 | -16.0 | 9.6 | 0.83 |
| 36 | 39.8 | 0.3 | 0.2 | 39.3 | 0.6 | 3 | 0 | -12.2 | 9.6 | 0.64 |
| 37 | 35.2 | 1.3 | 0.9 | 35.3 | 0.7 | 3 | 0 | -16.9 | 9.6 | 0.88 |
| 38 | 91.0 | 4.4 | 3.2 | 82.7 | 9.9 | 3 | 0 | 39.0 | 9.6 | 2.03 |
| 39 | 57.3 | 2.4 | 1.7 | 57.4 | 1.0 | 3 | 0 | 5.3 | 9.6 | 0.28 |
| 40 | 42.5 | 0.0 | 0.0 | 43.7 | 1.2 | 3 | 0 | -9.5 | 9.6 | 0.49 |
| 41 | 52.5 | 3.0 | 2.1 | 53.5 | 2.0 | 3 | 0 | 0.5 | 9.6 | 0.03 |
| 42 | 56.6 | 1.6 | 1.2 | 56.2 | 1.0 | 3 | 0 | 4.6 | 9.6 | 0.24 |
| 43 | 31.8 | 10.7 | 7.7 | 23.6 | 12.0 | 3 | 1 | -20.2 | 9.6 | 1.05 |
| 44 | 59.0 | 3.0 | 2.1 | 57.3 | 2.7 | 3 | 0 | 7.0 | 9.6 | 0.36 |
| 45 | 53.6 | 0.0 | 0.0 | 53.5 | 0.1 | 3 | 0 | 1.6 | 9.6 | 0.08 |
| 46 | 49.4 | 3.4 | 2.5 | 49.7 | 1.6 | 3 | 0 | -2.6 | 9.6 | 0.14 |
| 50 | 38.0 | 4.4 | 3.2 | 37.7 | 2.0 | 3 | 0 | -14.0 | 9.6 | 0.73 |
| 52 | 60.0 | 1.5 | 1.1 | 59.0 | 1.5 | 3 | 0 | 8.0 | 9.6 | 0.42 |
| 53 | 59.3 | 4.9 | 3.5 | 59.2 | 2.0 | 3 | 0 | 7.3 | 9.6 | 0.38 |
| 54 | 8.8 | 1.1 | 0.8 | 8.9 | 0.5 | 3 | 0 | -43.2 | 9.6 | 2.25 |
| **Tryptophan** | | | | | | | | | | |
| 56 | 45.0 | 2.4 | 1.7 | 45.4 | 1.3 | 3 | 0 | -7.0 | 9.6 | 0.36 |
| 57 | 53.3 | 0.7 | 0.5 | 52.9 | 0.7 | 3 | 0 | 1.3 | 9.6 | 0.07 |
| 58 | 52.7 | 0.4 | 0.3 | 53.0 | 0.5 | 3 | 0 | 0.7 | 9.6 | 0.04 |
| 59 | 51.0 | 0.0 | 0.0 | 50.0 | 1.0 | 3 | 0 | -1.0 | 9.6 | 0.05 |
| 60 | 54.0 | 1.5 | 1.1 | 53.3 | 1.2 | 3 | 0 | 2.0 | 9.6 | 0.10 |
| 61 | 0.0 | 0.0 | 0.0 | 0.0 | 0.0 | 3 | 1 | -52.0 | 9.6 | 2.71 |
| 62 | 42.0 | 1.5 | 1.1 | 43.0 | 1.5 | 3 | 0 | -10.0 | 9.6 | 0.52 |
| 64 | 69.0 | 0.0 | 0.0 | 69.3 | 0.3 | 3 | 0 | 17.0 | 9.6 | 0.88 |
| 65 | 72.3 | 1.2 | 0.9 | 76.2 | 4.3 | 3 | 0 | 20.3 | 9.6 | 1.06 |
| 66 | 51.8 | 1.2 | 0.8 | 52.8 | 1.4 | 3 | 0 | -0.2 | 9.6 | 0.01 |
| 67 | 38.7 | 0.1 | 0.1 | 36.4 | 2.4 | 3 | 0 | -13.3 | 9.6 | 0.69 |
| 69 | 58.1 | 1.0 | 0.7 | 58.5 | 0.7 | 3 | 0 | 6.1 | 9.6 | 0.32 |
| 70 | 47.0 | 0.0 | 0.0 | 46.0 | 1.0 | 3 | 0 | -5.0 | 9.6 | 0.26 |
| 71 | 59.0 | 4.4 | 3.9 | 59.0 | 3.0 | 2 | NA | 7.0 | 9.2 | 0.38 |
| 72 | 52.0 | 1.5 | 1.1 | 53.0 | 1.5 | 3 | 0 | 0.0 | 9.6 | 0.00 |
| 73 | 45.0 | 0.0 | 0.0 | 45.7 | 0.7 | 3 | 0 | -7.0 | 9.6 | 0.36 |
| 74 | 58.1 | 1.2 | 0.9 | 58.6 | 0.9 | 3 | 0 | 6.1 | 9.6 | 0.32 |
| 75 | 53.1 | 8.0 | 5.8 | 53.0 | 3.2 | 3 | 0 | 1.1 | 9.6 | 0.06 |
| 76 | 55.1 | 2.5 | 1.8 | 54.7 | 1.3 | 3 | 0 | 3.1 | 9.6 | 0.16 |
| 77 | 45.0 | 5.9 | 4.3 | 45.0 | 2.3 | 3 | 0 | -7.0 | 9.6 | 0.36 |
| 78 | 45.0 | 4.4 | 3.2 | 45.3 | 2.0 | 3 | 0 | -7.0 | 9.6 | 0.36 |
| 79 | 0.0 | 0.0 | 0.0 | 0.0 | 0.0 | 3 | 1 | -52.0 | 9.6 | 2.71 |
| 80 | 0.0 | 0.0 | 0.0 | 0.0 | 0.0 | 3 | 1 | -52.0 | 9.6 | 2.71 |
| 81 | 60.2 | 1.6 | 1.2 | 60.1 | 0.8 | 3 | 0 | 8.2 | 9.6 | 0.43 |
| 83 | 57.0 | 1.0 | 0.8 | 57.9 | 1.3 | 3 | 0 | 5.0 | 9.6 | 0.26 |
| 84 | 50.0 | 0.6 | 0.4 | 50.4 | 0.7 | 3 | 0 | -2.0 | 9.6 | 0.11 |
| 85 | 53.0 | 4.4 | 3.2 | 52.3 | 2.3 | 3 | 0 | 1.0 | 9.6 | 0.05 |
| 86 | 55.0 | 1.5 | 1.1 | 54.7 | 0.9 | 3 | 0 | 3.0 | 9.6 | 0.16 |
| 88 | 50.0 | 0.0 | 0.0 | 50.6 | 0.6 | 3 | 0 | -2.0 | 9.6 | 0.10 |
| 90 | 60.5 | 0.1 | 0.1 | 60.7 | 0.2 | 3 | 0 | 8.5 | 9.6 | 0.44 |

**Tyrosine**

| **Lab** | **Median** | **MAD** | **u (median)** | **Mean** | **u (mean)** | **n** | **Non-detects** | **DoE** | **u (DoE)** | **Score** |
| --- | --- | --- | --- | --- | --- | --- | --- | --- | --- | --- |
| 1 | 71.8 | 1.3 | 1.0 | 70.9 | 1.3 | 3 | 0 | 6.0 | 3.8 | 0.78 |
| 2 | 65.8 | 2.1 | 1.5 | 65.8 | 0.8 | 3 | 0 | 0.0 | 3.8 | 0.00 |
| 3 | 63.0 | 0.0 | 0.0 | 65.0 | 2.0 | 3 | 0 | -2.8 | 3.8 | 0.36 |
| 4 | 64.7 | 1.8 | 1.3 | 64.1 | 1.2 | 3 | 0 | -1.1 | 3.8 | 0.14 |
| 5 | 63.0 | 1.5 | 1.1 | 63.3 | 0.9 | 3 | 0 | -2.8 | 3.8 | 0.36 |
| 6 | 66.0 | 0.0 | 0.0 | 66.3 | 0.3 | 3 | 0 | 0.2 | 3.8 | 0.03 |
| 7 | 66.0 | 1.5 | 1.1 | 67.0 | 1.5 | 3 | 0 | 0.2 | 3.8 | 0.03 |
| 8 | 60.0 | 0.0 | 0.0 | 59.7 | 0.3 | 3 | 0 | -5.8 | 3.8 | 0.75 |
| 9 | 77.6 | 27.6 | 20.0 | 93.7 | 25.9 | 3 | 0 | 11.8 | 3.8 | 1.54 |
| 10 | 63.5 | 0.7 | 0.5 | 62.9 | 0.9 | 3 | 0 | -2.3 | 3.8 | 0.30 |
| 11 | 58.0 | 3.7 | 2.7 | 62.2 | 5.5 | 3 | 0 | -7.8 | 3.8 | 1.01 |
| **Tyrosine** | | | | | | | | | | |
| 12 | 67.7 | 2.5 | 1.8 | 68.2 | 1.5 | 3 | 0 | 1.9 | 3.8 | 0.25 |
| 13 | 69.0 | 1.5 | 1.1 | 68.7 | 0.9 | 3 | 0 | 3.2 | 3.8 | 0.42 |
| 14 | 67.0 | 0.0 | 0.0 | 66.7 | 0.3 | 3 | 0 | 1.2 | 3.8 | 0.16 |
| 15 | 63.2 | 0.8 | 0.6 | 63.2 | 0.3 | 3 | 0 | -2.6 | 3.8 | 0.34 |
| 16 | 66.0 | 0.0 | 0.0 | 66.7 | 0.7 | 3 | 0 | 0.2 | 3.8 | 0.03 |
| 17 | 67.0 | 1.5 | 1.1 | 69.0 | 2.5 | 3 | 0 | 1.2 | 3.8 | 0.16 |
| 18 | 67.0 | 4.4 | 3.2 | 69.3 | 3.9 | 3 | 0 | 1.2 | 3.8 | 0.16 |
| 19 | 73.4 | 0.1 | 0.1 | 72.1 | 1.3 | 3 | 0 | 7.6 | 3.8 | 0.98 |
| 20 | 63.0 | 3.0 | 2.1 | 65.0 | 3.1 | 3 | 0 | -2.8 | 3.8 | 0.36 |
| 21 | 72.0 | 1.5 | 1.1 | 72.0 | 0.6 | 3 | 0 | 6.2 | 3.8 | 0.81 |
| 22 | 69.0 | 0.0 | 0.0 | 68.7 | 0.3 | 3 | 0 | 3.2 | 3.8 | 0.42 |
| 23 | 55.5 | 0.9 | 0.6 | 55.3 | 0.6 | 3 | 0 | -10.3 | 3.8 | 1.34 |
| 24 | 60.0 | 1.5 | 1.1 | 61.0 | 1.5 | 3 | 0 | -5.8 | 3.8 | 0.75 |
| 25 | 68.0 | 0.0 | 0.0 | 68.0 | 0.0 | 3 | 0 | 2.2 | 3.8 | 0.29 |
| 26 | 57.2 | 0.2 | 0.1 | 57.5 | 0.3 | 3 | 0 | -8.6 | 3.8 | 1.12 |
| 27 | 68.5 | 0.3 | 0.2 | 68.0 | 0.6 | 3 | 0 | 2.7 | 3.8 | 0.35 |
| 28 | 59.0 | 3.0 | 2.1 | 58.7 | 1.5 | 3 | 0 | -6.8 | 3.8 | 0.88 |
| 29 | 60.8 | 1.9 | 1.4 | 60.9 | 0.8 | 3 | 0 | -5.0 | 3.8 | 0.65 |
| 30 | 69.3 | 6.5 | 4.7 | 69.6 | 2.8 | 3 | 0 | 3.5 | 3.8 | 0.46 |
| 31 | 73.0 | 3.0 | 2.1 | 77.0 | 5.0 | 3 | 0 | 7.2 | 3.8 | 0.94 |
| 32 | 65.3 | 0.0 | 0.0 | 65.9 | 0.6 | 3 | 0 | -0.5 | 3.8 | 0.07 |
| 33 | 66.0 | 1.5 | 1.1 | 66.0 | 0.6 | 3 | 0 | 0.2 | 3.8 | 0.03 |
| 34 | 63.0 | 3.4 | 2.5 | 61.5 | 2.7 | 3 | 0 | -2.8 | 3.8 | 0.36 |
| 35 | 63.0 | 3.0 | 2.1 | 63.7 | 1.8 | 3 | 0 | -2.8 | 3.8 | 0.36 |
| 36 | 62.8 | 0.9 | 0.6 | 63.4 | 0.9 | 3 | 0 | -3.0 | 3.8 | 0.39 |
| 37 | 64.8 | 0.2 | 0.1 | 64.0 | 0.8 | 3 | 0 | -1.0 | 3.8 | 0.13 |
| 38 | 67.0 | 1.5 | 1.1 | 68.0 | 1.5 | 3 | 0 | 1.2 | 3.8 | 0.16 |
| 39 | 62.0 | 0.1 | 0.1 | 62.4 | 0.5 | 3 | 0 | -3.8 | 3.8 | 0.49 |
| 40 | 69.5 | 0.7 | 0.5 | 69.8 | 0.6 | 3 | 0 | 3.7 | 3.8 | 0.48 |
| 41 | 61.2 | 0.4 | 0.3 | 61.8 | 0.8 | 3 | 0 | -4.6 | 3.8 | 0.60 |
| 42 | 68.0 | 0.3 | 0.2 | 67.1 | 1.0 | 3 | 0 | 2.2 | 3.8 | 0.29 |
| 43 | 57.5 | 0.0 | 0.0 | 56.9 | 0.6 | 3 | 0 | -8.3 | 3.8 | 1.09 |
| 44 | 67.0 | 0.0 | 0.0 | 68.3 | 1.3 | 3 | 0 | 1.2 | 3.8 | 0.16 |
| 45 | 64.7 | 2.2 | 1.6 | 64.1 | 1.4 | 3 | 0 | -1.1 | 3.8 | 0.14 |
| 46 | 71.9 | 0.1 | 0.1 | 71.2 | 0.8 | 3 | 0 | 6.1 | 3.8 | 0.79 |
| 47 | 68.0 | 0.0 | 0.0 | 67.0 | 1.0 | 3 | 0 | 2.2 | 3.8 | 0.29 |
| 48 | 71.4 | 8.2 | 5.9 | 68.8 | 5.6 | 3 | 0 | 5.6 | 3.8 | 0.73 |
| 49 | 70.1 | 2.5 | 1.8 | 71.1 | 1.9 | 3 | 0 | 4.3 | 3.8 | 0.56 |
| 50 | 63.0 | 1.5 | 1.1 | 61.7 | 1.9 | 3 | 0 | -2.8 | 3.8 | 0.36 |
| 51 | 63.0 | 1.5 | 1.1 | 63.0 | 0.6 | 3 | 0 | -2.8 | 3.8 | 0.36 |
| 52 | 64.0 | 3.0 | 2.1 | 65.7 | 2.7 | 3 | 0 | -1.8 | 3.8 | 0.23 |
| 53 | 81.6 | 4.9 | 3.5 | 79.4 | 3.9 | 3 | 0 | 15.8 | 3.8 | 2.06 |
| 54 | 61.7 | 0.4 | 0.3 | 62.3 | 0.8 | 3 | 0 | -4.2 | 3.8 | 0.54 |
| 55 | 65.0 | 0.0 | 0.0 | 64.7 | 0.3 | 3 | 0 | -0.8 | 3.8 | 0.10 |
| 56 | 62.4 | 2.1 | 1.5 | 62.0 | 1.1 | 3 | 0 | -3.4 | 3.8 | 0.44 |
| **Tyrosine** | | | | | | | | | | |
| 57 | 66.6 | 1.3 | 1.0 | 66.6 | 0.5 | 3 | 0 | 0.8 | 3.8 | 0.10 |
| 58 | 68.5 | 0.0 | 0.0 | 67.7 | 0.8 | 3 | 0 | 2.7 | 3.8 | 0.35 |
| 59 | 64.0 | 0.0 | 0.0 | 63.3 | 0.7 | 3 | 0 | -1.8 | 3.8 | 0.23 |
| 60 | 66.0 | 3.0 | 2.1 | 66.7 | 1.8 | 3 | 0 | 0.2 | 3.8 | 0.03 |
| 61 | 58.4 | 0.4 | 0.3 | 58.5 | 0.2 | 3 | 0 | -7.4 | 3.8 | 0.96 |
| 62 | 65.0 | 0.0 | 0.0 | 67.3 | 2.3 | 3 | 0 | -0.8 | 3.8 | 0.10 |
| 63 | 65.5 | 0.3 | 0.2 | 65.6 | 0.2 | 3 | 0 | -0.3 | 3.8 | 0.04 |
| 64 | 63.0 | 1.5 | 1.1 | 62.0 | 1.5 | 3 | 0 | -2.8 | 3.8 | 0.36 |
| 65 | 70.1 | 0.2 | 0.1 | 69.9 | 0.2 | 3 | 0 | 4.3 | 3.8 | 0.55 |
| 66 | 75.5 | 2.5 | 1.8 | 75.6 | 1.1 | 3 | 0 | 9.7 | 3.8 | 1.26 |
| 67 | 68.9 | 1.5 | 1.1 | 66.3 | 3.1 | 3 | 0 | 3.1 | 3.8 | 0.40 |
| 68 | 71.2 | 3.3 | 2.4 | 72.7 | 2.7 | 3 | 0 | 5.4 | 3.8 | 0.70 |
| 69 | 73.0 | 1.7 | 1.3 | 72.7 | 1.0 | 3 | 0 | 7.2 | 3.8 | 0.93 |
| 70 | 64.0 | 1.5 | 1.1 | 63.3 | 1.2 | 3 | 0 | -1.8 | 3.8 | 0.23 |
| 71 | 61.0 | 3.0 | 2.1 | 62.0 | 2.1 | 3 | 0 | -4.8 | 3.8 | 0.62 |
| 72 | 64.0 | 0.0 | 0.0 | 63.7 | 0.3 | 3 | 0 | -1.8 | 3.8 | 0.23 |
| 73 | 64.0 | 1.5 | 1.1 | 64.0 | 0.6 | 3 | 0 | -1.8 | 3.8 | 0.23 |
| 74 | 71.7 | 0.9 | 0.6 | 71.1 | 0.9 | 3 | 0 | 5.9 | 3.8 | 0.77 |
| 75 | 69.5 | 0.6 | 0.4 | 68.8 | 0.9 | 3 | 0 | 3.7 | 3.8 | 0.48 |
| 76 | 71.6 | 1.3 | 1.0 | 73.3 | 2.2 | 3 | 0 | 5.8 | 3.8 | 0.75 |
| 77 | 64.0 | 1.5 | 1.1 | 62.7 | 1.9 | 3 | 0 | -1.8 | 3.8 | 0.23 |
| 78 | 62.0 | 3.0 | 2.1 | 62.3 | 1.5 | 3 | 0 | -3.8 | 3.8 | 0.49 |
| 79 | 67.0 | 0.0 | 0.0 | 66.7 | 0.3 | 3 | 0 | 1.2 | 3.8 | 0.16 |
| 80 | 42.0 | 1.5 | 1.1 | 41.3 | 1.2 | 3 | 0 | -23.8 | 3.8 | 3.10 |
| 81 | 68.0 | 1.3 | 1.0 | 67.8 | 0.7 | 3 | 0 | 2.2 | 3.8 | 0.29 |
| 83 | 64.0 | 0.7 | 0.5 | 63.6 | 0.7 | 3 | 0 | -1.8 | 3.8 | 0.23 |
| 84 | 64.7 | 4.7 | 3.4 | 64.7 | 1.8 | 3 | 0 | -1.1 | 3.8 | 0.15 |
| 85 | 65.0 | 7.4 | 5.4 | 64.3 | 3.5 | 3 | 0 | -0.8 | 3.8 | 0.10 |
| 86 | 68.0 | 1.5 | 1.1 | 69.3 | 1.9 | 3 | 0 | 2.2 | 3.8 | 0.29 |
| 87 | 66.0 | 0.0 | 0.0 | 66.0 | 0.0 | 3 | 0 | 0.2 | 3.8 | 0.03 |
| 88 | 69.5 | 1.1 | 0.8 | 69.5 | 0.5 | 3 | 0 | 3.7 | 3.8 | 0.48 |
| 89 | 64.0 | 3.0 | 2.1 | 63.0 | 2.1 | 3 | 0 | -1.8 | 3.8 | 0.23 |
| 90 | 68.6 | 0.4 | 0.3 | 68.6 | 0.2 | 3 | 0 | 2.8 | 3.8 | 0.36 |

**Valine**

| **Lab** | **Median** | **MAD** | **u (median)** | **Mean** | **u (mean)** | **n** | **Non-detects** | **DoE** | **u (DoE)** | **Score** |
| --- | --- | --- | --- | --- | --- | --- | --- | --- | --- | --- |
| 1 | 235.3 | 0.6 | 0.4 | 237.6 | 2.5 | 3 | 0 | 1.3 | 11.0 | 0.06 |
| 2 | 231.0 | 3.0 | 2.1 | 229.7 | 2.4 | 3 | 0 | -3.0 | 11.0 | 0.14 |
| 3 | 231.0 | 1.5 | 1.1 | 237.3 | 6.8 | 3 | 0 | -3.0 | 11.0 | 0.14 |
| 4 | 230.0 | 1.5 | 1.1 | 231.3 | 1.9 | 3 | 0 | -4.0 | 11.0 | 0.18 |
| 5 | 234.0 | 1.5 | 1.1 | 225.0 | 9.5 | 3 | 0 | 0.0 | 11.0 | 0.00 |
| 6 | 231.0 | 1.5 | 1.1 | 232.3 | 1.9 | 3 | 0 | -3.0 | 11.0 | 0.14 |
| 7 | 242.0 | 4.4 | 3.2 | 232.7 | 10.9 | 3 | 0 | 8.0 | 11.0 | 0.36 |
| 8 | 231.0 | 3.0 | 2.1 | 229.3 | 2.7 | 3 | 0 | -3.0 | 11.0 | 0.14 |
| **Valine** | | | | | | | | | | |
| 9 | 224.6 | 31.0 | 22.4 | 274.3 | 60.4 | 3 | 0 | -9.4 | 11.0 | 0.43 |
| 10 | 231.9 | 7.2 | 5.2 | 229.4 | 5.1 | 3 | 0 | -2.1 | 11.0 | 0.10 |
| 11 | 245.0 | 4.4 | 3.2 | 246.0 | 2.6 | 3 | 0 | 11.0 | 11.0 | 0.50 |
| 12 | 230.9 | 1.3 | 1.0 | 236.0 | 5.5 | 3 | 0 | -3.1 | 11.0 | 0.14 |
| 13 | 251.0 | 3.0 | 2.1 | 250.3 | 1.8 | 3 | 0 | 17.0 | 11.0 | 0.77 |
| 14 | 232.0 | 3.0 | 2.1 | 232.3 | 1.5 | 3 | 0 | -2.0 | 11.0 | 0.09 |
| 15 | 232.0 | 2.7 | 1.9 | 230.9 | 2.1 | 3 | 0 | -2.0 | 11.0 | 0.09 |
| 16 | 232.0 | 3.0 | 2.1 | 232.0 | 1.2 | 3 | 0 | -2.0 | 11.0 | 0.09 |
| 17 | 232.0 | 0.0 | 0.0 | 236.3 | 4.3 | 3 | 0 | -2.0 | 11.0 | 0.09 |
| 18 | 262.0 | 4.4 | 3.2 | 258.3 | 5.2 | 3 | 0 | 28.0 | 11.0 | 1.28 |
| 19 | 237.8 | 10.9 | 7.9 | 238.5 | 4.9 | 3 | 0 | 3.8 | 11.0 | 0.17 |
| 20 | 224.9 | 9.0 | 6.5 | 227.6 | 6.0 | 3 | 0 | -9.1 | 11.0 | 0.41 |
| 21 | 252.0 | 4.4 | 3.2 | 249.3 | 4.3 | 3 | 0 | 18.0 | 11.0 | 0.82 |
| 22 | 258.0 | 0.0 | 0.0 | 258.0 | 0.0 | 3 | 0 | 24.0 | 11.0 | 1.09 |
| 23 | 237.4 | 3.0 | 2.1 | 236.5 | 2.0 | 3 | 0 | 3.4 | 11.0 | 0.15 |
| 24 | 233.0 | 1.5 | 1.1 | 237.3 | 4.8 | 3 | 0 | -1.0 | 11.0 | 0.05 |
| 25 | 244.0 | 1.5 | 1.1 | 245.3 | 1.9 | 3 | 0 | 10.0 | 11.0 | 0.46 |
| 26 | 209.9 | 2.1 | 1.5 | 207.6 | 3.1 | 3 | 0 | -24.1 | 11.0 | 1.10 |
| 27 | 246.5 | 4.1 | 3.0 | 245.7 | 2.3 | 3 | 0 | 12.5 | 11.0 | 0.57 |
| 28 | 209.0 | 25.2 | 18.2 | 209.0 | 9.8 | 3 | 0 | -25.0 | 11.0 | 1.14 |
| 29 | 219.2 | 5.3 | 3.9 | 220.5 | 3.3 | 3 | 0 | -14.8 | 11.0 | 0.67 |
| 30 | 246.8 | 21.8 | 15.8 | 247.7 | 9.3 | 3 | 0 | 12.8 | 11.0 | 0.58 |
| 31 | 236.0 | 3.0 | 2.1 | 238.0 | 3.1 | 3 | 0 | 2.0 | 11.0 | 0.09 |
| 32 | 269.9 | 3.0 | 2.1 | 269.2 | 1.8 | 3 | 0 | 35.9 | 11.0 | 1.63 |
| 33 | 226.0 | 0.0 | 0.0 | 228.0 | 2.0 | 3 | 0 | -8.0 | 11.0 | 0.36 |
| 34 | 222.0 | 1.5 | 1.1 | 218.3 | 4.2 | 3 | 0 | -12.0 | 11.0 | 0.55 |
| 35 | 240.0 | 4.4 | 3.2 | 240.0 | 1.7 | 3 | 0 | 6.0 | 11.0 | 0.27 |
| 36 | 263.2 | 5.7 | 4.1 | 263.2 | 2.2 | 3 | 0 | 29.2 | 11.0 | 1.33 |
| 37 | 225.9 | 0.4 | 0.3 | 222.3 | 3.7 | 3 | 0 | -8.1 | 11.0 | 0.37 |
| 38 | 244.0 | 0.0 | 0.0 | 245.3 | 1.3 | 3 | 0 | 10.0 | 11.0 | 0.46 |
| 39 | 215.0 | 8.9 | 6.4 | 214.3 | 4.1 | 3 | 0 | -19.0 | 11.0 | 0.87 |
| 40 | 248.0 | 6.7 | 4.8 | 249.5 | 4.0 | 3 | 0 | 14.0 | 11.0 | 0.64 |
| 41 | 227.2 | 2.8 | 2.0 | 225.2 | 3.0 | 3 | 0 | -6.8 | 11.0 | 0.31 |
| 42 | 246.2 | 5.0 | 3.6 | 243.9 | 4.1 | 3 | 0 | 12.2 | 11.0 | 0.56 |
| 43 | 201.1 | 2.4 | 1.7 | 201.9 | 1.7 | 3 | 0 | -32.9 | 11.0 | 1.50 |
| 44 | 264.0 | 0.0 | 0.0 | 267.3 | 3.3 | 3 | 0 | 30.0 | 11.0 | 1.37 |
| 45 | 240.7 | 1.0 | 0.8 | 237.2 | 3.9 | 3 | 0 | 6.7 | 11.0 | 0.31 |
| 46 | 230.8 | 5.6 | 4.1 | 229.0 | 3.9 | 3 | 0 | -3.2 | 11.0 | 0.15 |
| 47 | 222.0 | 1.5 | 1.1 | 219.3 | 3.2 | 3 | 0 | -12.0 | 11.0 | 0.55 |
| 48 | 257.8 | 3.0 | 2.1 | 253.7 | 5.2 | 3 | 0 | 23.8 | 11.0 | 1.08 |
| 49 | 266.0 | 1.5 | 1.1 | 266.0 | 0.6 | 3 | 0 | 32.0 | 11.0 | 1.46 |
| 50 | 226.0 | 0.0 | 0.0 | 222.0 | 4.0 | 3 | 0 | -8.0 | 11.0 | 0.36 |
| 51 | 241.0 | 1.5 | 1.1 | 241.0 | 0.6 | 3 | 0 | 7.0 | 11.0 | 0.32 |
| 52 | 234.0 | 1.5 | 1.1 | 240.7 | 7.2 | 3 | 0 | 0.0 | 11.0 | 0.00 |
| 53 | 215.3 | 16.0 | 11.6 | 215.3 | 6.3 | 3 | 0 | -18.7 | 11.0 | 0.85 |
| **Valine** | | | | | | | | | | |
| 54 | 235.4 | 1.4 | 1.0 | 238.8 | 3.8 | 3 | 0 | 1.4 | 11.0 | 0.06 |
| 55 | 232.0 | 1.5 | 1.1 | 232.0 | 0.6 | 3 | 0 | -2.0 | 11.0 | 0.09 |
| 56 | 223.0 | 6.2 | 4.5 | 222.1 | 3.3 | 3 | 0 | -11.0 | 11.0 | 0.50 |
| 57 | 236.4 | 4.2 | 3.0 | 235.8 | 2.2 | 3 | 0 | 2.4 | 11.0 | 0.11 |
| 58 | 228.7 | 4.7 | 3.4 | 229.6 | 2.6 | 3 | 0 | -5.3 | 11.0 | 0.24 |
| 59 | 230.0 | 3.0 | 2.1 | 227.3 | 3.7 | 3 | 0 | -4.0 | 11.0 | 0.18 |
| 60 | 245.0 | 3.0 | 2.1 | 248.7 | 4.7 | 3 | 0 | 11.0 | 11.0 | 0.50 |
| 61 | 230.7 | 1.0 | 0.8 | 230.8 | 0.5 | 3 | 0 | -3.3 | 11.0 | 0.15 |
| 62 | 234.0 | 4.4 | 3.2 | 234.0 | 1.7 | 3 | 0 | 0.0 | 11.0 | 0.00 |
| 63 | 234.0 | 1.3 | 1.0 | 234.3 | 0.8 | 3 | 0 | 0.0 | 11.0 | 0.00 |
| 64 | 219.0 | 3.0 | 2.1 | 215.0 | 5.0 | 3 | 0 | -15.0 | 11.0 | 0.68 |
| 65 | 265.5 | 12.8 | 9.2 | 266.1 | 5.5 | 3 | 0 | 31.5 | 11.0 | 1.43 |
| 66 | 254.0 | 4.4 | 3.2 | 256.0 | 3.6 | 3 | 0 | 20.0 | 11.0 | 0.91 |
| 67 | 236.0 | 5.9 | 4.3 | 229.7 | 8.4 | 3 | 0 | 2.0 | 11.0 | 0.09 |
| 68 | 241.0 | 3.5 | 2.5 | 249.5 | 9.7 | 3 | 0 | 7.0 | 11.0 | 0.32 |
| 69 | 257.5 | 19.4 | 14.1 | 256.4 | 8.5 | 3 | 0 | 23.5 | 11.0 | 1.07 |
| 70 | 227.0 | 8.9 | 6.4 | 219.7 | 10.5 | 3 | 0 | -7.0 | 11.0 | 0.32 |
| 71 | 217.0 | 8.9 | 6.4 | 214.0 | 6.2 | 3 | 0 | -17.0 | 11.0 | 0.77 |
| 72 | 227.0 | 1.5 | 1.1 | 226.0 | 1.5 | 3 | 0 | -7.0 | 11.0 | 0.32 |
| 73 | 228.0 | 3.0 | 2.1 | 226.7 | 2.4 | 3 | 0 | -6.0 | 11.0 | 0.27 |
| 74 | 266.2 | 15.7 | 11.4 | 267.0 | 6.8 | 3 | 0 | 32.2 | 11.0 | 1.47 |
| 75 | 250.9 | 8.9 | 6.4 | 250.4 | 3.9 | 3 | 0 | 16.9 | 11.0 | 0.77 |
| 76 | 271.9 | 1.9 | 1.4 | 266.8 | 5.8 | 3 | 0 | 37.9 | 11.0 | 1.73 |
| 77 | 231.0 | 13.3 | 9.7 | 233.3 | 7.3 | 3 | 0 | -3.0 | 11.0 | 0.14 |
| 78 | 229.0 | 7.4 | 5.4 | 230.3 | 4.1 | 3 | 0 | -5.0 | 11.0 | 0.23 |
| 79 | 221.0 | 4.4 | 3.2 | 223.0 | 3.6 | 3 | 0 | -13.0 | 11.0 | 0.59 |
| 80 | 139.0 | 1.5 | 1.1 | 143.3 | 4.8 | 3 | 0 | -95.0 | 11.0 | 4.33 |
| 81 | 236.7 | 0.6 | 0.4 | 235.0 | 1.9 | 3 | 0 | 2.7 | 11.0 | 0.12 |
| 83 | 213.2 | 3.6 | 2.6 | 213.3 | 1.5 | 3 | 0 | -20.8 | 11.0 | 0.95 |
| 84 | 229.9 | 18.1 | 13.1 | 230.5 | 7.6 | 3 | 0 | -4.1 | 11.0 | 0.19 |
| 85 | 232.0 | 4.4 | 3.2 | 244.7 | 14.2 | 3 | 0 | -2.0 | 11.0 | 0.09 |
| 86 | 250.3 | 8.5 | 6.1 | 248.8 | 4.7 | 3 | 0 | 16.3 | 11.0 | 0.74 |
| 87 | 236.0 | 4.4 | 3.2 | 237.0 | 2.6 | 3 | 0 | 2.0 | 11.0 | 0.09 |
| 88 | 247.6 | 0.1 | 0.1 | 240.7 | 6.9 | 3 | 0 | 13.6 | 11.0 | 0.62 |
| 89 | 244.0 | 5.9 | 4.3 | 237.7 | 8.4 | 3 | 0 | 10.0 | 11.0 | 0.46 |
| 90 | 237.8 | 7.6 | 5.5 | 236.4 | 4.2 | 3 | 0 | 3.8 | 11.0 | 0.17 |

**S5: Supplemental table 3: Comparison of study data to desirable analytical performance specifications**

| Analyte | Desirable bias (Corte et al) | Mean bias from consensus value | Desirable Imprecision (Corte et al) | Mean intra-laboratory %CV | PRSD_R_ | Inter-laboratory %CV |
| --- | --- | --- | --- | --- | --- | --- |
| 1-methylhistidine | - | - | - | 3.5 | - | - |
| 3-methylhistidine | - | - | - | 9.1 | - | - |
| Alanine | 14.4 | 5.8 | 7.3 | 2.4 | 9.4 | 5.0 |
| Anserine | - | - | - | - | - | - |
| Arginine | 9.8 | 9.9 | 9.7 | 7.1 | 11.3 | 10.0 |
| Carnosine | - | - | - | - | - | - |
| Citrulline | 12.2 | 10.4 | 10.7 | 4.0 | 12.5 | 11.4 |
| Glutamate | 23.1 | 7.9 | 23.1 | 3.8 | 10.6 | 9.3 |
| Glutamine | 6.3 | 8.5 | 6.0 | 2.9 | 8.5 | 9.1 |
| Glycine | 10.5 | 5.3 | 5.9 | 2.6 | 10.2 | 5.8 |
| Histidine | 7.2 | 8.3 | 4.9 | 3.1 | 10.9 | 8.7 |
| Isoleucine | 12.0 | 7.1 | 7.8 | 3.2 | 11.2 | 7.4 |
| Leucine | 11.6 | 6.5 | 7.4 | 2.3 | 10.2 | 6.9 |
| Lysine | 10.0 | 6.5 | 5.7 | 2.3 | 9.8 | 6.5 |
| Methionine | 11.5 | 10.3 | 7.3 | 3.3 | 13.0 | 8.5 |
| Ornithine | 14.5 | 7.4 | 9.2 | 3.4 | 10.7 | 8.3 |
| Phenylalanine | 10.4 | 4.8 | 4.7 | 2.0 | 8.7 | 5.4 |
| Proline | 26.4 | 7.1 | 10.4 | 3.6 | 9.8 | 7.0 |
| Serine | 11.2 | 6.5 | 6.4 | 3.2 | 11.1 | 7.3 |
| Taurine | 13.4 | 8.3 | 15.3 | 3.6 | 12.2 | 6.2 |
| Threonine | 9.4 | 5.3 | 8.9 | 2.7 | 10.4 | 5.4 |
| Tryptophan | 38.6 | 15.3 | 11.4 | 4.2 | 11.2 | 18.6 |
| Tyrosine | 15.5 | 5.6 | 5.2 | 2.4 | 11.0 | 6.3 |
| Valine | 10.4 | 5.1 | 5.3 | 2.8 | 9.7 | 6.0 |
